# Origin of the cell nucleus, mitosis and sex: roles of intracellular coevolution

**DOI:** 10.1186/1745-6150-5-7

**Published:** 2010-02-04

**Authors:** Thomas Cavalier-Smith

**Affiliations:** 1 Department of Zoology, University of Oxford, South Parks Road, Oxford, OX1 3PS, UK

## Abstract

**Background:**

The transition from prokaryotes to eukaryotes was the most radical change in cell organisation since life began, with the largest ever burst of gene duplication and novelty. According to the coevolutionary theory of eukaryote origins, the fundamental innovations were the concerted origins of the endomembrane system and cytoskeleton, subsequently recruited to form the cell nucleus and coevolving mitotic apparatus, with numerous genetic eukaryotic novelties inevitable consequences of this compartmentation and novel DNA segregation mechanism. Physical and mutational mechanisms of origin of the nucleus are seldom considered beyond the long-standing assumption that it involved wrapping pre-existing endomembranes around chromatin. Discussions on the origin of sex typically overlook its association with protozoan entry into dormant walled cysts and the likely simultaneous coevolutionary, not sequential, origin of mitosis and meiosis.

**Results:**

I elucidate nuclear and mitotic coevolution, explaining the origins of dicer and small centromeric RNAs for positionally controlling centromeric heterochromatin, and how 27 major features of the cell nucleus evolved in four logical stages, making both mechanisms and selective advantages explicit: two initial stages (origin of 30 nm chromatin fibres, enabling DNA compaction; and firmer attachment of endomembranes to heterochromatin) protected DNA and nascent RNA from shearing by novel molecular motors mediating vesicle transport, division, and cytoplasmic motility. Then octagonal nuclear pore complexes (NPCs) arguably evolved from COPII coated vesicle proteins trapped in clumps by Ran GTPase-mediated cisternal fusion that generated the fenestrated nuclear envelope, preventing lethal complete cisternal fusion, and allowing passive protein and RNA exchange. Finally, plugging NPC lumens by an FG-nucleoporin meshwork and adopting karyopherins for nucleocytoplasmic exchange conferred compartmentation advantages. These successive changes took place in naked growing cells, probably as indirect consequences of the origin of phagotrophy. The first eukaryote had 1-2 cilia and also walled resting cysts; I outline how encystation may have promoted the origin of meiotic sex. I also explain why many alternative ideas are inadequate.

**Conclusion:**

Nuclear pore complexes are evolutionary chimaeras of endomembrane- and mitosis-related chromatin-associated proteins. The keys to understanding eukaryogenesis are a proper phylogenetic context and understanding organelle coevolution: how innovations in one cell component caused repercussions on others.

**Reviewers:**

This article was reviewed by Anthony Poole, Gáspár Jékely and Eugene Koonin.

## Background

**Figure 1:**
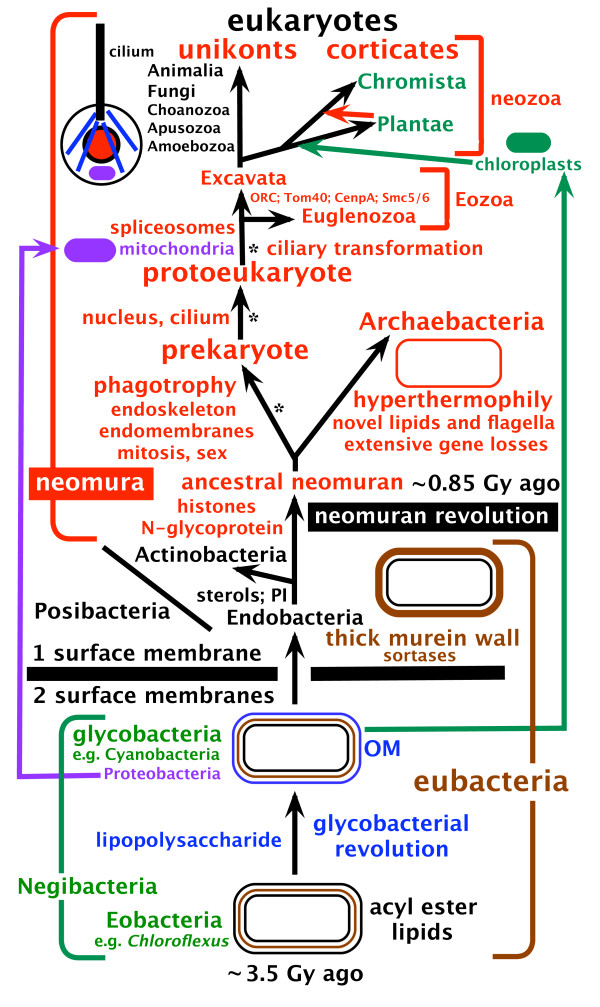
**The tree of life and major steps in cell evolution**. Archaebacteria are sisters to eukaryotes and, contrary to widespread assumptions, the youngest bacterial phylum [6,13]. This tree topology, coupled with extensive losses of posibacterial properties by the ancestral archaebacterium, explains (without lateral gene transfer) how eukaryotes possess a unique combination of properties now seen in archaebacteria, posibacteria and α-proteobacteria. Eukaryote origins in three stages indicated by asterisks probably immediately followed divergence of archaebacteria and eukaryote precursors from the ancestral neomuran. This ancestor arose from a stem actinobacterial posibacterium by a quantum evolutionary shake-up of bacterial organization - the neomuran revolution [6,12]: surface N-linked glycoproteins replaced murein; ribosomes evolved the signal recognition particle's translational arrest domain; histones replaced DNA gyrase, radically changing DNA replication, repair, and transcription enzymes. The eukaryote depicted is a hypothetical early stage after the origin of nucleus, mitochondrion, cilium, and microtubular skeleton but before distinct anterior and posterior cilia and centriolar and ciliary transformation (anterior cilium young, posterior old: [3]) evolved (probably in the cenancestral eukaryote [9]). Kingdom Chromista was recently expanded to include not only the original groups Heterokonta, Cryptista and Haptophyta, but also Alveolata, Rhizaria and Heliozoa [9], making the name chromalveolates now unnecessary. Excavata now exclude Euglenozoa and comprise just three phyla: the ancestrally aerobic Percolozoa and Loukozoa and the ancestrally anaerobic Metamonada (e.g. *Giardia*, *Trichomonas*), which evolved from an aerobic *Malawimonas*-related loukozoan. Sterols and phosphatidylinositol (PI) probably evolved in the ancestral stem actinobacterium but the ancestral hyperthermophilic archaebacterium lost them when isoprenoid ethers replaced acyl ester lipids.

Cells are of only two fundamental kinds: bacteria (=prokaryotes; cells with DNA segregated by surface membrane motors) and eukaryotes (nucleated cells dividing by mitosis) [1,2]. In bacteria the typically single and circular DNA chromosome is attached to the surface cytoplasmic membrane and segregated by protein motors associated with that membrane, and ribosomes start translating messenger RNA (mRNA) even during transcription. Eukaryote chromosomes are normally multiple and linear and never attach directly to the surface plasma membrane. Instead they are fixed to and surrounded by a specialised part of the endomembrane system (the nuclear envelope, NE) during interphase, the part of the cell cycle when the cell grows, genes are transcribed, and DNA replicated. During cell division, by contrast, eukaryotic chromosomes are compacted, precluding transcription or replication, and attach by their centromeres to microtubules of the mitotic spindle, which moves them into daughter cells. The problem of nuclear origins therefore requires understanding coevolution of about 27 cell components (Appendix 1) and how they became functionally interlinked into the fundamentally novel eukaryotic life cycle [3-5], approximately 850 My ago, at least two billion years after bacteria evolved [6]. Not only mitosis, but also sex, i.e. meiosis and syngamy (cell and nuclear fusion), must have evolved at the same time. This conclusion follows irrespective of whether the eukaryote tree is between unikonts (animals, fungi and three protozoan phyla) and bikonts (plants, chromists and all other protozoan phyla [7,8] or is instead between Euglenozoa and all other eukaryotes as shown in Fig. 1 in line with recent arguments for the root lying within Eozoa (Euglenozoa plus excavates), most likely between Euglenozoa and excavates sensu stricto [9]. Peroxisomes, mitochondria, centrioles, cilia, and Golgi dictyosomes must also have originated prior to the last common ancestor of all extant eukaryotes, whichever of these positions of the root is correct [6]. This radical transformation of cell structure (eukaryogenesis) is the most complex and extensive case of quantum evolution in the history of life [2,3,6]. Beforehand earth was a sexless, purely bacterial and viral world. Afterwards sexy, endoskeletal eukaryotes evolved morphological complexity: diatoms, butterflies, corals, whales, kelps, and trees.

Evolution of complex characters typically involves preadaptation, radical mutational innovation, and different selective forces acting in succession [3,6,10]. Here I paint an integrated picture of how the nucleus, sex, and the eukaryotic cell cycle originated and congealed into a novel, unified, and very conservative cellular lifestyle during later stages of the conversion of a bacterium into a eukaryote. In addition to establishing the phylogenetic context (Fig. 1) there are three crucial problems for understanding the origin of the nucleus [5]: (1) assembly of endomembranes around chromatin (the DNA-histone complex); (2) evolution of the nuclear pore complex (NPC), which crucially allows a channel between nucleoplasm and cytoplasm; and (3) origin of centromeres and mitotic spindle, without which nuclear chromosomes cannot be stably inherited. As first argued 30 years ago [11], origin of the cell nucleus cannot be understood in isolation from other major innovations of the eukaryotic cell; intracellular coevolution among different cell constituents that interact physically or that profoundly affect selective forces acting on each other is the key to understanding eukaryote origins [3,4]. Elements of the present synthesis were presented then [11], e.g. that sex began even before the nuclear envelope, i.e. in a prekaryote phase of evolution (see Fig. 1) and the dominant selective advantages. However, the phylogenetic context has changed dramatically with our now much more robust understanding of cell phylogeny (1) [3,7,8,12,13]. Moreover, genomics has enabled molecular origins of many key eukaryotic constituents, including NPCs, to be traced [14-17], whilst advances in molecular cell biology tell us how nuclei actually assemble [18,19] and function. Building on these insights, I now propose the first specific physical mechanism for evolving nuclear envelope architecture and explain its major genetic consequences and why other theories are inadequate.

As the field of eukaryogenesis has been confused by a plethora of contradictory ideas, some not compatible with established evidence, before presenting the novel explanations I summarise two areas to put them in context: (1) the phylogenetic origin of the eukaryotic components, and (2) the origin of the endomembrane system and cytoskeleton. I only outline the conclusions, giving references for details, as most of the evidence and arguments is not new, being already published. Because the nature of molecular changes during major evolutionary transitions is more diversified and complex than some molecular evolutionists have realised, I also preface my original explanations of the origin of the nucleus with an outline of some basic but widely neglected evolutionary principles that apply to all such major innovations in body plan. This background is rather long because the proper evolutionary context is so important: the nucleus did not evolve on its own; explanations of its origin make no sense without understanding the prior evolution of the endomembrane system of which its envelope is a specialised part. Intracellular coevolution of about a 100 novel properties is at the core of understanding eukaryogenesis.

### Phylogenetic context for eukaryogenesis

Eukaryote cells are all evolutionary chimaeras of an ancestrally phagotrophic host cell with nucleus, endomembranes, and endoskeleton [3] and an enslaved α-proteobacterium converted into a mitochondrion close to the time when the nucleus itself originated, i.e. prior to divergence of any extant eukaryotic lineages (Fig. 1) [20]. Contrary to some assumptions [17,21], the host for that symbiogenesis was not an archaebacterium, but an otherwise fully developed early eukaryote with NE and cilium (a protoeukaryote) or else an intermediate stage (prekaryote) that had already evolved rudiments of phagocytosis (the likely means of engulfing the α-proteobacterium) and internal membranes already differentiated into a primitive ER and peroxisomes, endoskeleton, centrosomes and mitosis (see [3,6,20,22] for further explanation). Fig. 1 emphasizes the key importance for early cell evolution of ancestral groups like Posibacteria and Eobacteria that are necessarily paraphyletic (in contrast to derived holophyletic groups like archaebacteria and actinobacteria), but which are phylogenetically perfectly respectable, the 'arguments' against them being fundamentally flawed (see [23]).

Figure 1 differs from many widely discussed views of the tree of life in three major respects: the position of the root of the whole tree, the position of the eukaryotic root, and in the idea that both archaebacteria and eukaryotes evolved from Posibacteria. Though these topics are explained in detail in other papers, many readers may not have assimilated the evidence therein that rather strongly supports them, so I shall begin by outlining the evidence for these interpretations and add a few novel arguments and new evidence for them and explain the flaws in alternative ideas on the rooting and topology of the tree.

### Clade neomura and its posibacterial origin

Archaebacteria are clearly related to the eukaryote host (together forming a clade called neomura [4,12]). But there is no sound evidence that archaebacteria are directly ancestral to eukaryotes. Instead several arguments show they are their sisters [6,12,13]. Thus the >20 features shared by both groups but absent from eubacteria (e.g. N-linked glycoproteins, more complex RNA polymerases, core histones) are not specifically archaebacterial, but neomuran characters that evolved in their common ancestor during the neomuran revolution [4,6,12,13]. Purely archaebacterial characters (notably unique isoprenoid ether lipids and flagella) evolved in the ancestral archaebacterium after it diverged from the prekaryote lineage [12,13]. Moreover, genes shared by eukaryotes and eubacteria, but not archaebacteria (e.g. MreB that became actin [3,6], and eubacterial surface molecules that became NE lamin B receptors [14], and enzymes making acyl ester phospholipids), were probably lost by the ancestral archaebacterium, which apparently underwent massive gene loss during its secondary adaptation to hyperthermophily [12,13]. In addition to those earlier arguments, the most comprehensive multigene analysis to date convincingly places archaebacteria as a holophyletic clade that is sister to eukaryotes, not ancestral to them [24]. However, these authors confusingly refer to the 'deep archaeal origin of eukaryotes' despite their strong evidence that all extant archaebacteria form a derived clade not a paraphyletic ancestral group. The phrase 'archaeal origin' wrongly implies that the common ancestor of eukaryotes and archaebacteria had the specific positive attributes of archaebacteria that distinguish them from both eukaryotes and eubacteria, of which there are very few: notably the isoprenoid ether lipids, archaeosine modified rRNAs, flagella, and duplicate versions of DNA polymerase B [25].

It is unparsimonious to assume that such characters were present in and then lost by the ancestors of eukaryotes. Though the replacement of archaebacterial lipids by acyl ester lipids derived from the enslaved proteobacterial ancestor of mitochondria is a formal possibility [26], it would be evolutionarily extremely onerous and thus unlikely, and phylogeny gives no convincing reason to assume it in the first place. Moreover, the hypothesis of replacement by archaebacterial lipids by eubacterial lipids from the α-proteobacterial symbiont totally fails to explain the origin of phosphatidylinositol, which played a key role in eukaryogenesis [27] and is present in all the actinobacterial relatives of neomura but never in archaebacteria or proteobacteria. Thus, it is far more likely that both archaebacteria and eukaryotes evolved from a common ancestor that was a prokaryote with acyl ester lipids including phosphatidylinositol, but which had not yet evolved either the specifically archaebacterial properties like isoprenoid ether lipids or any eukaryotic properties.

Sterol evolution even more strongly refutes the idea that eukaryotes evolved from archaebacteria and independently shows that neomura are most closely related to actinobacteria. Sterols in actinobacteria and eukaryotes are synthesised from squalene, as are the hopanoids of eubacteria. In all posibacteria squalene is produced from isopentenyl diphosphate (IPP), which is also the precursor for the isoprenoid tails of archaebacterial lipids; in posibacteria, archaebacteria, and eukaryotes that never have plastids (which use instead the cyanobacterial DOX isoprenoid pathway) IPP is generated by the mevalonate synthetic pathway, the enzymes of which were clearly in place and inherited vertically from the last common ancestor of Posibacteria and neomura [28,29]. As the enzymes that convert IPP into sterols are entirely absent from archaebacteria and mostly absent from α-proteobacteria, this simultaneously refutes the popular but totally erroneous ideas that archaebacteria were directly ancestral to eukaryotes [26,30,31] and that eukaryotes got sterols from the enslaved mitochondrion [26,31-33]. Actinobacteria are the only bacteria in which many genes needed for making sterols are phylogenetically widespread and of ancient origin within the group. Sequence trees for four major enzymes of sterol synthesis refute the idea that any of these genes entered actinobacteria by lateral gene transfer [34] and are totally consistent with the vertical descent of sterol biosynthesis from an actinobacterium-like posibacterium to the first eukaryote (and their loss in the ancestral archaebacterium when replacement of acyl esters by isoprenoid ethers provided an alternative and superior means of making membranes more rigid). Oddly, though recognising that their trees rule out lateral transfer from eukaryotes to actinobacteria, Desmond and Gribaldo [34] evade the obvious conclusion that Posibacteria were indeed ancestral to neomura by postulating lateral transfer (LGT) of these genes from a stem pre-eukaryotic lineage into actinobacteria, despite there being no evidence whatever for that implausible and unparsimonious scenario, which would require that Actinobacteria are younger than pre-eukaryotes. The first enzyme of sterol synthesis for squalene monooxygenation (making squalene epoxide) is so widespread in actinobacteria that it must have been present in their last common ancestor [34]; elsewhere in prokaryotes it is known only from a few gamma and delta proteobacteria and one planctomycete (all members of the clade Gracilicutes [13]); as the trees do not require any LGT it probably evolved in the last common ancestor of Posibacteria and Gracilicutes after the prior divergence of Cyanobacteria and the oxygenation of the atmosphere; it is entirely absent from archaebacteria and α-proteobacteria. As sterol synthesis requires oxygen its loss by secondarily or facultatively anaerobic lineages is unsurprising (the likelihood that the ancestral archaebacterium was largely anaerobic [12] is another reason why it lost sterols).

The second enzyme of the sterol synthesis pathway, oxidosqualene cyclase, catalyzing cyclisation of squalene epoxide to make lanosterol and/or cycloartenol is even more widespread in eubacteria, being present in both posibacterial subphyla (Actinobacteria, Endobacteria) as well as Proteobacteria (including even α-proteobacteria), Planctobacteria, and Cyanobacteria, so probably evolved even earlier before Cyanobacteria diverged from the other groups, and was presumably never present in Eobacteria and lost by Sphingobacteria, Spirochaetae, and Archaebacteria. The tree suggests that one planctobacterium (*Stigmatella*) replaced its own enzyme by one from eukaryotes, but gives no evidence for LGTs amongst eubacteria, contrary to the authors assumption [34]. Such replacement by LGT of one enzyme within a pathway is mechanistically simple, but there is no evidence for LGT of the whole pathway at any time in the history of life (by contrast symbiogenetic replacement by whole cell enslavement did allow the mevalonate part of the pathway to be replaced by that of cyanobacteria). The third enzyme in the pathway that catalyses C14 demethylation of lanosterol is known only from the order Actinomycetales (widespread) within Actinobacteria and from one delta and one gamma proteobacterium; as the tree does not support the idea of LGT, most likely it evolved at the same time as the first enzyme but was lost (or evolved beyond bioinformatic recognition) more often. The enzyme DHCR24, which makes the more complex sterols ergosterol and cholesterol, is present widely and phylogenetically deeply in Actinomycetales within Actinobacteria and is sister to its eukaryotic homologue [34] if the tree is rooted between them and the β-proteobacterium *Rhodoferax *in accord with Fig. 1, suggesting that this enzyme also originated at the same time as enzymes one and three but was lost even more often. Homologues were detected in only one other bacterium: *Methylococcus*; its sequence branches well within opisthokonts and was therefore probably acquired by LGT from an animal; however there is no evidence for LGT for that gene provided one roots the tree correctly. The simplest interpretation of the alternative lanosterol and cycloartenol pathways in eukaryotes [35] is that the first eukaryote inherited the posibacterial oxidosqualene cyclase vertically and that it was mutationally modified in plants at the time of origin of plastids and to make cycloartenol preferentially and later transferred to other eukaryotes by secondary symbiogenesis (i.e. to chromists and photosynthetic euglenoids); the sequence tree [34] is consistent with the rooting of eukaryotes within Eozoa (Fig. 1) and refutes my old idea that the plant enzyme came from the cyanobacterial ancestor of plastids [4].

Thus sterol and phosphatidylinositol evolution independently refute the idea that eukaryotes evolved from archaebacteria and both strongly indicate that the closest relatives to neomura are actinobacteria (in agreement with a dozen other characters [12]). However, the evolution of archaebacterial lipids and neomuran glycoproteins suggests that neomura may have evolved from the other posibacterial subphylum, Endobacteria. Homologues of the glycosyl transferases that make N-linked glycoproteins were detectable only in Endobacteria among eubacteria [13] and geranylgeranylglyceryl phosphate synthase (GGGPS) the enzyme that attaches isoprenoid tails to *sn*-Glycerol-1-P to make the membrane lipids of archaebacteria is known only from Endobacteria (specifically Bacillales) and the sphingobacterium *Cytophaga*, making it likely that Endobacteria rather than Actinobacteria were ancestral to neomura. This evidence for an endobacterial origin of neomura can be readily reconciled with the more extensive evidence for their actinobacterial affinities by the posibacterial tree topology of Fig. 1, where Endobacteria are shown as ancestral to both neomura and their sister Actinobacteria. We need only postulate that the cenancestral actinobacterium lost glycosyl transferase and GGGPS after it diverged from neomura and that phosphatidylinositol evolved immediately prior to that bifurcation and was lost only by archaebacteria (together with other acyl esters). This topology also allows the extra 5' Alu domain of the neomuran signal recognition 7SL RNA to have been inherited directly from Endobacteria [12], making it unnecessary to postulate that the positionally equivalent domain present in some Endobacteria (alone among eubacteria) is convergent [13] - assuming that 5' domain was lost in the ancestral actinobacterium. As previously discussed [13], the other key enzyme for the archaebacterial replacement of eubacterial lipids, *sn*-glycerol-1-phosphate dehydrogenase, which makes their unique *sn*-glycerol-1-phosphate, almost certainly evolved from a known posibacterial homologue (also present in *Thermotoga *and Proteobacteria [28,29]). The idea that archaebacterial lipids evolved independently of eubacterial biosynthetic pathways and the idea that their cells evolved independently of eubacterial cells [36,37] are both utter nonsense.

If actinobacteria are holophyletic (Fig. 1), there is also no need to assume that any of the five unique proteins of actinobacteria [38] or any of the actinobacterially unique paralogues of more widespread proteins like the iron uptake regulator Fur [39] were lost by the ancestor of neomura. However, one would have to assume that the most divergent actinobacterial branches had lost 20S proteasomes, as they are restricted to Actinomycetales [13]. Skophammer et al. [40] suggest that archaebacteria plus Endobacteria are a clade because of two claimed shared indels; however, it is evident that one gene pair they considered are not really paralogues and the other is self contradictory [41] so there is no convincing evidence against the topology shown in Figure 1. A quaternary structure argument for dihydroorotate hydrogenase (PyrD) evolution [41] supports a common ancestry for archaebacteria and Endobacteria; but that does not mean that they alone form a clade, for we all accept that the ancestral eukaryote was cladistically closer to Archaebacteria than Endobacteria, so it must have lost the PyrD 1B paralogue; an additional loss by the ancestral actinobacterium reconciles their argument with Fig. 1. An indel argument to exclude the root of the tree of life from Actinobacteria [42] actually excludes it only from the orders Actinomycetales and Bifidobacteriales, as their analysis included no DNA gyrase GyrA proteins from the three most deeply branching orders. But that limitation of the argument does not matter, as there was never any reason to think the root was within Actinobacteria in the first place. My own alignment indicates that the only available GyrA from the deepest branching actinobacterium (*Rubrobacter*) does not have the four amino acid insertion found in other actinobacteria, suggesting that it evolved after the first internal divergence, possibly substantially later (incidentally the insertion region seems incorrectly aligned in [42] and the gap should probably be moved by five amino acids). One cannot use this indel to argue against the topology or rooting of Fig. 1 because when histones evolved in the neomuran ancestor DNA gyrase was replaced by DNA topoisomerase VI [12], whose B subunit probably evolved from GyrB, but whose A subunit is so radically different that they cannot be aligned with GyrA [43,44]. A eukaryote-specific topoisomerase (IIA) probably also evolved from DNA gyrase by fusion of GyrA and GyrB to make a chimaera also so different from its eubacterial ancestors that one cannot apply the indel argument to it. Given the ancestral neomuran transformations of gyrase into novel topoisomerases, the very few archaebacterial GyrAs that can be aligned with those of eubacteria almost certainly entered archaebacteria by LGT from eubacteria [43], so the absence in them of the higher actinobacterial 4-amino acid insertion [42] must not be used to argue against actinobacteria being sisters of neomura (Fig. 1).

The above arguments from eukaryote and archaebacterial lipid evolution strongly contradict (and are more compelling than) a recent 53-gene analysis in which, in contrast to standard phylogenetic methods that show archaebacteria as holophyletic sisters of eukaryotes, a theoretically superior heterogeneous method shows archaebacteria as paraphyletic ancestors to eukaryotes [45]. Several statistically strongly supported branches within eukaryotes on that tree are topologically incorrect, so it cannot safely be concluded (as the authors did) that the grouping of eukaryotes as sisters to crenarchaeotes alone is not also a phylogenetic reconstruction error - as I think is likely. The same problem applies to their analyses of rRNA and 41 protein genes [46]. Virtually all these genes underwent marked episodic accelerated evolution in the eukaryote stem, making them so substantially different from those of archaebacteria that reconstructing the correct tree with confidence is extremely difficult. None of these trees adequately test my thesis about the sister relationship of neomura and actinobacteria as no actinobacteria were included and taxon sampling was generally too sparse to get the best trees. Given that methods and datasets conflict and that the topology within eukaryotes is suspect (and contradictorily different) in all the trees, even though it ought to be easier to reconstruct, I do not share the authors' hope that this type of analysis can establish the historical truth by itself unambiguously enough to be trusted. Comparison of their overall tree with their eukaryotes only tree shows that including the prokaryotic outgroups changes the topology within the eukaryotes and misroots the eukaryote part of the tree. As the stem at the base of neomura is even longer than the stem at the base of eukaryotes it is likely to cause even worse problems of misrooting within the basal neomuran branches. Therefore one can have no confidence in the conclusion of this analysis. The difficulty of deciding by even the best current sequence tree methods whether archaebacteria are holophyletic or paraphyletic emphasizes two things: (1) we should give more weight to other phylogenetic evidence for establishing the correct phylogeny, as I and Lake [40,42,47-51] have, and (2) it is very unlikely that archaebacteria can be several times as old as eukaryotes, as the assumption that archaebacteria are as old as eubacteria (e.g. [36]) would require given the fossil evidence that eubacteria are at least 2.5 and more likely 3-4 times older than eukaryotes [6]; if archaebacteria were really 2.5-4× older than eukaryotes, sequence trees should place eukaryotes clearly relatively shallowly within one or other of the two archaebacterial subphyla, which they never do. By far the most parsimonious interpretation of the overall evidence concerning the topology and root of the tree of life is that the common ancestor of neomura was neither an archaebacterium nor a eubacterium (to label it as either is seriously confusing), but a transitional intermediate between these two major groups, which had eubacterial/eukaryotic type lipids, but neomuran N-linked surface glycoproteins and DNA-handling enzymes [3,4,12].

As this transitional 'missing link' arose somewhat earlier than eukaryotes themselves, during what I named the 'neomuran revolution', the reader is referred to earlier discussions of this enabling revolution in cell structure, and of the compelling evidence from the fossil record and transition analysis that eubacteria are paraphyletic and ancestral to neomura and very much older [6,13]. These papers refute the unwarranted, widespread assumption that the root of the tree of life is between neomura and eubacteria (assumed for example by [52], notwithstanding their mistaken assertion that their Fig 1. tree was 'unrooted'). They detail why that is incorrect, and why the root is within photosynthetic gram-negative eubacteria (Negibacteria: Fig. 1, where ancestrally photosynthetic taxa are green or purple) [6,12,13]. The conclusion that the root of the bacterial tree is within Negibacteria has been questioned on the basis of indel distributions [40,42,47-51], but Valas and Bourne [41] have re-examined these indels critically in the light of protein three dimensional structure and show that the supposed contradictions to my thesis cannot be substantiated and that a root of the tree within Negibacteria, specifically beside or within Chlorobacteria, remains the best interpretation for rooting the tree of life.

As eubacteria are the basal, most ancient group of cells from which neomura evolved, far more genes than often supposed were inherited by the ancestral prekaryote vertically from the eubacterial ancestor of neomura and were already present before mitochondria and nuclei evolved. The absence of numerous eukaryotic gene homologues in archaebacteria, and their presence in many eubacteria, is often used to suggest that they were acquired from the enslaved α-proteobacterium or by independent lateral gene transfer (LGT) [17]. However, that conclusion is probably wrong, being based on dubious assumptions: that LGT is easier than multiple gene losses; that the host was an archaebacterium not a prekaryote derived from the neomuran ancestor. The eubacterial ancestor of neomura could not have been a negibacterium with two bounding membranes, but was a posibacterium with a single surface membrane, like neomura; probably a stem actinobacterium, i.e. an early intermediate between Endobacteria and crown Actinobacteria [4,6,12,13]. As such, it was probably extremely gene-rich. Thus archaebacteria are probably secondarily simplified, their ancestor having lost many hundreds of eubacterial genes during its novel adaptation to hyperthermophily after its divergence from the eukaryote ancestor, e.g. the loss of most genes for aerobic metabolism, including the cytochrome P450s that were precursors of ER respiration, which probably was not derived from the proteobacterial symbiont (see [22] for discussion of the likely gene numbers of the putative intermediates and of the relative contributions of the two major gene donors in eukaryogenesis, which corrects some widespread misconceptions). Like mycoplasmas, archaebacteria are highly derived and specialised, not primitive, bacteria. By contrast their large-celled aerobic sister group, the prekaryote lineage became far more genetically complex through the greatest burst of gene (and probably genome) duplication in the history of life. By the time the nucleus began to evolve, members of this lineage had ceased to be bacteria, having already evolved ER and rudimentary endoskeleton [6,12].

### The eozoan root of the eukaryote tree between Euglenozoa and neokaryotes

Just as one must work upwards from bacteria to eukaryotes with a reliable phylogeny rooted in cellular and palaeontological reality, not unfounded speculation, so one must work downwards systematically from the known diversity of eukaryotes to infer the nature of their last common ancestor. Only when both inferences are sound can we hope to explain the transition from prokaryote to eukaryote realistically. Past reasoning has been hampered by the root of the eukaryote tree also often being misplaced. Though not as inherently difficult as rooting the whole tree of life, correcting these errors has not been easy. We can now rule out the earlier idea that any premitochondrial lineage of eukaryotes survives as there is good evidence that all extant lineages have relics of a mitochondrion [53]; thus the root of the tree cannot lie within the secondarily anaerobic excavate phylum Metamonada that includes *Giardia *and *Trichomonas*, as was often supposed in the past, and must lie amongst ancestrally aerobic protozoan phyla. That the double-membrane mitosomes of *Giardia *indeed evolved from mitochondria and did not evolve separately from α-proteobacteria (as some have speculated) is shown by the presence of Tom40 [54], the outer-membrane protein that in all mitochondria of neokaryotes (i.e. all eukaryotes except the early diverging Euglenozoa [9]) mediates protein import into the mitochondrion from the cytosol.

A few years ago evidence suggested that the eukaryotic root lies between two major groups differing radically in cytoskeletal and ciliary organization: unikonts and bikonts [3,7,8]. Unikonts, comprising animals, fungi, Choanozoa, and Amoebozoa, were postulated ancestrally to have had one cilium and centriole and interphase microtubular cytoskeleton in the form of a cone of single microtubules emanating from the centriole, like a half-spindle [3]. Bikonts, comprising the plant and chromist kingdoms plus 10 protozoan phyla that form four clades (alveolates, excavates, Rhizaria, Apusozoa), ancestrally had an asymmetric cortical skeleton of bands of multiple microtubules forming the roots of two dissimilar cilia and centrioles [3]. However, it now seems that that interpretation was mistaken [9]. One argument for this bifurcation being fundamental was based on a misinterpretation of ciliary and centriolar development of the unikont slime mould *Physarum *that suggested that these were fundamentally different in the unikont Amoebozoa from the prevailing pattern in bikonts, but the discovery of an overlooked correction to the earlier interpretation now makes it likely that most, possibly all, eukaryotes share fundamentally the same pattern of cilia transformation and evolved from an early ancestor that had already evolved two centrioles and ciliary and the widespread pattern of ciliary transformation from a younger to an older cilium [55]. Recent evidence that the biciliate gliding Apusozoa are phylogenetically within unikonts (apusomonads at least probably being sisters to opisthokonts [9,56]) makes it likely that the simple cytoskeletal patterns of Amoebozoa and opisthokonts are secondarily derived following the independent loss respectively of the posterior and anterior cilium. A second argument based on a derived fusion gene against the root being within bikonts [8,57] is also now invalidated by the growing evidence that apusomonads, which have that fusion gene [57], belong within unikonts as sisters to opisthokonts [56].

Another favoured position for the position of the root was within the excavates beside the jakobid flagellates because of the primitive nature of their mitochondrial genome [58]. However, stronger arguments stem from the numerous distinctive features of Euglenozoa, which are most simply explained if the root is between Euglenozoa and excavates [9] as shown in Figure 1. Two euglenozoan characters in particular, the absence of mitochondrial import protein Tom40 and of the DNA replication preinitiation 'origin recognition complex' (ORC), both of which are likely to be ancestral characters of the most primitive eukaryotes rather than secondarily derived simplifications [9]. Previously Tom40 and ORC were assumed to have originated in the ancestral eukaryote; I now think it more likely that both arose somewhat later in the common ancestor of all eukaryotes other than eukaryotes (collectively called neokaryotes to contrast them with the very different Euglenozoa [9]). It seems possible that RNA polymerase II transcription factors IIA, F, and H also originated only in the ancestral neokaryote not the first eukaryote, which are absent from trypanosomatid genomes, unless they were lost by Euglenozoa when their cenancestor replaced the original neomuran transcriptional regulation by posttranscriptional gene regulation [59].

**Figure 2:**
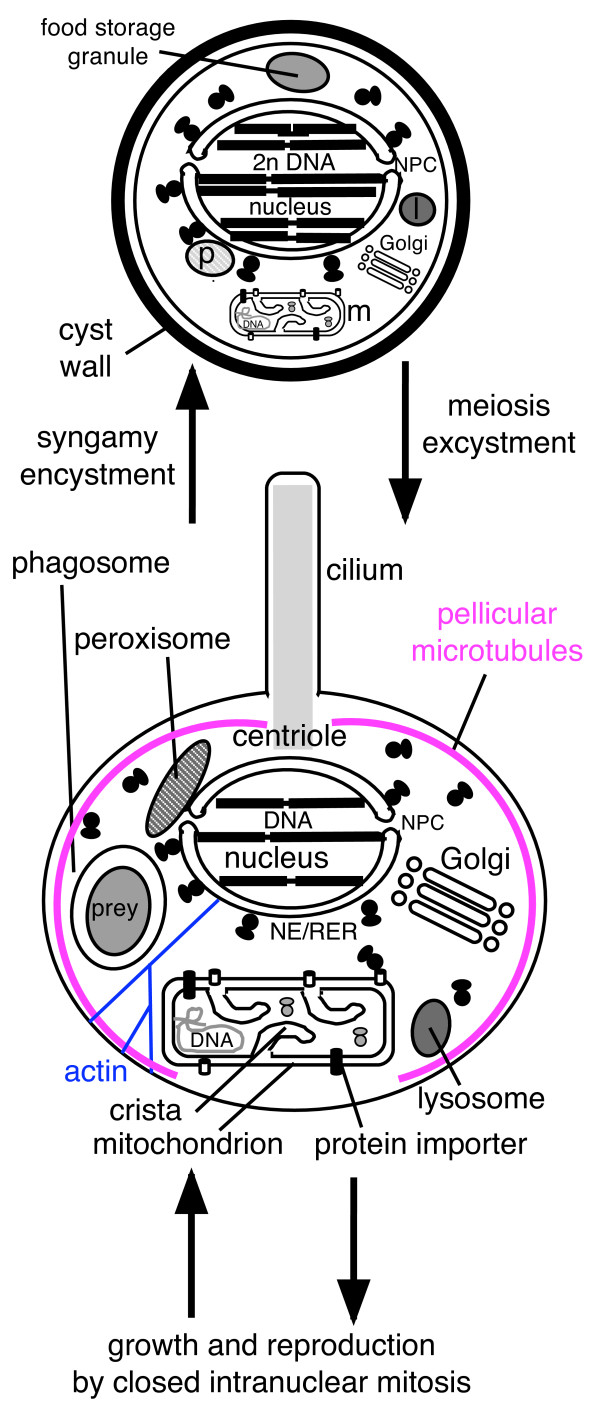
**Inferred life cycle and high degree of organellar complexity of the last common ancestor of all extant eukaryotes**. This reconstruction assumes that the root of the eukaryotic tree is between Euglenozoa and excavates [7,8]. If so, every homologous character present on both sides of the neokaryote/euglenozoan split must have evolved prior to the cenancestor, provided that its later lateral gene transfer from one to the other can be ruled out, as it can for the complex characters shown. The major uncertainty is whether there were only one centriole and cilium as shown or more likely two of each [9]. In addition to the pellicular microtubules there would also have been centriolar roots consisting of bands of microtubules (probably two if the ancestor was uniciliate and three if biciliate) and a specialized anterior cytostome and cytopharynx for prey ingestion (all not shown for simplicity). The peroxisome (p) was probably attached to the nucleus and the Golgi was probably attached to a centrin body; centrin would also have been associated with the centriole and intranuclearly at mitotic spindle poles. The mitochondrion (m) was probably actually attached to the centriole and/or nucleus. A branched actin cytoskeleton permeating the cytoplasm was linked to nuclear envelope (NE) via KASH/Sun integral membrane protein complexes and to the plasma membrane via membrane-embedded integrin proteins. Syngamy involved fusion of plasma membrane, NE, and probably mitochondria.

We can confidently eliminate most other formally possible positions of the eukaryotic root, and infer with high confidence that the last common ancestor of all eukaryotes was a phagotrophic protozoan with nucleus, at least one centriole and cilium, facultatively aerobic mitochondria, sex (meiosis and syngamy) and dormant cyst with cell wall of chitin and/or cellulose, and peroxisomes (Fig. 2); these conclusions all follow whether the root is beside the jakobids or the Euglenozoa. This last ancestor was probably non-photosynthetic, unless cyanobacteria enslaved chloroplasts simultaneously with mitochondria, as has sometimes been proposed [60] but which is unlikely if the root is beside or within Euglenozoa or excavates. Importantly for the present paper, the eukaryotic cenancestor (last common ancestor) already had a highly developed nuclear envelope with complex NPCs with all proteins shared by animals, plants, and Euglenozoa and at least eight different karyopherins to mediate nucleocytoplasmic exchange. Those NPC proteins apparently missing in the parasites *Giardia *and *Plasmodium *[14,17] have either diverged sharply beyond recognition (most likely) or been lost, and do not represent a simpler primitive state as some suggest [17]. This is certainly so for *Plasmodium*, which diverged from plants after the origin of red algae, whose plastid their ancestor enslaved (given chromalveolate monophyly: [61,62]). However, if the root is indeed between Euglenozoa and the rest of eukaryotes, at least some of those apparently absent from the trypanosomatid *Leishmania *[17] might be genuinely, primitively and universally missing from Euglenozoa. The last common ancestor of all eukaryotes (cenancestor) was almost certainly sexual and probably haploid, undergoing syngamy prior to encystment, and meiosis during cyst germination (excystment), but further study of ploidy in early diverging lineages is needed to test this [3,63]. Though most euglenoids have been considered to be asexual (*Scytomonas *is the only one with proven syngamy, but even for it meiosis has not been seen), evidence for relatively normal sexual mechanisms is growing for the euglenozoan *Trypanosoma brucei *[64,65].

This rerooting of the tree is important for thinking about the origin of eukaryotes, as it means that several characters often assumed to be general for eukaryotes and to have evolved in the first eukaryote actually evolved later, e.g. in the ancestor of neokaryotes or neozoa. Thus it now seems that eukaryotic N-linked glycoproteins were probably initially somewhat simpler than in animals and plants. If other Euglenozoa resemble trypanosomatids in lacking glucose termini in the glycosyl group that is added to proteins cotranslationally in the RER [66], then the enzyme making dolichol-phosphate-glucose the donor in neokaryotes for adding three extra residues may have evolved only in the ancestral eozoan, not the first eukaryote; probably quality control over glycoproteins was also simpler as they lack two of the four enzymes that Neozoa use to digest faulty ones (Mannosidase I and peptide-N-glycanase) [67].

Unless the root of the eukaryote tree were within Euglenozoa between the euglenoid *Scytomonas*, which (possibly primitively) has only a single centriole and cilium [68], and all other Euglenozoa that ancestrally had two (a possibility that cannot currently be excluded), then the last common ancestor of eukaryotes almost certainly had two centrioles and cilia per daughter cell. Centrioles would probably have been duplicated at the beginning of S-phase and the two parental centrioles would have separated prior to division, each associated with one new daughter centriole, as in all well studied ciliated eukaryotes. As the closest group to Euglenozoa on unrooted trees is the excavate phylum Percolozoa (Heterolobosea and their relatives) [69], if the root is between Euglenozoa and excavates, the earliest branching excavates would have been the discicristate phylum Percolozoa. This is important for understanding the origin of mitosis, as both Percolozoa and Euglenozoa have intranuclear mitosis with an intact nuclear envelope and a nucleolus that divides, very unlike the open mitosis of animals and plants where the nucleolus and nuclear envelope both disperse prior to metaphase. This probable root position between Euglenozoa and Percolozoa means also that in the ancestral eukaryote (as in both these phyla) the centrioles will not have been be directly at spindle poles but were indirectly attached to them by a cytoplasmic fibrillar cytoskeleton. Moreover, as neither phylum was ancestrally amoeboid, their common ancestor would have had a well developed semi-rigid cell cortex supported by longitudinal cortical microtubules; thus both mitosis and cell division probably evolved in a cell with semi-rigid surface, this rigidity probably stabilising the earliest eukaryotes and allowing fairly accurate DNA segregation following the loss of the eubacterial cell wall; as previously argued [70] the widespread assumption that the earliest eukaryotes were soft-surfaced amoebae is probably a myth; such formless intermediates would have exacerbated the problems of maintaining efficient DNA segregation during eukaryogenesis when the bacterial connection of chromosomal DNA to the cell surface and rigid wall was lost.

Geometric order is essential for DNA and organelle segregation. The important point for this paper is that the eukaryotic cenancestor had at least one centriole and cilium in daughter cells (possibly two) and at least two centrioles (possibly four) in predivision cells, probably attached to the nucleus during interphase to form a karyomastigont complex. Probably the cortical microtubular skeleton that persists during the whole cell cycle and is divided amongst daughters, with new elements being inserted into each, coevolved with the purely temporary mitotic spindle; the origin of the first protozoan pellicle is important for understanding eukaryogenesis as is discussed below. Furthermore, the cenancestral eukaryote had already evolved the coupling of centriole duplication to the onset of DNA replication at the beginning of S-phase [71-76], and had fully eukaryotic cell cycle controls [77] based on cell cycle kinases, phosphatases, and proteases, plus cyclin-mediated anaphase proteolytic resetting of hundred of proteins, as well as growth control over the G1 to S transition and post S-phase involvement of ε-tubulin in centriole duplication [78]. This temporal ordering is as important as geometric order for accurate cell reproduction. All these novelties were evolving simultaneously during the prokaryote-eukaryote transition, effectively at the same time as the nucleus, our main subject.

### Symbiogenesis: an accessory to eukaryogenesis, not the primary instigator

Although symbiogenesis explains the origin of mitochondria, Mereschkowsky's theory of a symbiotic origin of the nucleus [79], and recent attempts to modernize it, are all decisively refuted by the NE being three subdomains of the endoplasmic reticulum (ER) (to say it is 'connected to' ER [17] is wrong; it is ER, invariably having ribosomes on its outer surface); analogies with mitochondria or bacteria are extremely naïve [80]. The really distinctive steps in eukaryogenesis - all much more radical than the helotic origin of mitochondria - were the integrated origins of phagocytosis, endomembranes, endoskeleton, mitosis, nuclei, centrioles, cilia, cell cycle controls, meiosis, and syngamy [3,4,11]. As stressed above, these arguably evolved by the rapid autogenous structural transformation of a bacterial cell through entirely novel selective forces and drastic intracellular structural transformations caused by the onset of phagotrophy [3,4]. Although mitochondrial symbiogenesis involved transfer of many α-proteobacterial genes to the nucleus, whose proteins were often (not always) retargeted to the mitochondrion [20], these genes were probably inessential for any non-mitochondrial major innovations, except for supplying in some transferred genes group II self-splicing introns that evolved into spliceosomal introns and RNAs [3,81]. It is important to realise that the mitochondrion itself is an evolutionary chimaera with many key proteins of host origin being imported, e.g. the inner membrane carrier proteins [20]. Recent trees indicate that the inner membrane proteins Oxa1 (which inserts respiratory chain proteins from the mitochondrial matrix) and Oxa2 (which assembles cytochrome oxidase) evolved in the ancestral eukaryote by duplication from the host's YidC gene, rather than from the α-proteobacterial YidC (as crenarchaeotes lack YidC they cannot have been ancestral to eukaryotes) [82]. On the YidC tree [82] neomuran sequences are a clade (with archaebacteria and eukaryotes sisters) that branches between Endobacteria and Actinobacteria and there is a clear bipartition between the neomuran/posibacterial sequences and those of negibacteria plus chloroplasts, consistently with Fig. 1.

Chloroplast symbiogenesis was probably after the unikont/corticate split (Fig. 1), shortly followed by secondary enslavement of a red alga to yield chromalveolates [83]; both major photosynthetic symbiogeneses replaced host fatty acid and other enzymes, but did not significantly affect basic nuclear properties, except that in chromists among chromalveolates membrane fusion placed the enslaved red alga within the perinuclear cisterna [61]. In contrast to mitochondria the AlbC protein that inserts proteins from the stroma into the thylakoids probably evolved from the cyanobacterial YidC, the host YidC being unavailable by then for such co-option as it had already been modified for mitochondrial function as Oxa1 and 2; its weakly supported failure to group with cyanobacterial rather than other negibacterial sequences is probably artifactual [82]. But even chloroplasts are chimaeric having inner membrane carriers of host origin, and having like mitochondria had their original outer membrane lipopolysaccharides replaced by host phosphatidylcholine.

An important, insufficiently appreciated, feature of symbiogenesis is that it supplied several novel genetic membranes to the eukaryotic cell. In many ways addition of genetic membranes was more important than that of DNA, genes or genomes, because without them genes for oxidative phosphorylation would be useless. Lateral gene transfer had enabled foreign genes to be acquired by bacteria since life began, but for 3.5 Gy never succeeded in transferring oxygenic photosynthesis from one bacterium to another. In free-living prokaryotes cell lineages have been strictly vertical throughout history. By acquiring phagotrophy, eukaryotes could acquire whole cells and novel genetic membranes as well as genomes, not just genes from other organisms, so the inheritance of membranes has rarely been horizontal among unrelated taxa. Of course, sex also involves the horizontal transfer of membranes as well as genes. Membrane heredity is at least as old as DNA heredity - probably older [84] - and just as important for understanding cell evolution [20,62,84-88]. All membranes have been inherited from those of the first cell and the origins of novel mechanisms of protein targeting into and across membranes is central to eukaryogenesis, which involved a marked increase in the number of genetic membranes, some initiated in association with and enabling (not caused by) foreign cell enslavement [85] and some not, i.e. being purely autogenous.

### Coevolutionary origin of the endomembrane system and cytoskeleton

The endomembrane system and cytoskeleton are coadapted and interact in numerous ways. Branching networks of actin attach to plasma membrane, endomembranes and organelles by specific protein links. The endomembrane system fundamentally depends on coat-mediated budding of vesicles from one compartment, uncoating, and fusion of smooth vesicles with target compartments. Both budding and fusion are mediated by suites of mechanical effectors, targeting specificity factors, and controlling proteins among which GTPases play a major role. Vesicles are transported along the cytoskeleton by molecular motors absent in bacteria: myosins for actin filaments, dyneins and kinesins for microtubules. Probably three different functionally specialised myosins were present in the cenancestral eukaryote [7] (this estimate is not changed by recent rerooting the tree [9], though one must now regard the addition of the most widespread tail domains to two of them as neokaryote synapomorphies only, not shared by the earlier diverging Euglenozoa). They evolved during eukaryogenesis by successive gene duplications after their common ancestor became the first myosin by the radical transformation of a former bacterial GTPase by a shift in nucleotide specificity to ATP [89] and various domain fusions [90] to make a complex motor. Likewise the cenancestral eukaryote probably had 11 different heavy chain kinesins [91], with multiple roles in mitosis, ciliary motility and vesicle transport, including interactions with dynein (note that the recent rooting of the tree between Euglenozoa and neokaryotes [9] reduces the previously estimated number in the cenancestral eukaryote because one can now treat kinesins 4-8 and 15 as having evolved later in the ancestral neokaryote only; the rerooting also invalidates the earlier suggestion that kinesin-17 is a synapomorphy for bikonts [91]; instead it probably originated in the ancestral eukaryote and was lost by the ancestral unikont, as it was independently several times within Plantae and Chromista). Kinesins and myosins are structurally related ATPases, probably having a common origin in the prekaryote by a primary gene duplication. Dynein motors are related not to them but to midasin and arose independently from a bacterial ATPase [92]. Duplications produced nine different heavy chain dyneins in the cenancestor [93], most involved in ciliary motility [94].

When I proposed that the origin of actin for a role in phagocytosis was the primary molecular invention that triggered eukaryogenesis [11] it was not even known that actin and myosin were thus involved. Since then a universal role has become apparent for actin and myosin, not only in phagocytosis but in other forms of endocytosis retained even by eukaryotes like yeast, whose ancestors abandoned phagotrophy [95-99]. Thus, an intimate association of actomyosin with endocytosis in all its forms and with endomembrane vesicle trafficking is central for eukaryote cells. Actin is a key player in endomembrane biology and not just involved in general cell motility and cytoplasmic division. It originated from the bacterial membrane skeleton protein MreB, which like actin helps mediate both cell shape and division [100-105]. Prekaryote gene duplications produced not only actin, but actin related proteins (Arps) that nucleate actin filaments, Arp2/3 being essential for branching to make a 3D skeletal network for the first time and for endocytosis. Similarly, six tubulins (α, β for microtubules, γ-tubulin for centrosomal nucleation, and δ, ε, η for centrioles) must have arisen prior to the cenancestor by gene duplications of a relative of FtsZ, the filamentous bacterial GTPase which dates back to the last common ancestor of all life and is the general marker for the site of bacterial division (despite being secondarily lost by the ancestor of many crenarchaeotes and also within Planctobacteria).

### Novel proteins and eukaryogenesis

**Figure 3:**
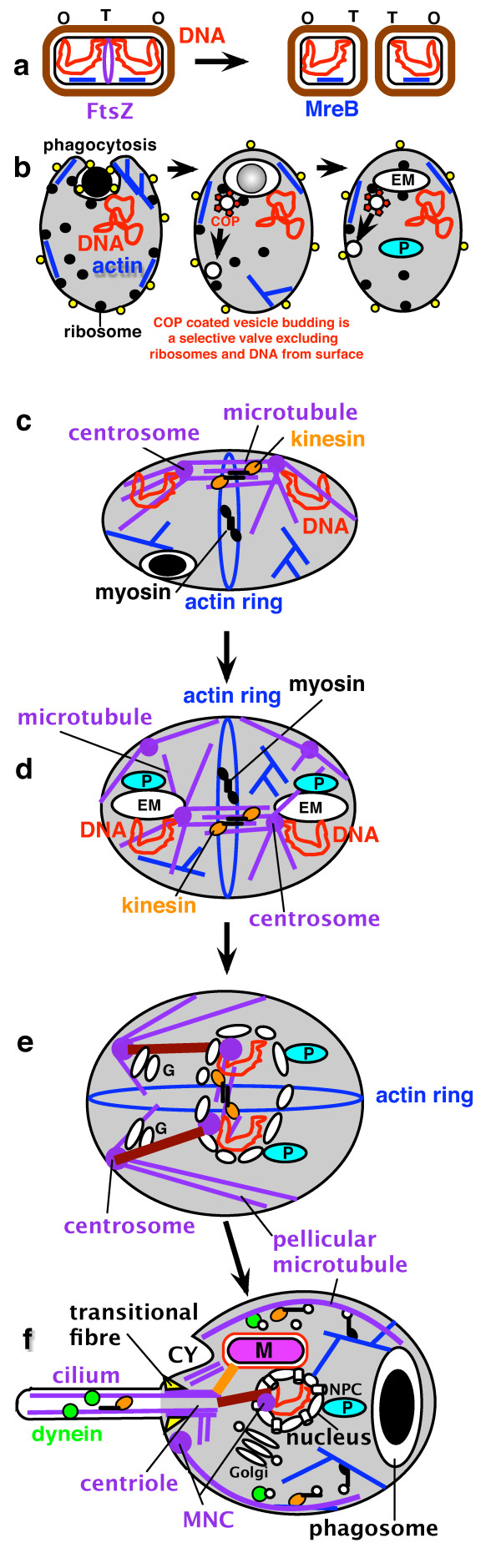
**Evolution of eukaryotes from a posibacterium, emphasizing changes in DNA segregation caused by internalization of DNA-membrane attachments**. (a). An FtsZ ring between daughter DNA termini (T) divides bacteria; cortical skeletal MreB (blue) and rigid murein wall (brown) control cell shape. (b). Disruptive effects of phagotrophy. Left: flexible glycoproteins (yellow) replaced murein, allowing MreB to become actin (blue) and power phagocytosis, which internalised DNA-membrane attachments (centre); evolution of COP-coated vesicle budding, and fusion with plasma membrane after uncoating, made permanent endomembranes (EM: precursor of ER, NE, Golgi, lysosomes; peroxisomes (P) separated earlier) and disrupted bacterial DNA segregation. (c). Hypothetical origin of simple mitosis in a prekaryote where FtsZ gene duplications evolved stable microtubules and γ-tubulin-containing centromeres still attached to the surface membrane. (d). Accidents in centrosome duplication and phagotrophic membrane internalisation generated a more complex prekaryote II in which stable endomembranes differentiated into peroxisomes (P) and protoendomembranes (EM, i.e. the ancestors of ER and Golgi; see [27]), some associated with the internalised centrosome and DNA; another centrosome remained at the cell surface stabilising it; the actin ring controlled the site of cytokinesis. Ultimately the surface centrosome generated pellicular microtubules and centriolar and ciliary microtubules of the cenancestral eukaryote; the ER-associated MNC nucleated its intranuclear spindle. (e). The first eukaryote. (f). Adding NPCs, mitochondria, and cilium, and nuclear chromosome linearization and kinetochore evolution, made the cenancestral eukaryote, shown in G1 of the cell cycle; bacterial ingestion was via a specialised microtubule-supported pocket-like cytostome (CY) at the apical ciliary end, making the cell asymmetric.

Thus prior to the origins of the nucleus and mitochondrion the prekaryote underwent massive gene duplications that created characteristic eukaryote structures [106]; notably of small GTPases involved in vesicle budding or fusion [107]; of actin, tubulins, and molecular motors; of proteins of the proteasome making this digestive cylinder immensely more complex than in bacteria because of the novel ubiquitin-linked proteolytic controls over the cell cycle and removal of faulty ER lumen proteins by the ERAD system [108]; and - of special significance for the origin of the nucleus - coated vesicle proteins (e.g. COPII, COPI, clathrin). These gene duplications, and origins of novel protein domains (the most extensive since cells began) [6], had a key role in eukaryogenesis, the central logic of which is summarized in Fig. 3 in six major stages. (a). Ancestrally an FtsZ ring between daughter DNA termini (T) divided bacteria, their shape being controlled by cortical skeletal MreB (blue) and rigid murein wall (brown). (b). The origin of phagotrophy then disrupted this. In the neomuran ancestor flexible glycoproteins (yellow) replaced murein, allowing MreB in the ancestor of eukaryotes to become actin (blue) and power phagocytosis, which internalised DNA-membrane attachments (centre); soon thereafter evolution of COP-coated vesicle budding, and fusion with plasma membrane after uncoating, made permanent endomembranes (EM: precursor of ER, NE, Golgi, lysosomes; peroxisomes (P) separated earlier) and disrupted bacterial DNA segregation. (c). Hypothetical origin of simple mitosis in a prekaryote cell where FtsZ gene duplications evolved stable microtubules and γ-tubulin-containing centromeres (probably also containing centrin) still attached to the surface membrane. Centrosome binding to the chromosome origin and microtubule attachment parallel to the cell membrane throughout the cell cycle prevented phagocytosis in that region (upper) and thus avoided chromosome internalization as in b but allowed it elsewhere (e.g. lower example shown). Actin and myosin that originally evolved for phagocytosis were recruited to form a contractile ring for cytokinesis, replacing the lost function of FtsZ. Probably they were thus recruited before FtsZ duplicated to α-, β- and γ-tubulin and the prime function of microtubules was to prevent actomyosin dividing the cell before replication finished or internalising the chromosome attachment sites. This stabilising function would be less demanding for the initial origin of microtubules and centrosomes than many more complex later ones. Kinesin (a myosin relative) may have evolved by gene duplication and divergence from myosin at this stage to push apart the antiparallel microtubules attached to the daughter centrosomes, only then would slight microtubule shortening allow the actomyosin ring to cleave the cell. This phagotrophic prekaryote I, with both kinesin and myosin motors that could segregate its DNA and divide the cell, had a centrosome duplication cycle, but no nuclear envelope and perhaps not yet even permanent endomembranes. (d). Accidents in centrosome duplication and phagotrophic membrane internalisation generated a more complex prekaryote II in which stable endomembranes differentiated into peroxisomes (P) and protoendomembranes (EM, i.e. the ancestors of ER and Golgi; see [27]), some of the latter remaining associated with the internalised centrosome and DNA, whereas another centrosome remained at the cell surface and continued to stabilise it and control the site of cytokinesis by the actin ring. Ultimately the surface centrosome generated the pellicular microtubules and the centriolar and ciliary microtubules of the cenancestral eukaryote, whereas the ER-associated MNC nucleated its intranuclear spindle. (e). The first eukaryote. EM attachments around DNA made a primitive NE, and connectors (brown, possibly containing centrin) evolved to ensure that both nuclear and cytoplasmic organelles were properly segregated at division, which was constrained to be between sister cytoplasmic centrosomes and their attached pellicular microtubules. The Golgi apparatus (G) was probably by then differentiated from the ER and attached to the centrosome.

At some stage between (e) and (f) dynein motors evolved to move cargo along microtubules to their minus end (i.e. towards MNCs where γ-tubulin resides). Perhaps initially for moving cytoplasmic vesicles along microtubules (tiny open circles in f, moved by kinesin in the other direction and by myosin along actin filaments), dynein was recruited for sliding ciliary microtubules and (perhaps almost simultaneously) to drive kinetochores on spindle microtubules towards the poles, probably improving segregation, but the origins of kinetochore motility is not discussed here as much of what is known about neokaryote centromeres and kinetochores, mainly based on opisthokonts only [109], may not apply to Euglenozoa or the cenancestral eukaryote. Logically it had to follow the prior evolution of kinesin-driven centrosome separation. Euglenoids, unlike other Euglenozoa, do not exhibit significant anaphase shortening of kinetochore spindle fibres, segregation being largely by centrosome separation and without a prometaphase chromosome movement to form a metaphase equatorial arrangement [110]; the possibility exists that both were also true for the ancestral eukaryote mitosis. Adding NPCs, mitochondria, and cilium (whose basal centriole [=basal body] differentiated following further duplication of the cytoplasmic centrosome and of extra tubulins), and nuclear chromosome linearization and kinetochore evolution, made the cenancestral eukaryote, shown in Fig. 3f in G1 of the cell cycle; a transient intranuclear spindle will develop after the intranuclear centrosome duplicates. The pellicular microtubules prevented phagocytosis over most of the cell surface, so bacterial ingestion was via a specialised microtubule-supported pocket-like cytostome (CY) at the apical ciliary end, which made the cell asymmetric. The mitochondrion was probably attached to the centriole as in kinetoplastid Euglenozoa (orange linker on Fig. 3f), the peroxisome to the nucleus, the Golgi to a centrin-containing body that positioned it near the nucleus and centriole, and the centriole to the cell membrane by the transitional fibres and to many internal structures via two bands of root microtubules (a second centriole and cilium with another microtubular root is not shown in Fig. 3f but would also have been present in the cenancestor unless the eukaryotic root is between *Scytomonas *and other euglenoids [see 9 for discussion of the latter possibility]).

Coated vesicles were crucial: origin of COPII-coated vesicles budding from the primitive endomembranes physically generated from the surface membrane by phagocytosis helped make endomembranes permanent [3]. By fusing with the cell surface after uncoating the first coated vesicles allowed it to grow despite selectively excluding ribosome receptors and DNA-attachment proteins, which caused secretory ribosomes and DNA to remain on the endomembranes, thus creating proto-rough ER/NE [3]. I shall not enumerate all the vesicle coat proteins, SNARE targeting/fusion factors and small GTPases that also must have arisen at this time by gene duplication from bacterial ancestors to generate novel superfamily after superfamily of specifically eukaryotic proteins. Jékely [111] critically reviewed ideas about endomembrane origins and concluded that the autogenous explanation is probably correct and symbiotic models are highly implausible and of little or no explanatory value. I will simply emphasise that this was the largest burst of gene duplication in the history of life, affecting almost every feature of the cell except basic metabolism and bioenergetics (Fig. 3), and now clarify the general character of this innovation and its causes.

### Functional shifts, quantum evolution, and the origins of molecular novelty

Molecules like FtsZ, MreB and the ATPase and GTPase ancestors of eukaryotic motors originated in the first bacteria about 3.5 billion years ago. Ever since they have been gradually diverging and changing in small ways that do not radically affect their function, which is essentially the same in extant bacteria as in their 3.5 billion year old ancestors. Likewise their very different descendant tubulins, actin, and dynein, myosin, and kinesin motors have been evolving slowly and mutually diverging for over 800 million years without radical change. What has kept their change so slow, within bacteria and within eukaryotes, that we can make comprehensible sequence trees to trace their divergence of over such long periods is very strong stabilising or purifying selection; this eliminates variants that too much disrupt their function and interactions with the hundreds of other cellular components [112]. Thus for most of earth history stabilising selection limiting change, and minor directional changes perfecting the details and adapting the descendants to slightly different niches, have been the dominant force. In marked contrast, during the origin of eukaryotes all these molecules radically altered in that one lineage only. If one were to extrapolate the slow rate of change normally seen in such molecules to the prokaryote-eukaryote transition it would take several times the age of the universe to effect as much change as actually happened in a time so short geologically as to be a mere blink of an eyelid. No other lineages ever changed in this radical way; only one did. It may have done so, I contend, in much less than 0.1 million years. The changes undergone in this one lineage by these proteins were many orders of magnitude faster and more extensive than the generally slow changes in the bacterial and eukaryotic versions of these molecules. Such extremely rapid evolution is what the great palaeontologist and evolutionist Simpson called quantum evolution [10,113], because it occurs almost instantaneously from the long perspective of the geological timescale. He pointed out that during the origin of a new body plan some features of an organism - those involved directly in the greatest novelty - invariably undergo such extreme quantum evolution, whereas others are extremely conservative and hardly change at all [10,113].

Thus evolution of new body plans is characteristically mosaic [114], affecting some key characters immensely more than others. Some undergo quantum evolution; some are almost static. In sequence terms, such mosaic evolution (not to be confused with chimaeric evolution) means that molecules that are unaffected by the innovation may continue to evolve at their normal slow, relatively steady (though not strictly clock-like) pace throughout a major transition yielding a new body plan, e.g. many metabolic enzymes of unaltered function. At the other extreme some evolve so rapidly that new protein domains are invented, even erasing evidence of past relationships [12,22]. Somewhat less dramatically changed proteins retain structural evidence of their ancestry but their sequences diverge so much that one cannot make sequence trees that include both ancestral and descendant molecules, e.g. the molecular motors. Major innovation often also involves both gene duplications and gene fusions to make more complex chimaeric genes with multiple domains. This non-uniform broad spectrum of evolutionary modes during a major transition has important implications for our ability to reconstruct the changes and for what methods are most appropriate. Thus, molecules that have undergone the most dramatic change are simultaneously the most important for understanding its basic nature, e.g. the nuclear pore complex and lamina structural proteins for nuclear origins, but also the most difficult to use for sequence trees and sequence-based bioinformatic methods. By contrast those to which such methods can be best applied are the most conservative molecules of unchanged function that only underwent trivial changes and are essentially irrelevant for understanding the change itself, and of use only as phylogenetic markers for tracing the origin of a different minor subset of cell components. In the middle of the spectrum are conservative molecules which were not central to the change, and therefore retained their ancestral function and enough useful information for us to make trees, yet nonetheless were sufficiently strongly affected indirectly by the changes as to have undergone a temporary major acceleration in evolutionary rate and coadaptive evolution during the transition.

Among such intermediate-character molecules in the case of eukaryogenesis are ribosomal RNA and proteins and the RNA polymerases. Their basic function was unchanged during the origin of eukaryotes, but the functions of RNA polymerases were so significantly modified by the duplications that generated separate polymerases for rRNA, mRNA and tRNA that they underwent a rapid spurt of evolution as they became specialised for these subtly different roles; once thus perfected they thereafter settled down to the normal slower paced evolution dominated by purifying selection [3]. Such a rapid and substantial, but purely temporary, spurt of evolution is the general rule for the evolution of paralogues by gene duplication [13]. The more radical the modification the longer will be the stem at the base of each paralogue subtree. But the length of these sister stems is related almost entirely to the degree of functional shift during their primary divergence, bearing little or no relation to elapsed time; commonly for novel eukaryote-specific paralogues it is as long as the whole subsequent slow diversification phase; the latter lasted at least 800 million years, but to assume that the contrasting adaptation of two sister paralogues would also take 800 million years before any eukaryotes could diversify and leave modern descendants is evolutionarily ridiculous.

Population genetics of large microbial populations and modest selective forces could achieve this in much less than 80,000 years, even 8,000 years (well over 3 million generations; ten times the number that separates us from our common ancestor with other apes), so very likely stem lengths of paralogue subtrees are commonly inflated 10,000-fold or more by quantum evolutionary divergence consequential upon divergence of sister paralogues, compared with rates of change deduced by comparisons among derived groups where purifying selection dominates and keeps change slow. As argued previously [12], a comparable gross rate inflation probably applied to cytoplasmic rRNA temporarily during eukaryogenesis as a consequence of the evolution of the nuclear envelope and nucleolus with novel processing and transport (but **not **to mitochondrial rRNA, which evaded these novel coevolutionary selective forces by being kept entirely within the mitochondrial matrix). Thus, extreme quantum evolution can occur not only following divergence of paralogues but in unduplicated molecules given a sufficiently intense shift of function. Overlooking the fundamentally different evolutionary principles that apply to megaevolution compared with ordinary macroevolutionary divergence has led to much misinterpretation of rRNA and paralogue trees. These misinterpretations have most seriously affected interpretations of the timing of evolutionary events and the rooting of trees, but quantum evolution can also introduce such serious long-branch artefacts that no algorithm can reconstruct the correct topology. Molecules like rRNA and RNA polymerase are not evolutionary chronometers, but evolve extremely heterogeneously in speed across time; rates can be roughly estimated only by considering both the fossil record and the degree to which quantum evolution may be involved, which cannot be statistically modelled - unique events like the origin of eukaryotes or the vertebrate skeleton violate all naïve statistical assumptions of regularity and repetitiveness. It is fundamentally wrong to assume that all molecules evolve the same way throughout time and to ignore unpredictable irregularities. A related kind of misinterpretation concerns the source of the different components of eukaryotes. For example Esser et al. [115] concluded that among bioinformatically tractable proteins, the majority in yeast were more similar to those of eubacteria than to archaebacteria. From this truth they concluded a probable falsehood - that most nuclear genes came from the α-proteobacterial ancestor of mitochondria.

There are three reasons why that conclusion is unsound. First, the evidence that eubacteria are ancestral to archaebacteria implies that ancestral archaebacteria probably lost about 1000 genes compared with eubacteria [6,13,22]. If most of those genes were lost after they diverged from the prekaryote lineage, 1000 extra genes of host origin would have been present in the host to leave modern eukaryotic descendants, which would radically increase the proportion deduced to have come from the host, by itself completely reversing the authors' conclusion. In other words, the genes now in archaebacteria grossly underestimate the much wider spectrum present in the neomuran ancestor; many eubacteria-like eukaryotic genes probably came not from the mitochondrion (as others undoubtedly did) but from the host, their sister homologues being lost by the archaebacteria. Secondly, proteins well enough conserved during the prokaryote/eukaryote transition to allow such sequence comparisons are a small, non-representative sample of the total. Most of those host proteins that evolved radically novel, specifically eukaryotic, functions (such as nuclear pore complexes and vesicle coat proteins, and molecular motors) were so transformed by quantum evolution that their sequences are too divergent to be recognisable by BLAST analysis. Evidence of the precise phylogenetic origin of the majority of eukaryotic genes has simply been erased by the quantum evolution that made eukaryotes. Probably the vast majority came vertically from the host, not the α-proteobacterial symbiont. The third major bias to the proportions is symbiont replacement of functionally equivalent host genes. This well-known evolutionary phenomenon applies not only to the primary symbiogenetic origin of mitochondria and chloroplasts, but to secondary symbiogenesis, e.g. the acquisition of plant fatty acid biosynthesis genes from the enslaved cyanobacterium [83,116] and later from the enslaved red alga by the ancestral chromalveolate [117]. Symbiogenesis introduces thousands of symbiont genes into the host nucleus. If the encoded proteins were exactly equivalent to host proteins, there is a good chance that some will replace host proteins, since among two equally well expressed and well controlled proteins it does not matter which is lost by functionally advantageous elimination of wasteful duplicates [60]. If the symbiont is a bacterium, as was the protomitochondrion, the host versions of many of the relatively unaltered metabolic genes they share will be eliminated. But novel host eukaryotic genes that have become so different from their bacterial progenitors that the protomitochondrion had no close relatives are less likely to be lost. Thus, host gene replacement by symbiont genes is biased towards a small subset of genes - relatively conserved and bioinformatically tractable metabolic genes and against those very genes that played the most important role in the origin of eukaryotes and whose ancestry cannot be traced from their sequence.

The value of symbiogenesis was that it created a novel organelle with minimal structural change to the enslaved bacterium and no fundamental genetic change simply by adding one novelty: inner membrane carriers and associated protein-import machinery [20,22]; the elimination of thousands of symbiont genes and retargeting of some were inevitable consequences of this one key innovation. Symbiogenesis probably contributed very little, if anything, to the key initial steps in eukaryogenesis, the concerted origin of the cytoskeleton, endomembranes, and mitosis. Two major effects on the host were the origin of spliceosomal introns from α-proteobacterial group II self splicing introns, and the loss of its own plasma membrane/endomembrane ATP-generating, oxidative phosphorylation enzymes - assuming that the host, like the symbiont was a facultative aerobe able to live both in fully aerobic and temporarily anaerobic environment [22].

## Results: the Origin of Mitosis and the Nucleus

### Megaevolutionary principles apply to cell and molecular evolution

From the classic megaevolutionary perspective of Simpson [10,113], understanding the origin of eukaryotes (eukaryogenesis) is much less difficult than is often supposed, despite it being the most dramatic molecular, cellular and genetic upheaval in the history of life. The central requirement for a convincing explanation is to specify correctly the adaptive shift that caused the innovation in body plan and the nature of the ancestral organism that made that innovation - in this case predation by phagotrophy. Thereafter it is relatively straightforward to reconstruct a biologically sensible and mechanistically plausible sequence of likely successive changes.

Most megaevolution occurs without any symbiogenesis; only five megaevolutionary events in the history of life involved symbiogenesis. In the origin of the kingdoms Plantae and Chromista (now = chromalveolates [9]), of chlorarachnean Cercozoa and Euglenia, the internal symbiogenetic enslavement of a photosynthetic cell was the central determinative event; in every case phagocytosis engulfed the slave, proving that phagotrophy preadapts a cell for symbiogenetic enslavement of other cells. Symbiogenesis was of the essence, but phagotrophy was the essential preadaptation. In the fifth case, the origin of eukaryotes, phagotrophy was causally primary and mitochondrial enslavement purely secondary. The fundamental novelty in body plan and way of life of eukaryotes involved the unique origin of phagotrophy, internal cytoskeleton and endomembranes. Very likely the host that acquired a mitochondrion was already a facultative aerobe with oxidative phosphorylation [3]. Adding mitochondria was an important extra refinement to increase energy efficiency, but probably did not fundamentally change the cell's way of life or body plan or impose any radically new selective forces on it as did the evolution of phagocytic prey capture and internal digestion. Oxidative phosphorylation had been around for ~2 billion years before eukaryotes evolved [12]; phagotrophy only arose with them. Margulis [32,33] claimed that mitochondrial enslavement preceded and enabled phagotrophy. But neither she nor recent adherents to this unsubstantiated view (e.g. [26,36]) ever convincingly explained how such an implausible reversal of the above logic could have worked selectively or mechanistically to produce the cytoskeleton and endomembrane system.

Simpson emphasised that the magnitude of megaevolutionary steps ensure that they occur only if the ancestral lineage was in many ways preadapted for the transition, both by its structure and physiology and its ecological accessibility to the empty adaptive zone that it alone has a realistic potential to invade. Evolution occurs not in abstract sequence space, but in the real world of organisms with specific body plans and ecological contexts. Preadaptation does not imply evolutionary foresight or planning; it is just an historical accident - not a lethal accident or uncaused accident, but one culminating a unique stream of historical events that gave one lineage uniquely suitable properties for being a viable vehicle for mutations of radical effects that would have extinguished less fitted lineages of different background. Fish with lungs and lobed fins happened to be preadapted for land life and becoming tetrapods, in a way that fish with spiny fins and no lungs, or still worse echinoderms or jellyfish, which never made it to land, were not. Phylogenetic preadaptation of a unique lineage is a necessary part of the explanation of every megaevolutionary event. Exceptional preadaptation and discontinuity invariably apply to body plan innovation. Thus, the origin of eukaryotes was necessarily an intrinsically abnormal event, not one understandable simply by extrapolating the trivial tinkering that occupies most evolution. To understand it we must invoke something quite exceptional. Actually four things, none miraculous but all uniquely innovative: one is preadaptation of one lineage for phagotrophy; secondly the novel selective forces that phagotrophy brings; thirdly the disruptive effects that phagotrophy had on the cell division and DNA segregation machinery by internalising the DNA-membrane attachment sites [4,11]. Not only did this disruption necessitate the adoption of novel systems (cell division by actomyosin and DNA segregation by microtubules/kinesin), but also because these were inevitably initially inefficient it directly caused repeated polyploidy (whole genome duplication in modern jargon) in the transitional intermediate. This would make the cells large, as in the giant bacterium *Epulopiscium *[118], probably advantageous for ingesting others as food. Every gene probably duplicated many times in just a few days. The intermediate was almost certainly multigenomic and in consequence also larger than its ancestors and better able to engulf other cells and also survive some otherwise harmful mutations. Interestingly, large complex filamentous multigenomic cyanobacteria and actinomycetes can segregate their DNA randomly, and thus remain viable without MreB, unlike most bacteria [58,119]. Advanced large, metabolically diversified and multigenomic prokaryotes, not the tiny degenerate ones implausibly postulated by Margulis [32,33,120] as our ancestors, could best have made the transition to eukaryotes.

### Key novelties and preadaptations for eukaryogenesis

There were three key preadaptive features of the ancestor of eukaryotes. First it had one bounding membrane (like Posibacteria and Archaebacteria alone among prokaryotes), not two as in all other eight eubacterial phyla, which would have prevented the evolution of phagocytosis and endomembrane budding. Thus of eubacteria, only Posibacteria were preadapted to become the ancestor of either eukaryotes or archaebacteria. This first preadaptation was the origin of Posibacteria by the loss of the negibacterial outer membrane, probably at least 700 million years before the origin of eukaryotes, thus not a triggering event [121]. Secondly, the three dimensionally covalently bonded murein wall of most eubacteria makes both the origin of phagotrophy and sex (cell fusion and ploidy reduction by meiosis) impossible. The essential preadaptation was the replacement of the murein cell wall by individually potentially mobile surface N-linked glycoproteins. This neomuran revolution was I argued the triggering event for the origin of eukaryotes [4] and equally of their archaebacterial sisters; each evolved divergently, one by genome reduction and cell compaction and the other by gene multiplication and cellular expansion. The third preadaptation for eukaryogenesis was a combination of ideal cellular factors: large cell size to enable engulfing of other bacteria, large genome size with many potential gene ancestors, a diverse set of lipids to favour endomembrane differentiation, a large secretome including many digestive enzymes as precursors for internal digestion (about a third of eukaryotic proteins are secretory); and a facultatively aerobic/anaerobic metabolism to allow them most simply to become hosts to a facultative aerobic/anaerobic, probably photosynthetic, α-proteobacterium [3,4].

Of the two posibacterial subphyla, Endobacteria and Actinobacteria, no Endobacteria were thus preadapted, but many actinobacteria were. Actinobacteria have the largest secretome of any bacteria - over 800 secretory proteins, mostly digestive enzymes, large genomes and cells, complex life cycles with protein phosphorylation controls, differentiated resting spores, phosphatidylinositol, proteasomes of the neomuran type for intracellular protein digestion, and H1-like histone, and enzymes related to those that make eukaryotic sphingolipids, and many more cytochrome P450 (precursors of the ER oxidation system) than other bacteria. Regulation of actin polymerisation by phosphatidylinositol 4,5-bisphosphate activated by a small GTPase [107] can only have evolved in an actinobacterium-derived ancestor, as no other prokaryotes whatsoever have phosphatidylinositol [13]; the inositol phospholipids of myxobacteria are chemically different (dialkyl ethers not acyl esters; see [13]); citing them as evidence for a myxobacterial rather than an actinobacterial ancestry [122] is a spurious argument. Duplications of the same GTPase gene family provided Ran GTPase for NE assembly and nucleocytoplasmic transport and GTPases for endomembrane differentiation during the multiplication of novel genetic membranes [85,107]. Among actinobacteria, actinomycetes are of special significance for eukaryogenesis (and the origin of archaebacteria) as they not only have eukaryote-like small GTPases, crucial for endomembrane differentiation and nuclear origins, and a neomuran type proteasome, but also biosynthetic pathways for polyketides like fungi, protozoa, chromalveolates and plants; mycobacteria can also make cholesterol. Contrary to Margulis [32], who suggested that mitochondrial enslavement by a mycoplasma-like host introduced sterol synthesis into prekaryotes thereby enabling phagotrophy, α-proteobacteria do not make sterols, and mycoplasmas are too small to have been the host; they evolved as intracellular parasites inside eukaryote cells after phagocytosis had already evolved, enabling a posibacterial ancestor to lose its murein wall in an osmotically protective environment and for mycoplasma cells and genomes to be miniaturized. Mycoplasmas lost their posibacterial walls independently of the neomuran ancestor. The genomic reduction of the ancestral archaebacterium, associated small size, lack of extensive secretome or suitable lipid precursors, more anaerobic lifestyle, a likely hyperthermophilic specialisation, absence of phosphatidylinositol or other phospholipids and sterols, all make archaebacteria entirely implausible as ancestors of the eukaryote host, being devoid of most preadaptations that would have been present in the neomuran ancestor. Though in lipid biology mycobacteria seem preadapted for the evolution of eukaryotes, they are so specialised that the actual ancestor of neomura was more likely a less specialised early diverging relative with similar metabolic capabilities.

**Figure 4:**
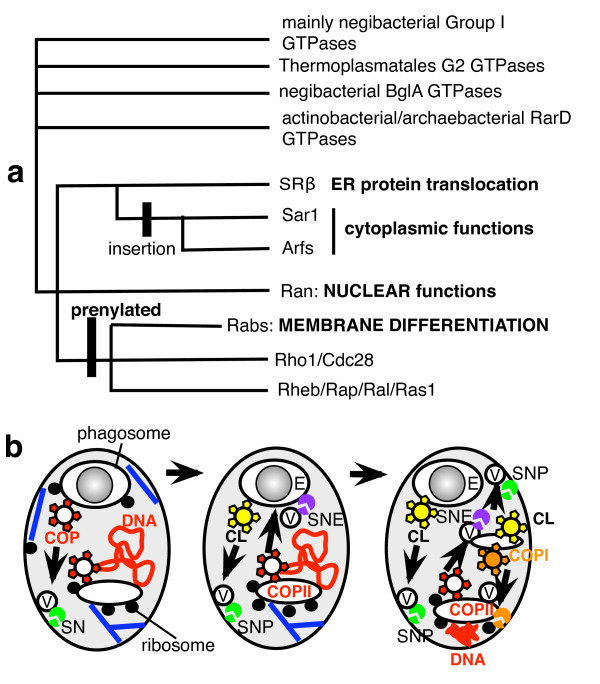
**Evolutionary differentiation of endomembranes**. (a). Schematic tree for controlling small GTPases [124,128]. Sar-1 and Arf-1 have an extra, derived insertion, so the root cannot be in that branch. Because of disparate rates of evolution among paralogues and the shortness of the molecules it is unclear from trees whether the seven eukaryotic clades (lower) are all mutually related as shown and which of the four bacterial clades (upper) are their closest relatives. (b). After endomembranes, peroxisomes, and plasma membrane became distinct genetic membranes (Fig. 3b) most secretory ribosomes were on old DNA-bearing cisternae; the first COP/adaptin coats generated vesicles (V) from the protoendomembrane/phagosome; early SNAREs (SN, left) fused them with the plasma membrane. Endomembrane differentiation improved digestion by targeting digestive enzymes specifically to phagosomes, mediated by successive concerted duplications and divergence of coat proteins, cognate SNAREs able to bind to them, and associated small GTPases. Primary specialisation between digestion and synthesis involved clathrin vesicles (CL) associated with plasma membrane SNAREs (SNP) and COPII vesicles associated with endomembrane SNAREs (SNE). Interpolation of Golgi, by mutual fusion of uncoated COPII vesicles, stabilised by COPI-mediated recycling (right), allowed specialisation between lysosomes and surface growth. For a fuller discussion of endomembrane origins see [91].

Jékely [107] suggested that eukaryogenesis was initiated not by phagocytosis, but by the evolution of secretion by exocytosis, with phagocytosis arising later, simply because this appeared more compatible with his small GTPase sequence tree. While I once briefly held that view [70,123], it has immensely less explanatory power than phagotrophy as the initiator [3,4,6]. Moreover, the GTPase tree that fuelled his proposal (see Fig. 4a) was misleading through concealing branch lengths and misinterpreted. It is evident from other work that the Arf branch is the longest among the eukaryote paralogues and that the small GTPase tree is essentially unresolved at its base [124]; therefore rooting of the tree with the far longer eubacterial outgroup could have wrongly attracted it to the base. It is as unwise as for other paralogue trees to assume that the rooting is accurate. Even if the position of the root is correct, it was wrong to infer that the Arf containing branch arose prior to the Rab-containing branch. In such a basal divergence one cannot say that one sister branch is earlier than the other. The worst argument, however, was that the Arf-1 branch is involved in secretion and the Rab one in phagocytosis, and to combine these two bad arguments to conclude that exocytosis evolved before phagocytosis [107]. In fact, there is ample evidence that Rab paralogues are extensively involved in secretion in early, middle and late stages [125,126], and conversely that Arf1 is involved on phagocytosis [127]. Thus Arf and the Rab paralogues are both required for secretion and exocytosis and equally for endocytosis and phagocytosis, decisively invalidating any attempt to infer the relative timing of the origin of these complementary processes from GTPase paralogue trees. Since his work additional prokaryotic small GTPases paralogue classes have been discovered, which complicate the story and disrupt the branching pattern of the tree [128]. In my view these authors also are overconfident of their (somewhat different) tree and misinterpret it as evidence for separate origins of Arf/Sar and Rab/Ras/Rho from different bacteria; more likely both these major classes of eukaryotic GTPases have a common ancestry in the RarD paralogue found only in archaebacteria and actinobacteria [87]. Rather than making dubious deductions from possibly distorted sequence trees, it is preferable to use general evolutionary and cell biological principles and as much relevant knowledge as possible to infer the overall most likely sequence of events. Fig. 4b therefore proposes a plausible and simple scenario for endomembrane differentiation prior to the origin of the nucleus; almost certainly endocytosis and exocytosis coevolved, recruiting from a common pool of enzymes when assembling their toolkit.

Endomembrane differentiation required not only duplications of vesicle coat proteins and GTPases controlling budding but also of SNAREs controlling targeting of each type of uncoated vesicle [85] (and GTPases controlling fusion) [107]. Even the first step, separation of rough ER (RER) from plasma membrane probably needed SNAREs initially. 20 different SNAREs arose before the eukaryotic cenancestor [129]. The genetic identity of RER was secured by insertion of SRP-recognising docking protein (pentagons Fig. 3a, b) in which small GTPase SRβ participates [107]. It was more efficient for digestive enzyme-laded CopII vesicles budding from proto-ER to fuse with phagosomes not the cell surface. Thus was born the primary divergence between ER outwards secretory traffic and plasma membrane inwards endocytic traffic seen in GTPase [107] and SNARE [130] trees. So we should not ask 'did phagocytosis or exocytosis evolve first?' Both evolved together, with phagotrophy being the entirely novel selective advantage, as De Duve [131] and Stanier [2] first argued. I refer the reader to [27] for a more detailed discussion of the origin of the endomembrane system, which had to precede the origin of the nucleus.

### The overlapping origins of mitosis, centrioles and the nucleus

As Fig. 3 indicated, a simple form of mitosis probably originated before the nucleus (Fig. 3c), but the modern version is more complex and its elaboration must have overlapped and coevolved with the origin of the nucleus. A nucleus could not evolve without a relatively efficient means of DNA segregation, making it misleading to consider the origin of the nucleus on its own, as many do. I shall argue that NPCs and nucleocytoplasmic transport and the chromatin-attachment of NPCs and the inner envelope membrane - the key features of the cell nucleus - evolved by the structural and functional integration of novel duplicates of pre-existing key proteins whose basic functions originally evolved separately for endomembrane and chromatin-associated mitotic functions. Before explaining this integration I must outline the origins of endomembranes and of mitosis, the essential prerequisites for the origin of the nuclear envelope, NPCs and nucleocytoplasmic compartmentation.

As explained in the first detailed discussion of the logic of the transition from bacterial to eukaryotic DNA segregation [70], this involves three major changes. First a change in the timing of DNA replication relative to mechanical separation; both processes are spread over almost the whole cell cycle in bacteria and replication origins are separated (as then postulated, but now known [132,133]) well before replication is complete, whereas in eukaryotes replication is completed before sister chromatid separation begins. Second was the origin of the eukaryotic chromosome condensation cycle in which DNA is relatively dispersed for replication and transcription but typically more tightly condensed during mitosis. Third, was a topographic change from chromosomes being attached to and geometrically ordered on the bacterial cell surface membrane and wall to chromosomes being attached to the nuclear envelope in interphase during replication but attached instead to the mitotic spindle microtubules during cell division. Spindle microtubules consist of α- and β-tubulin and are nucleated by γ-tubulin at the core of the centrosome; when these and the other universal tubulins evolved by repeated gene duplications of a bacterial GTPase they underwent such radical changes in a short time that they now have only a slight sequence similarities to their bacterial relatives FtsZ [134] and TubZ [135]. It has usually been assumed that tubulin evolved from FtsZ [136], whose single filaments form the core of the Z-ring which marks the site of division in most bacteria and chloroplasts and the more primitive mitochondria. However, the recently discovered GTPase TubZ, a double filament protein that is important for the segregation of DNA in certain plasmids of the posibacterium *Bacillus*, is a better analogue of tubulin, because it seems to have similar treadmilling and capping properties and might actually be involved in pushing apart sister DNA molecules [135,137], unlike FtsZ which is more a morphogenetic marker to guide the membrane scission machinery into place. The fact that TubZ coexists in cells with FtsZ means that its DNA segregation properties can evolve without at the same time abandoning FtsZ's marker function. I suggest that tubulins evolved from either an early relative of TubZ itself (quite likely as it evolved like tubulin in the posibacterial lineage) or an independently evolved paralogue of FtsZ that functioned similarly in plasmid DNA segregation. TubZ evolves much faster than either tubulin or FtsZ, presumably because it interacts with fewer other proteins, and is extremely divergent in sequence from both. During the origins of α-, β-tubulin γ-tubulin numerous new features evolved to allow α- and β-tubulin to form dimers and their protofilaments to form microtubules and γ-tubulin to nucleate them, so much so that sequence trees cannot be expected to establish which bacterial paralogue tubulin evolved from [134]. However, an origin from a treadmilling posibacterial TubZ involved in plasmid DNA separation would offer the easiest transition for the origin of microtubules and the mitotic spindle, with the least trauma for the evolving phagotroph.

By contrast there was a much less radical change in the organization of a fourth key component of the DNA segregation system, whose role and universal importance to DNA segregation was unknown even 20 years ago: SMC proteins and kleisins [138]. SMC (acronym of structural maintenance of chromosomes) proteins have homologues in all organisms and are essential for accurate DNA segregation. SMCs are giant proteins with a globular domain at each end and a long helically coiled rod in the middle. They exist as dimers in which the rods are antiparallel and twisted around each other as coiled coils, and hinged in the middle enabling them to form a V shape in which the end of each arm contains one C-terminal and one N-terminal globular domain. This close association of the contrasting domains enables the globular head end of each arm to bind ATP and function as an ATPase. Each head interacts with kleisin proteins; when these are bound to both, the whole complex forms a ring known as a cohesin or condensin (depending on its function and which SMCs and kleisins it contains). Cohesin rings enclose sister DNA molecules and prevent their separation till the cell is ready to divide, because they are loaded onto the parent DNA molecule before replication is completed (in eukaryotes before it even starts). When the cell is ready to divide proteases digest the kleisin (at anaphase in eukaryotes), converting the ring into an open V, thereby allowing the mechanical separation of DNA sister molecules (chromatids) [139,140].

Eukaryotic cohesins are loaded into DNA at the G1-S phase transition. Bacterial DNA segregation broadly follows the principles adumbrated long ago [141], but we now know the molecular machinery [132,133]. Bacterial SMC condensins are loaded onto replication origins by the DNA partitioning protein ParB and thereby mediate chromosome compaction of daughter chromosomes [132,133,142], which is essential for accurate DNA segregation as the origins are moved to opposite poles by an ATPase (Soj), i.e. actively as postulated [141]. Thus DNA gyrase in eubacteria and histones in eukaryotes are not sufficient for chromosome compaction as was originally assumed [70]. The ring-shaped condensins that hold DNA loops together to allow proper chromosome folding [142] clearly evolved in the last common ancestor of all life, as SMC is known from most bacterial phyla [143] and I have found them by BLAST in the earliest diverging Chlorobacteria (their greater sequence divergence within this phylum compared with most others is consistent with it being the oldest [6,13]). Moreover SMC had also undergone two gene duplications in the last common ancestor to generate two proteins for DNA repair: Rec9 and SbcC. RecN is a eubacterial protein involved in repairing double-strand breaks in DNA; neomura have a homologue (also for break repair) called Rad50 that probably evolved from it. SbcC helps in other forms of DNA repair and seems to have been lost by the ancestral neomuran. A small subset of γ-proteobacteria of the family Enterobacteriacae (including *Escherichia, Salmonella *and *Shigella*) replaced SMC by a larger but related protein, MukB that interacts with different kleisins, and groups on trees with SbcC and must have evolved either from it or from SMC by radical changes. Possibly these unique drastic changes in these enterobacterial SMC-like proteins occurred when their ancestor (alone among prokaryotes?) evolved the capacity to initiate several successive rounds of chromosome replications, yielding branching chromosomes that allow the cell cycle to become even shorter than the replication fork travel time [141].

SMC proteins are so long (over 100 amino acids) and well conserved that they make better sequence trees [143] than most proteins commonly used for prokaryote phylogeny (e.g. [45]); the SMC tree shows archaebacteria, neomura, and 'neomura plus Posibacteria' all as monophyletic and places neomura between actinobacteria and Endobacteria as in Fig. 1 (though as sisters of Endobacteria not Actinobacteria). Prokaryote SMCs are all homodimers, whereas in eukaryotes they are always heterodimers. During the origin of eukaryotes two gene duplications generated four paralogues [143]: Smc1 and 3, which form cohesin by binding the kleisin Ssc1; and Smc2 and 4, which form condensin I by binding the kleisin CAP-H (or CAP-H2 in condensin II which evolved later in an early animal only). As Smc1 and 2 group together on trees, as do Smc3 and 4, it is likely that stem eukaryotes initially evolved an ancestral general purpose heterodimer containing one of each of these pairs, but that further duplications differentiated it into cohesin (loaded onto chromatin in interphase) and condensin (loaded during prophase of mitosis). This differentiation must have gone hand in hand with the new temporal controls via cyclins and phosphorylation. The first heterodimeric SMC was probably a condensin as in bacteria; the newer function of cohesin depended on the coevolution of novel anaphase proteolysis of Scc1 (see below). Though Euglenozoa have condensin I, and cohesin, Ssc1 and the separase enzyme that cuts it are essential for chromosome segregation [144], they lack another Smc heterodimer (Smc5/6), which may act as a cohesin for rDNA segregation [144] as well as having functions in DNA repair or recombination, suggesting that it evolved somewhat later (in the ancestral neokaryote, being present in *Naegleria *and *Giardia *genomes).

Chromosome condensation, separation and all other aspects of mitosis and the cell cycle are regulated by four related families of eukaryotic serine/threonine (S/T) protein kinases: Cdk, Nek, aurora and polo-like. All are present in the most divergent eukaryotes (Euglenozoa) so all originated by gene duplication in the ancestral eukaryote. Their closest relatives and putative ancestors of these and numerous other eukaryotic families of S/T kinases is the PKN2 family of eubacterial kinases which are widely and mainly present in Posibacteria (both subphyla) and Cyanobacteria but never in archaebacteria [145]. Almost certainly they were vertically inherited from Posibacteria to the ancestral neomuran and immensely multiplied by eukaryotes but lost by the ancestral archaebacterium. The idea that they originated from nowhere in the first eukaryote and were repeatedly laterally transferred into eubacteria [145] is bizarre, implausible, and not supported by trees (presumably proposed because the authors accepted the erroneous dogma that eubacteria are not ancestral to neomura). In two groups of actinobacteria the S/T kinases regulate cell growth and division including DNA segregation [146]. I suggest that this was also true of the stem actinobacterium from which neomura probably evolved, so there was even some continuity of cell cycle regulation between these posibacteria and eukaryotes. These posibacterial kinases have a binding site for peptidoglycan [147], which would have been lost when the ancestral neomuran replaced murein by glycoproteins allowing their recruitment for new cell cycle functions when the segregation machinery in the ancestral eukaryote was internalised as a consequence of phagocytosis (see next section below).

Though archaebacterial S/T kinases are more widespread [147] than previously thought [145], the apparent absence of cell cycle regulating PKN subfamily S/T kinases is yet another reason why archaebacteria, unlike Posibacteria, cannot be ancestral to eukaryotes. It is important to stress that as well as chromosomes mitosis segregates other organelles. Golgi duplication and centriole duplication are also regulated by some of these cell cycle kinases; so all segregation mechanisms must have coevolved. Polo-like kinases are essential for centriole and Golgi duplication (the latter by phosphorylating Golgi-nucleating centrin blobs [148]) and separase is important for centriole as well as chromosome separation in Euglenozoa [149], so these functions all evolved in the early eukaryote at the same time as the nucleus. Aurora and polo-like families are probably sisters; on the polo-like paralogue-rooted aurora kinase tree Euglenozoa are the most divergent eukaryotes [150], as they are for each of the four SMC subtrees [144] and on Fig. 1, giving further support to the conclusion that Euglenozoa are the earliest diverging eukaryotes [9]. In trypanosomes aurora kinase has more diverse and basic cell cycle functions than polo-like [151,152] suggesting that it might have been the original mitosis related kinase; the involvement of polo-like kinase in centriole duplication, mitochondrial DNA segregation and cytokinesis [153] suggests an early division of labour between the two kinases with respect to chromosome segregation (aurora) and cytoplasmic organelle segregation (polo-like). Proper geometric localization of the segregation machinery, exemplified by that of polo-like kinase along the presumptive division plane [154] would have been important in the early evolution of eukaryote cell division and would have been facilitated by the complex and semi-rigid character of the cell cortex supported by an extensive array of cortical microtubules; the rooting of the tree between Euglenozoa and Percolozoa (both of which ancestrally had rigid microtubule supported pellicle) plus a distinct cytostome for ingestion [9] confirms that the importance of cell surface rigidity for the evolution of organelle segregation that I have long emphasized; contrary to early ideas dating back to Haeckel the cenancestral eukaryote was not a formless amoeba but a phagotrophic flagellate with rigid cell cortex and localised ingestion apparatus for phagocytosis. That is the only way to reconcile the conflicting demands of surface rigidity for geometric control of segregation and surface fluidity for ingestion during the prokaryote to eukaryote transition. The transitional organism had simultaneously to perfect ingestion and digestion and maintain viability by reasonably accurate organelle segregation. Mutual attachment of almost every cell organelle is seen in such modern cells as trypanosomes [155-157] and the cercozoan *Sainouron *[158].

The contractile calcium-binding protein centrin and proteins that bind it play key roles in organellar positional control that are often more disparate and pervasive than its core centrosomal function. Trypanosomes have five centrins [156] with diverse functions including coordinating cell and nuclear division [159] and ciliates far more, mainly to construct their uniquely complex and pervasive contractile cortical infraciliary lattice [160]; some protists (especially those that lost centrioles and cilia) have only one, probably secondary simplification. Centrin forms a variety of structures and is related to other calcium-binding proteins that likewise have four EF-hand folds, notably the cytosolic calmodulin and ER lumen calreticulin; all three are present in Euglenozoa and clearly originated in the ancestral eukaryote from a common ancestor. Archaebacteria have no EF-hand proteins; those with a single EF-hand motif are very widespread in eubacteria [161], but multiple EF-hand motifs are much rarer, proteins with four as in eukaryotes being restricted to Actinobacteria, Proteobacteria, and Cyanobacteria. Very likely they came vertically from the stem actinobacterium but sequences are too short to test this adequately. Calnexin, a second ER calcium-binding protein, is absent from Euglenozoa [67], and probably evolved by gene duplication from calreticulin only in early neokaryotes. Centrin trees [162] show that at least two slowly evolving centrins were present in the cenancestral eukaryote, and other data suggest that one (the clade that includes human centrins 1, 2 and 4) was probably associated with centrioles and the other (the clade that includes human centrin 3) with spindle poles.

I previously argued that mitosis (i.e. DNA segregation by microtubules) evolved in two stages: a primitive system with only one microtubule nucleating centre (MNC) associated with the origin of replication of the ancestral neomuran organ of replication in which microtubules were constitutively present at the cell surface; and a more advanced one caused by the simultaneous origin of the nuclear envelope, centriole, and cilium in which there were separate cytoplasmic (permanent) and intranuclear microtubules (transient) each with their own MNC [4]. The initial function of microtubules was argued to be preventing the phagocytic internalisation of the DNA/membrane attachment sites (and consequent mis-segregation in the absence of mitosis) by providing a subpellicular corset of microtubules conferring surface rigidity in place of that abandoned when the bacterial cell wall was lost when prey ingestion evolved. The key merit of this two-stage evolution of mitosis was allowing continuity between bacterial segregation at the cell surface and intranuclear mitosis in the first protozoa [4]. The intermediate stage on the cell surface did not require major changes to the chromosome but would allow MNCs and microtubules to evolve with a function closer to ancestral TubZ (separating plasmid DNA at the cell surface) or FtsZ (constraining the plane of cell membrane division between daughter DNAs) than the much more complex intranuclear mitosis or cilia, but with the potential to evolve both later by adding extra functions. The likelihood that the two-stage theory is correct has been substantially increased by what has been learned since then about bacterial DNA segregation (outlined above) and by the rooting of the eukaryote tree between Euglenozoa and excavates [9]. As the earliest branching excavates, given that root position, are the Percolozoa (e.g. the amoeboflagellate *Naegleria*), the root of the eukaryotic tree is between them and Euglenozoa; we can therefore infer with confidence that any characters common to both of these protozoan phyla were present in their cenancestor and thus also in the eukaryote cenancestor. For example, Euglenozoa and Percolozoa are informally collectively known as discicristate protozoa because they each ancestrally had flat discoid mitochondrial cristae, which must have been the ancestral condition for eukaryotes; the more widespread protozoan condition of tubular cristae evolved later in the excavate Loukozoa. Mitosis in discicristates is always closed with a transient intranuclear spindle nucleated by an inconspicuous intranuclear MNC, whereas the cytoplasmic pellicular microtubules are present throughout the cell cycle and divide longitudinally by an antero-posterior cleavage furrow after the centrioles separate. Furthermore in all discicristates the nucleoli divide at mitosis in a dumbbell manner and do not disassemble and reassemble as they do in higher eukaryotes.

Thus the open mitosis of animals and of streptophyte green plants (i.e. embryophyte land plants and their charophyte algal ancestors) in which nucleoli and the nuclear envelope both fragment during prophase and are reassembled at telophase are clearly convergent secondary adaptations (which probably evolved for reasons of larger cell size and calcium control as explained elsewhere [163]) and positively misleading for understanding the origin of mitosis. Nucleolar fragmentation evolved in excavates after Loukozoa and Percolozoa diverged, but closed mitosis was retained by excavates and by the ancestral neozoa and ancestral unikonts (being retained by fungi and Apusozoa) and corticates (being retained by alveolates, red algae and many chlorophyte green algae). Open mitosis evolved independently from animals and streptophyte green plants within Amoebozoa among unikonts and in many chromist groups other than alveolates. It is notable that Eozoa and alveolates generally have more centrin paralogues than more derived eukaryotes, the most widespread number being four [162]. I suggest that the cenancestral eukaryote had three or four centrin paralogues: one in the nucleus for specifying mitotic poles and two or three in the cytoplasm for positioning the anterior microtubule nucleation centres for the pellicular microtubule corset, centriole(s), and Golgi apparatus. This many centrin foci at least is needed for the complex cytoskeletal architecture of discicristates. When higher fungi (ancestors of yeasts) lost centrioles and centriolar roots and their wall allowed the loss of pellicular microtubules [164,165], they unsurprisingly lost all centrin paralogues except that for the intranuclear centrosome (which was modified in appearance to form the spindle-pole bodies). Acentriolar Amoebozoa like the slime mould *Dictyostelium *independently lost the extra redundant centrins when their cells were morphologically simplified for amoeboid locomotion (not an ancestral character for eukaryotes). In green plants the ancestor of *Chlamydomonas *lost all centrin paralogues except one when it evolved semi-open mitosis, in which the nuclear envelope breaks down during mitosis only near the poles of the spindle allowing cytoplasmic centrosomal microtubules to penetrate the polar gap and contact the chromosomes that remain inside the rest of the NE [166]. The independence of this green plant centrin simplification from that of higher fungi also fits the fact that they lost the former intranuclear paralogue (unlike fungi, which retained it) and retained a cytoplasmic one for both spindle poles and centrioles [162]. In seed plants, higher gymnosperms and angiosperms apparently retained the same paralogue as *Chlamydomonas *for their cytoplasmic spindle poles when they lost cilia and centrioles [162]. I suspect that the clade of two paralogues labelled 'alveolate-specific' in [162] may not really be unique to that group; more likely they are related to one or both of the so-called group-specific branches for the Euglenozoa (trypanosomatids), Percolozoa (*Naegleria*) and Metamonada (*Trichomonas*; *Giardia *has only two so may have undergone some secondary simplification compared with other Eozoa) and that the long branches for all four groups prevent their grouping correctly. That each of these four groups convergently acquired multiple deep-branching paralogues seems improbable, especially as the presence of several deep-branching centrin paralogues in Eozoa is to be expected from the ancestral great complexity of their cytoplasmic organelles (Fig. 2). Alveolates are the only group of corticates that have extensively retained the ancestral discicristate cell organization of a separate permanent cortical array of pellicular microtubules and intranuclear spindle, which is almost certainly not convergent with that of Eozoa but a persisting ancient character; so their retention of more centrin paralogues than in plants animal, Amoebozoa or fungi is understandable. It should be noted that in these eozoan flagellates, as well as in apicomplexan alveolates, the cortical pellicular microtubules are nucleated at the cell apex and that the apical MNC duplicates prior to cell division just as does the intranuclear MNC; during cytokinesis the microtubules attached to the apical MNC separate as two half cones of microtubules geometrically similar to the much smaller intranuclear mitotic half-spindles. Thus cell division in Eozoa ancestrally was like dividing a Russian doll with a nuclear half-spindle (attached to the inner face of the inner nuclear membrane) nested within a larger half-spindle (attached to the inner face of the plasma membrane). The MNCs of both are mutually attached and their duplication and separation must be temporally coordinated. Paradoxically this very complexity of eozoan cell architecture makes it much easier to understand how mitosis evolved than if the first eukaryote was a formless amoeba with no cytoplasmic microtubules as many wrongly assume. Organelle segregation is a geometric and mechanical problem; architectural complexity and geometric order, mediated by semi-rigid mutual attachments of organelles, make it less likely to go wrong and conceptually simpler to understand than a more fluid or disordered system.

On my theory of the origin of mitosis [4] the larger cytoplasmic half-spindle is more like the ancestral prekaryotic version than the transient intranuclear mitotic one that evolved only at the time of chromosome internalisation, which must have been accompanied by the permanent duplication and differentiation of the MNC to give separate evolutionary divergent versions for plasma and nuclear membrane division: division of the two membranes was as important as division of the chromosome and all three had to be coordinated by direct physical attachments of each by cytoskeletal proteins. When the pellicular microtubular cones separate they are arranged side-by-side and not at 180° in mirror symmetry as are half-spindles in classical open mitosis of animals and plants (orthomitosis). However, in many protists with closed mitosis (e.g. some metamonads, some fungi like microsporidia, some Euglenozoa like trypanosomatids, most Retaria in the Rhizaria, and even one primitive green alga) mitotic half-spindles also are side-by-side in early stages and throughout most of mitosis (then called pleuromitosis), often only becoming oppositely oriented just before or as the cells separate. Thus the duplication and division mechanism of both are probably fundamentally similar, the main difference being to which membrane they are attached and that nuclear microtubules only transiently assemble. It is just as if a single MNC and microtubular cone became doubled and differentiated for two complementary functions, as my two stage theory proposed [4]. The theory is here improved by arguing (1) that discicristates retain both the original cell surface MNC and pellicular microtubules and the more derived intranuclear ones, and (2) that the differentiation went hand-in-hand with the membrane internalisation that generated the ER/NE. Surface pellicular microtubules of discicristates and their division mode were the mental model in mind when I proposed that microtubules first thus evolved at the cell surface [4], but because the tree was misrooted I did not then think (as I now do) that their cytoplasmic/cytokinetic/pellicular microtubular division mechanism probably descended directly from the hypothetical prekaryotic surface system, and is not just an analogy.

The two key constituents of centrosomes are gamma tubulin, the nucleator for microtubule assembly, and centrin for positional control and architecture. It is centrin, the positional controller that underwent gene duplication to several paralogues to enable the complex structure of the cenancestral eukaryote. However, in the prekaryote before the origin of nuclei and cilia a simple centrosome with just one centrin paralogue would have sufficed. When I first speculated about the origin of eukaryotic chromosomes [70] it was generally assumed that bacterial DNA segregation was passive, by wall growth between DNA attachment points, so I proposed that centromeres evolved from the termini of bacterial replicons, because of the requirement that DNA replication be completed before division and the idea that replication termination could physically directly signal this to a adjacent segregation machinery and wall division site. Later I decided that bacterial DNA segregation must be by active pulling apart of replicon origins by ATPase motors [141], now known to be true, and argued instead that the first microtubule nucleation site therefore probably evolved in association with the pre-existing binding sites on the cell membrane of bacterial replication origins, as shown in Fig. 3c. We now know that Par genes that are central to bacterial DNA segregation cluster close to bacterial chromosome origins and that ParS marks the site of loading Smc condensins [132,133]; the bacterial Par system is widely considered a centrosome analogue, so the idea that centromeres originated from the origin region of the bacterial chromosome is common place, albeit seldom made explicit. However at the prekaryote stage when chromosomes were still attached to the cell surface membrane and there was only a single MNC, the distinction between a primitive centrosome and primitive centromere was less clear; both functions could have been assumed by a single macromolecular complex at one point on the cell surface [141]. I considered the postulated single MNC to have been a proto-centrosome (not a centrosome) because microtubule nucleation and the division of MNCs had to evolve before there could be any selective force for the evolution of kinetochores to attach chromosomes to the growing end of microtubules. MNCs are much more fundamental and general that kinetochores because they are essential for the segregation of many other organelles as well as chromosomes and because all interphase cell architecture is organised around the various cytoplasmic MNCs and centrin foci and their attachments to the nucleus.

I assumed above that when the nucleus evolved there was only a single MNC at each spindle pole. However that might not be true, because in euglenoids there are several distinct sub-spindles in the nucleus [110] so they must have multiple MNCs at each pole. If the eukaryotic root were actually within euglenoids (e.g. between the unicentriolar *Scytomonas *and more typical bicentriolar genera) then this multiple MNC condition would have been ancestral for all eukaryotes. Even if the root is between Euglenozoa and neokaryotes it could still have been the ancestral condition and the uni-MNC condition of neokaryotes and Kinetoplastida/Diplonemea (the main other putative euglenozoan clade than euglenoids) could have arisen independently by polar MNC merger. Previously I argued that the polarity seen in animal epithelial cells, and to a lesser extent also in migratory mesenchymal cells [167] stems fundamentally from the cell polarity that evolved in the first phagotrophic zooflagellate [58]. With the root being beside or within the ancestrally zooflagellate Euglenozoa [9] we can now say that the basic pattern and mechanisms of such cell polarity go right back to the last common ancestor of all eukaryotes and that it is a universal principle of eukaryotic cell biology. Haeckel's idea that ancestral eukaryotes and cells generally were amorphous amoebae without centrioles or cilia [168] has mislead thinking for over 150 years. Skeletal organisation is fundamental to understanding eukaryogenesis; in the cytoplasm the cytoskeleton consists of cross-linked actin filaments and microtubules. The nucleoskeleton is primarily chromatin itself [169,170], heterochromatin in particular whose origin I next discuss. Actin is being found to be increasingly important also in nuclear functions of animals at least [171-173]. But as its role in Eozoa, if any is unknown, and even in animals actin's contribution to basic nuclear architecture is much smaller than that of chromatin, I omit discussing it, beyond pointing out that if it has similar roles in trypanosomatids, these must be of universal significance and must eventually be taken into account in fuller discussions of nuclear origins.

### Origin of Heterochromatin and Centromeres

Heterochromatin evolution is of key importance for the origin of the nucleus because the nuclear envelope is generally bound to at least a thin layer of condensed chromatin that would usually be called heterochromatin and bacteria have no equivalent structure [174]. Traditionally, constitutive heterochromatin was defined as chromatin that was condensed and microscopically visible throughout the cell cycle in contrast to euchromatin that dispersed in interphase and condensed only in mitosis as visible chromosomes. It has bizarre and largely unexplained patterns of variation in abundance and intranuclear distribution among organisms and developmental stages, but is often associated with centromeres, telomeres and the nuclear envelope. Until recently its function was a mystery; it was assumed not to be transcribed. All these ideas were oversimplified. Some organisms like budding yeasts lack visible chromatin condensation in both mitosis and interphase and others like euglenoids have visibly condensed chromosomes throughout the cell cycle. Heterochromatin generally has a low density of ordinary protein-coding genes; but much is transcribed at some stage of the life history to make transient non-coding RNA of varied functions. Long ago I postulated that heterochromatin is really DNA that has a largely structural role, such that its volume and degree of folding or unfolding can be modulated according to its needs - both in the interphase nucleus (where volume needs to be modulated at different developmental stages) and during mitosis where the compaction of DNA into centromeres and the geometric ordering of their kinetochore microtubule binding sites must be critical [169,175,176]. I also suggested that multicellular organisms may often transcribe it at stages when maximal nuclear volume was needed [169,170].

Recent ideas about heterochromatin have been dominated by the discovery that it is typically formed by the covalent modification of histones and is associated with an elaborate gene silencing machinery involving small 20-30-nucleotide non-coding RNA (microRNA or miRNA [177]) and associated enzymes that cut RNA that miRNA recognises by base pairing [178-181]. The significance of this vast body of work has been unclear for nuclear origins because although known in all higher eukaryotes (specifically Neozoa - all descendants of the last common ancestor of animals and plants) it was uncertain which phenomena and molecules were in the first nucleus. It is best known in the fission yeast fungus *Schizosaccharomyces pombe*, where RNA polymerase II transcribes heterochromatin (especially centric) only briefly during S phase and the RNA silencing machinery chops up the transcripts (recognised via double stranded loops) to make miRNAs which direct the histone deacetylating and methylating machinery for transmitting the heterochromatic state to the same locations in newly replicated DNA [182-185]; a form of epigenetic positional control. Plants use different specialized RNA polymerases (IV and V) for transcription and methylate DNA as well as histones to make heterochromatin, but the principle of RNA silencing and the machinery for generating and using small RNA is similar across all neozoa. Just enough is known about gene silencing and histones in trypanosomes to say that the basic gene silencing machinery was already present in the eukaryote cenancestor, but probably with substantially lower molecular complexity than in animals. As in neozoa, all four core histones are acetylated and methylated (sometimes at homologous sites) in trypanosomes [186], and a limited number of homologous acetylating, deacetylating, and methylating enzymes (mostly targeting lysines) exist; there are also a few chromatin remodelling enzymes and histone-binding proteins with bromodomains, PDH fingers and Tudor domains, so this basic machinery all evolved in the ancestral eukaryote. There is also clear evidence for telomere silencing, which presumably evolved in the ancestral eukaryote when telomeres arose making chromosomes linear. But it is unclear whether their core histones are phosphorylated or ubiquitinated; though core histone phosphorylation by aurora kinase can occur in vitro in trypanosomes, it is likely that the mechanisms and their uses are simpler in Euglenozoa.

In neozoa, heterochromatin assembly at the centromere is typically a prerequisite for the loading of CenpA onto kinetochores and therefore for binding spindle microtubules (i.e. in all but budding yeasts (*Saccharomyces*), which secondarily evolved point centromeres where a different histone variant Cse-1 is loaded directly onto centromeric DNA in association with proteins able directly to recognize the centromeric DNA sequence [187]; model systems are often the exception to the rule, as for *E. coli *and SMCs). Neozoan heterochromatin is marked by heterochromatin protein 1 (HP1) which recognises lysine 9 (K9) in histone H3, directing methyltransferase Clr4 to methylate it. However trypanosome H3 lacks lysine K9, having four fewer amino acids in this region, so like *Saccharomyces *they must also use different machinery for assembling heterochromatin. As archaebacterial histones [188] lack the N- and C-terminal tails that in eukaryotes are important for the chromosome condensation cycle and which are modulated by acetylation and methylation, the slightly shorter N-terminal tails of trypanosomatids plausibly represent the ancestral eukaryotic condition, but until we know more about H3 and H4 in other Euglenozoa we cannot be sure that they are not secondary deletions. Although archaebacteria do not acetylate histones, they do acetylate their other major chromatin protein, Alba [188] which is a prerequisite for Mcm function in replication [189]; Alba probably evolved from a more universal RNaseP protein [190]; deacetylation is done by a homologue of the eukaryotic Sir2 histone deacetylase, so the acetylation/deacetylation machinery was in place in the neomuran ancestor before the origin of the nucleus and could be co-opted as soon as histone tails were added (probably the key step in evolving eukaryotic chromatin) and gene duplication produced H2A and H2B and octomeric nucleosomes and the associated capacity for reversible condensation, for which histone H1 modifications are also important (in neozoa at least).

Gene silencing depends on small non-coding RNAs that bind to a protein of the Argonaute/PIWI family to form a complex that recognises targets for destruction or repression [191]. Also always involved are homologues of RNase III, an endonuclease that in all organisms cuts prerRNA during rRNA formation [192]. Some bacteria have Argonaute proteins [193,194], so both key enzymes were present in the neomuran ancestor before eukaryotes evolved. Moreover archaebacteria have a variety of small non-coding RNAs [195]; of these, small nucleolar RNAs (snoRNAs), which guide the processing machinery for prerRNA in all neomura (both for methylation and other modifications of nucleotides and cutting the backbone) [196,197] evolved in their cenancestor as did the protein fibrillarin - the catalyst of prerRNA methylation and a core protein of nucleoli, the part of the nucleus where prerRNA is processed and ribosomal subunits assembled (after their cytoplasmically made proteins are imported into the nucleus). Bacterial RNase IIIs all have a single RNase III domain (as do at least some of those that process prerRNA in eukaryotes [198]) and thus function as homodimers so that the two strands of the target double-stranded RNA undergo staggered cuts by separate active centres. The RNase III eukaryotic homologues involved in gene silencing (the most ancient one is usually called dicer) have two adjacent RNase III domains, produced by internal tandem duplication of a gene duplicate of class I RNase III in the ancestral eukaryote, which can cut both strands as a monomer [199]. During gene silencing dicer generates 21-23 nucleotide fragments. Dicer of neokaryotes has an N-terminal RNA helicase domain and a central PAZ domain as does Argonaute, but the two dicer-like RNase IIIs of trypanosomes lack both domains, whereas the excavate *Giardia *has the PAZ domain but not the helicase domain [200]. I suggest that the simple situation in trypanosomes (Euglenozoa) which is most like the ancestral neomuran class RNase III was the ancestral condition for dicer and that the PAZ domain was added in an early excavate and the helicase domain in the ancestral neozoan. This progressive increase in complexity of dicer agrees perfectly with the tree topology of Fig. 1 (with the first eukaryotic divergence between Euglenozoa and neokaryotes), and means that extra dicer functions were added during eukaryote evolution and we must be cautious in generalising functions so far demonstrated only in neozoa to other eukaryotes and to their cenancestor.

What then was the first function for these small RNAs? In neozoa three functions are known: developmental regulation by gene silencing (either during or after transcription); destroying transcripts of viruses or endogenous retroelements; heterochromatin formation (and possibly other aspects of chromosome structure or stability). It has been suggested that defence by destroying RNA of parasitic genetic elements may have been the first function [201]. However, I do not find that plausible because the need for such defence has been present for billions of years and dicer did not evolve in bacteria despite suitable precursors being present. Developmental regulation also is a primordial need, not specifically eukaryotic. We must explain why dicer-generated small RNAs evolved specifically during eukaryogenesis. Facilitating heterochromatin formation is the most obvious function that does this, but others connected with eukaryotic novelties are also possible. I originally proposed that heterochromatin evolved first for folding centromeric and telomeric chromatin appropriately and then spread to interstitial chromosomal regions by the possibly selfish transposition of heterochromatin labelling sequences - the former to provide a strong enough selective advantage to evolve a complex machinery and the latter to explain the apparently haphazard distribution of interstitial constitutive heterochromatin [175]. Later I argued that the primary selective force for the origin of heterochromatin was probably the folding of centrosomal histones to allow accurate DNA segregation by mitosis [18]. Small RNAs could provide a sequence-specific mediator between heterochromatin determining sequences and heterochromatin establishing proteins, whether facilitators of posttranscriptional modification of histones or of the loading of special proteins onto those regions. In neokaryotes, heterochromatin structure itself is the prerequisite for loading the centromeric histone CenpA onto adjacent DNA [202]; small RNAs are not themselves needed; their function is simply to ensure that centromeres form in the right place. As heterochromatin formation mechanistically precedes CenpA loading in neokaryotes but CenpA is absent in Euglenozoa, centromeric heterochromatin probably evolved before CenpA and was, I postulate, the primary determinant of centrosome function in early eukaryotes and probably evolved in the prekaryote during the origin of mitosis prior to the cell nucleus. Trypanosomes do use both histone methylation and deacetylation in cell cycle related controls [186,203] and Argonaute (and thus small RNAs) is essential for proper DNA segregation [204] (I suggest through controlling centromeric heterochromatin) and some trypanosomatids use small RNAs for suppressing transposons [205] and silencing their own genes [206]. However their canonical case of gene silencing is of genes located near telomeres that probably exploited a pre-existing telomeric gene silencing machinery (see next section). In trypanosomes one Argonaute protein is used for controlling chromosome segregation and also mRNA in polysomes for posttranslational control [205], while another targets exogenous RNA [207]. Probably the first eukaryote had a single Argonaute [208] primarily concerned with heterochromatin assembly. The heavy emphasis on translational control in trypanosomes is not likely to be a primitive character, but is probably a secondary consequence of the evolution of polycistronic messengers and universal trans-splicing of mini exons to pre-mRNA in the ancestor of Euglenozoa [59,209-211], coupled with the virtual abandonment of transcriptional control, which must have occurred after Euglenozoa diverged from neokaryotes. The fact that gene control in neokaryotes is largely transcriptional as in prokaryotes means that this must have been true also of the eukaryote cenancestor. Likewise the use of histone variants to label the boundaries of polycistronic transcripts in Euglenozoa [212,213] is almost certainly a secondary innovation for them, but it could have evolved from a more basic early eukaryotic use of histone variants to label functional chromosome domain boundaries.

I suggest that gene repression by small RNAs (miRNAs) and suppression of exogenous viral or transposons transcripts by destruction via analogous small silencing RNAs (siRNAs) could both easily have evolved and be applied to a great variety of targets according to the specific needs of different lineages after the basic small RNA-based machinery evolved for centromeric assembly and was modified for telomeric chromatin. Both latter functions are general for all eukaryotes, but specific gene repressions such as mating type silencing in yeast or variant surface antigen repression in the parasites *Giardia *and trypanosomatids are lineage-specific exploitations of a more basic pre-existing machinery. Various eukaryotes have secondarily lost the capacity for suppressing exogenous RNA by siRNA (also called RNA interference), e.g. *S. cerevisiae *([214], but related budding yeasts have retained it [215]) and *Trypanosoma cruzi *(but *T. brucei *retains it). That such organisms still have some heterochromatin suggests that the origin of heterochromatin was more complex than I have implied; study of its types and functions in Euglenozoa broadly are important for clarifying its origin. The secondary origin of centromeres by direct binding of centrosomal proteins to specific DNA sequences (bypassing typical heterochromatin) that evolved in budding yeasts may have predisposed some of them (e.g. *S. cerevisiae*) to lose the standard siRNA mechanism, as purifying selection for efficient centromere function would no longer retain it.

A more broadly distributed form of miRNA that probably evolved almost as early as centromeric small RNAs is miRNAs derived from snoRNAs, which arose at least as early as excavates, being found in *Giardia *as well as across neozoa [216]. One potential hypothetical function for snoRNA-derived miRNA that might be universal would be the positional control of nucleolar heterochromatin which is present in most eukaryotes and likely to be important in nucleolar architecture. snoRNAs evolved in the ancestral neomuran and come in two classes, boxC/D snoRNAs that recognize sites for methylation (or in one case - U3 - proper folding of prerRNA for cleavage [217]) and boxH/ACA snoRNAs that recognize prerRNA sites for isomerization to pseudouridine; each interacts with a different 4-protein catalytic complex. They underwent little change during the origin of the nucleus; trypanosomatid and euglenoid snoRNAs are notably simpler than those of neokaryotes (including the excavate *Giardia*) and more closely resemble those of archaebacteria [218-220], giving additional strong support to the primary eukaryote divergence being between Euglenozoa and neokaryotes (Fig. 1). Unsurprisingly on this view of the root, U3, which is far more variable in length than other eukaryotic snoRNAs, is shortest in trypanosomatids [217].

### Origins of telomeres and telomeric heterochromatin

The primary reason d'être for telomeres was to solve the end replication problem of linear chromosomes [221] and the telomerase machinery could in principle have been recruited either from host enzymes or from exogenous selfish genetic elements [70,222]. Linear chromosomes have evolved several times in viruses, bacteria, and mitochondrial DNA as well as during the origin of eukaryotes and the solution to the end replication problem has been varied. Probably in most cases the original fragmentation of the once circular chromosomes was accidental and all mechanisms were ad-hoc rescues. The origin of linear eukaryotic chromosomes may have occurred in the prekaryote prior to the origin of the nuclear envelope if meiosis (see below) began then, since odd numbers of meiotic crossovers or mitotic sister chromatid exchanges in circular chromosomes produce circular dimers, resulting in accidental breaks during DNA segregation [11,70]. Even before the origin of the nucleus telomeres would have acquired additional proteins to help block accidental covalent joining of ends of different chromosomes; a key protein involved in this end protection is Rap1 [223] that must have been present in the ancestral eukaryote as it present in trypanosomatid telomeres, where it is also essential for silencing genes located in telomeric heterochromatin [224,225]. Telomeric and centromeric gene silencing may both be by-products of the assembly of heterochromatin. Telomeric heterochromatin depends not only on region-specific proteins like Rap1, but also on others shared with centromere biogenesis; histone acetylases are involved [203] and the mi-RNAs derived from snoRNAs [216] potentially target the telomere-located variant surface antigen proteins. If this is also true of Euglenozoa it could mean that snoRNA derived miRNAs were recruited for telomere heterochromatin formation even in the first eukaryote. However, when the nuclear envelope evolved (see next two sections) telomeres would have acquired additional functions for attaching to it. Both in interphase and meiotic prophase telomeres are normally attached to the nuclear envelope. Probably the compacted nature of both centromeric and telomeric heterochromatin were prekaryotic preadaptations that would have facilitated the attachment of endomembranes to the surface of chromatin, the central evolutionary innovation in the origin of the nuclear envelope. The nucleolus also typically has associated heterochromatin, which also might have evolved before the nucleus and might as suggested above depend on small RNAs for its positional control.

### Phagocytosis, endomembranes, mitosis and novel cell cycle controls

It is often overlooked that internalisation of DNA-attachment sites by primitive phagocytosis would automatically produce an endomembrane vesicle with the chromosome attached to its surface by bacterial membrane proteins that bind DNA [4]. Almost certainly some such proteins were retained for similar DNA-binding duties in the inner membrane domain of the NE during eukaryogenesis. Likely candidates are Man1 (its MSC domain is related to the DNA-binding helix-turn helix fold of many bacterial transcription regulators) and RfB [14,17]. There had to be much continuity in DNA attachment and segregation processes during eukaryogenesis, despite its radical nature, or cells would die [4]. This DNA-bearing endomembrane was the NE precursor [4]. Such internalization of DNA must immediately have caused segregation problems [4,70], for any membrane division machinery carried with it might divide the endomembrane after DNA replication, yielding two daughter DNAs attached to separate cisternae, these would not be attached to the cell surface, which initially might still divide by an FtsZ ring. Without direct physical coordination of division of surface and endomembranes, daughters lacking DNA or having multiple chromosomes would inevitably be produced at a fair frequency until protoNE/DNA segregation became less random [3,4]. Avoiding this was the primary selective force for the radical origin of mitosis [3,4,70]; as suggested above the difficulty of the transition may have been somewhat mitigated by the transitional intermediate being multigenomic, so unequal divisions would produce DNA-free cells with lower frequency than if there were only one genome per cell. Provided that the transitional cell had a uniquely strong compensating advantage that completely set it apart from all bacterial competitors (eating other cells) it could still enjoy net reproductive success and rapid population growth despite the handicap of generating a proportion of DNA-less daughters.

Once it achieved this degree of success the main competition would be among its offspring, leading to increased segregational efficiency of the best lineage, with all the less efficient ones dying out. This same principle would apply to all innovative aspects of eukaryogenesis, ensuring that there was probably only one eventually successful lineage surviving the transition, with no half-evolved lineages persisting long enough to become ecologically important. Mis-segregation could be avoided only by coordinating division of both membranes through novel indirect physical connections to the cell surface. As the surface skeletal bacterial protein MreB had already undergone duplication and evolved into actin and Arps for phagocytosis, other gene duplications produced Arps to nucleate a contractile actomyosin ring for dividing the surface membrane [6]; even in eubacteria the divisome includes an actin relative, FtsA [226]. Membrane division also needs membrane deforming and membrane scission proteins. It now appears that novel proteins of this sort evolved during the neomuran revolution when the peptidoglycan wall was replaced by surface glycoproteins. In eubacteria it is presumed that the actual membrane scission might involve the growth of the wall and formation of the new murein septum, though all the machinery involved has not been identified [227]. Obviously that could not continue when murein was lost. Instead ancestral neomura evolved a novel membrane deforming and scission machinery using proteins ancestral to both the ESCRT III complex of eukaryotes [228] and the CdvA, B proteins of (mainly) crenarchaeote archaebacteria [229,230]. In eukaryotes this is involved in membrane division during cytokinesis and endosome division and in archaebacteria in cytokinesis and membrane blebbing into the environment [231]. The mesophilic former crenarchaeotes now called Thaumarchaeota [232] retain both the old FtsZ and the new ESCRT-like division machinery (which probably have complementary functions), but hyperthermophilic crenarchaeotes lost FtsZ (and so could not be ancestral to eukaryotes unless eukaryotes got their tubulin ancestor from FtsZ mitochondrial ancestor - very unlikely) and retained only ESCRT-like division machinery (Thermoproteales evolved a third unknown mechanism for their odd snapping division), as did eukaryotes (assuming tubulin evolved from TubZ not FtsZ). Conversely euryarchaeotes except *Thermoplasma *lost ESCRT-like division proteins. Another membrane division novelty, the large GTPase dynamin, which promotes division by forming a helical collar around the neck of constricted membranes, evolved later in the ancestor of eukaryotes only. Many dynamin paralogues evolved for different functions: cytokinesis, vesicle budding from the Golgi, endocytosis, peroxisome (probably all before the origin of the nucleus during endomembrane diversification [27]) and also for nuclear fusion (see below); and later mitochondrial division, and even later a plant cytokinetic one was further duplicated to help chloroplast division [233].

Another likely consequence of murein loss was a basic change in the termination of DNA replication. The ancestral mechanism in eubacteria is DNA site-specific and tightly linked to murein septation, and involves a recombinase (Xer in proteobacteria) that unlinks catenanes by site-specific recombination at the terminus [234,235], together with a DNA translocase (FtsK in proteobacteria) that strips proteins from DNA and helps unlinking [236]. This machinery was lost in the ancestral neomura, which all use non-site specific DNA termination mechanisms and different ways of controlling DNA replication in relation to the cell cycle, including novel topoisomerase II enzymes that can separate intertwined sister DNAs. As argued before [12], many novel features of neomuran DNA replication machinery and its control stem from the origin of histones rather than the loss of murein in the cenancestral neomuran. This is arguably so for the new preinitiation machinery involving Mcm proteins and Cdc6 discussed below.

Triplication of a TubZ-like gene in the ancestor of eukaryotes alone to yield γ-tubulin (centrosomes) and α- and β-tubulins making microtubules [3,6] provided a new machinery for DNA segregation, but as Fig. 3 indicated an initial key role may have been surface stabilisation to prevent DNA internalisation by phagocytosis. Initially there was no need for kinetochores and microtubule attachment to DNA since centrosomes and chromosomes were both bound to the membrane (Fig. 3c). DNA and endomembrane would automatically be cosegregated if microtubule polymerisation pushed daughter centrosomes apart [3,6], as is postulated for TubZ. Later, efficiency increased by evolution of kinesin motors to actively slide apart antiparallel centrosome-to-centrosome microtubules (Fig. 3c), and probably later still of dyneins to move vesicles and chromosomes towards the minus end of microtubules (Fig. 3e, f). Thus all major elements of mitotic spindles, possibly remaining through interphase as stable half-spindles, probably evolved before the nucleus [3,4,6], though I suspect that dynein, whose sister paralogue is midasin (a giant protein involved in rRNA export from the nucleus) may only have evolved at the same time as the nuclear envelope; most dyneins are concerned with ciliary function. I reserve discussion of the origin of centrioles (whose basic function is generating cilia) and cilia themselves to a later paper because of its complexity and because they are not fundamental to mitosis, being just 'there for the ride' [237-240], even though they originated prior to the eukaryote cenancestor. That FtsZ starts to assemble at the time of initiation of DNA replication [241] is intriguingly similar to the synchronization of centriole duplication at the beginning of S-phase; might the timing of γ-tubulin's initial assembly have been inherited from bacteria? By contrast to the more optional centrioles, as soon as protoER/NE became a distinct genetic membrane [85], its segregation without loss was as important for viability as that of DNA [163].

Efficient segregation and avoidance of DNA breakage (whether by entanglement with molecular motors effecting chromosome or vesicle movement or by contraction by the new actin contractile ring) required greater chromosome compaction [4,11,70]. Thus histone H3 and/or H4 (both arose in the neomuran ancestor [12,13]) underwent duplication and major modification, yielding histones H2a and H2b, forming octomeric nucleosomes, and an H1 precursor already present in the actinobacterial ancestor (lost by archaebacteria) [12], was recruited to link them as solenoidal 30 nm chromatin fibres. Methylating, phosphorylating, and acetylating enzymes were recruited and temporally coordinated to effect a chromosome condensation cycle (aurora kinase phosphorylates opisthokont H3 and Cdk1 does for H1 [242]), with looser interphase transcribed chromosomes alternating with inactive mitotic chromosomes of 30 nm fibre loops tightly folded around a proteinaceous core, including especially DNA topoisomerase II, essential to relieve positive supercoiling during transcription and to separate tangled sister DNAs. A further histone duplication generated CenpA, the core constituent of centromeres. It was previously assumed that this allowed evolution of specific microtubule binding directly to chromatin for the first time and thus fully developed mitosis and that it arose in the cenancestor of eukaryotes [12]. However, the rerooting of the eukaryote tree between Euglenozoa and other eukaryotes makes it possible that the apparent absence of CenpA in kinetoplastid Euglenozoa is the primitive state for all Euglenozoa and eukaryotes generally and that CenpA evolved only in the ancestor of neokaryotes (all eukaryotes other than Euglenozoa: [9]); CenpA homologues are present in excavates, including the earliest diverging Percolozoa, e.g. *Naegleria *[212]. Given that minichromosomes or yeast and trypanosomes can be segregated accurately without all the machinery associated with typical large chromosomes the ability to move organelles bipolarly on spindles is probably more basic and general than the specific modes of attachment. As suggested above the most fundamental innovation for centromeres may have been the origin of properly folded centromeric heterochromatin assembled at positions controlled by the base pairing of small RNAs. The requirement of Argonaute in trypanosomes for segregation of both mini and ordinary chromosomes [118], which do not have the same kind of kinetochores (and neither has CenpA), supports this.

That separase is needed but cohesin is not for trypanosome minichromosomes whereas both are needed for the large chromosomes [149] confirms the multiplicity or redundancy of the segregation machinery, as does its ability to segregate other organelles such as centrioles, Golgi, mitochondria and the nuclear envelope ER; in many protozoa the singleness and nuclear attachment of peroxisomes means that they also must be attached to the segregation machinery [158]. That segregation of these organelles may depend on similar mechanism to chromosome disjunction is shown by mammal cohesin being involved in centriole adhesion and separase cleavage of Scc1 (perhaps recruited to centrosomes by protein Aki1) causing their separation [243]. As separase mediates centriole separation in both trypanosomes and mammals, cohesin was probably recruited for attaching parent and daughter centrioles when centrioles first evolved prior to the eukaryote ancestor (between stages e and f on Fig. 3). The evidence that eukaryotes were ancestrally biciliate and that even the cenancestor had evolved ciliary transformation from a younger anterior to an older posterior cilium [9] means that proteolytic separation of mutually attached old and next generation centrioles must date from then. It is remarkably simplifying that chromosomes and centrioles use the same mechanism for adhesion and separation despite their fundamentally different structures and modes of duplication.

Very little is known about the molecular mechanisms of mitosis in Euglenozoa, except that mitosis differs cytologically in several respects from that of neokaryotes as well as among the euglenozoan classes, always lacking a chromatin condensation cycle (permanently condensed in euglenoids, permanently diffuse in kinetoplastids); in trypanosomatids there are often both minichromosomes (often more than the number of spindle microtubules so their segregation is unconventional); euglenoids also can have large numbers of small chromosomes; localised sites for binding DNA topoisomerase II may correspond with centromeres in some but not all trypanosomatid chromosomes [212,213]. Comparative studies of molecular mechanisms in Euglenozoa should greatly clarify the origin and early evolution of mitosis.

Two other key features of the eukaryote cell cycle [244] probably arose at about the same time as centromeres: DNA replication licensing and pervasive anaphase proteolysis and cycle resetting. Internalisation of DNA by phagocytosis would have disrupted not only the spatial coordination of DNA segregation but also the temporal coordination of replication and cell division as practised by bacteria [70]. This would have immediately been exacerbated by the origin of centromeres, because efficient mitotic DNA segregation totally removed the ancestral stabilizing selective force requiring only a single replicon per chromosome so replication termination could directly signal division to occur directly between the only two daughter replicon termini [70]. Thus inevitable recurrent mutations multiplying replicon origins or termini previously stringently removed by purifying selection for >2 Gy would suddenly be harmless and spread like wildfire [70] (and be beneficial, as multiple replicons would ensure more rapid completion of replication long before division might try segregating incomplete daughters and abolish wasteful overlapping rounds of replication [70]). Likewise mutations making several separate chromosomes would spread automatically, as mitosis, unlike bacterial systems, can cope with many [70]. Furthermore, rapid replication of numerous replicons removed any constraint for circularity arising from the need for replication forks to converge on a single terminus and signal completion, making mutations for linearity less harmful [70]. Thus, the characteristic multi-replicons of eukaryote chromosomes (and in part their linearity) are consequences of the origin of mitosis plus mutation pressure, not positive selection [70]. Having multiple replicons caused problems for ensuring that all replication was complete before division. The two solutions were evolution of Mcm-based DNA licensing to ensure all replicons initiated together, and of cyclins and recruitment of protein phosphorylation, dephosphorylation and proteolysis to coordinate this with cell growth, so that on average a round of replication was initiated at every doubling of cell size, with mitosis being inhibited till its completion. Mcm proteins themselves evolved earlier in the neomuran ancestor as they are also present in archaebacteria [245], but not apparently eubacteria, and work as a six-protein DNA helicase that moves replication forks. In archaebacteria and in the early diverging kinetoplastid Euglenozoa [246] replication origins are recognised by the protein Cdc6, which with the help of a partially related protein Cdt1 loads the Mcm hexamer onto replication origins. In neokaryotes the system is more complex: an additional prereplication complex consisting of up to six different origin recognition complex (ORC) proteins is also required. A major domain of five ORC proteins (Orc1-5) is related to Cdc6. I proposed that at least two of them (Orc1 and Orc2) evolved by gene duplication from Cdc6 in the ancestral neokaryote after it diverged from Euglenozoa [9], but that Orc4 (apparently missing from *Naegleria*) evolved only after the divergence of Percolozoa from other eukaryotes. Orc6 evolves faster and its origin is less clear. It thus appears that not all features of the ORC system that are universally present in higher eukaryotes (Neozoa) were present in the first eukaryotes and that the neozoan cell cycle control system probably evolved in stages during the successive divergences of Eozoa. Direct biochemical studies of excavate and euglenozoan cell cycle controls are essential to test this and elucidate the various steps.

Proteasomes, which probably arose in thermophilic actinobacterial ancestors of neomura to degrade denatured proteins [13], were complexified in the ancestral eukaryote by numerous gene duplications and access to them for destruction became controlled by the novel ubiquitin system and they were recruited for anaphase proteolysis. Numerous proteins, including especially the cohesin loops [247-249] that hold sister DNA molecules together from replication onwards (even in bacteria; they are also Smc proteins related to condensins) [63], were thus destroyed at the anaphase transition: the key switch of the eukaryote cell cycle [244]. It may be that this system is simpler in Euglenozoa and did not evolve all at once; one difference is that unlike in higher eukaryotes trypanosomatid Cdc6 is not degraded at S phase and persists in association with chromatin throughout the cycle [250].

Later, additional checkpoints blocking mitosis until all was ready were added. Attempts to deduce the order of evolution of cell cycle kinases from paralogue trees [251] are hopeless, as trees are obviously dominated by systematic biases caused by the radically different ways each family adapted immediately after gene duplication, against which statistical tests provide no protection whatever. Kinase trees do not remotely reflect time, are almost certainly misrooted (a pervasive problem with paralogue trees [13]), and likely to be topologically wrong. Fig. 3 of [251] has no valid outgroup and is simply 'rooted' among the three longest branches; cdc28 may appear late merely because since the cenancestor it evolved 2-3 times slower than them. All we can safely conclude from those trees is that all major mitotic and meiotic controls existed before the cenancestor (and the subsequent relative evolutionary rates of each paralogue). However gene duplication order is less important than the principle that the pervasive selective force of avoiding mis-segregation and DNA breakage made the eukaryote cell cycle, which was a rescue operation after the genetically immensely disruptive effects of DNA/membrane internalization by phagocytosis, which arose because of its immense trophic benefits despite this harm. Thus much of eukaryogenesis occurred to find a new equilibrium in which the benefits of phagocytosis could persist without its severe genetic costs. It is not intrinsically better to divide cells the eukaryote rather than the prokaryote way, but if DNA is attached to endomembranes instead of the cell surface there is no other option but a radically new solution, which was clearly constrained by the possible precursors that happened to be available and the new cell structure.

Thus, prior to compartmentation that finally made the nucleus, many features of eukaryotic chromosomes, including the chromatin condensation cycle and novel replication controls and novel segregation machinery, had probably evolved as an indirect consequence of the changeover from surface membrane to mitotic DNA segregation. As soon as the novel replication and other cell cycle controls were in place (necessitated to complete replication well before mitosis which is sudden at anaphase and not gradual and spread out over the whole cell cycle as in bacteria) accidental duplications of replicon origins inevitably spread them across the whole chromosome, but this would also have been positively selected as simultaneous replication at many points could compensate for the much slower movement of replication origins though nucleosomes and allow shorter cell cycles than otherwise); overall replication time would no longer limit genome size. There is no reason to think that an increase in genome size per se, which in eukaryotes is independent of organismal complexity [17], or quantitative population genetic factors such as effective population size were determinants of such a radical change in genome organization, as is sometimes claimed [79]. The above concentrated on the cell's trophic phase, but the cenancestral eukaryote also had resting cysts [3]. The evolution of syngamy and meiosis were probably associated respectively with encystation and excystation; a later section argues that the fundamental reason for the origin of sexual cycles with haploid and diploid nuclei was the conflicting selective forces during growth and dormancy (cell multiplication and survival respectively). However, I first consider the next steps in the origin of the nucleus of trophic cells, even though in reality sex-related chromosomal evolution was probably concurrent with the evolution of interphase nuclear structure.

### Nucleocytoplasmic separation: a two phase evolution

**Figure 5:**
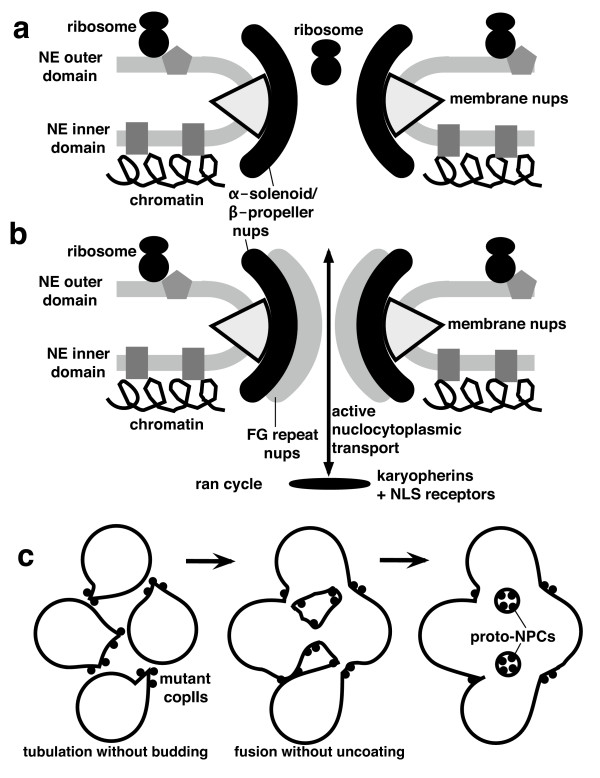
**Two-phase origin of the nuclear envelope and trans-envelope transport**. (a). Nucleoporins (Nups) forming the octagonal cylindrical scaffold evolved by duplications of coat proteins of COPII secretory vesicles with α-solenoid and/or β-propeller domains, being attached by integral membrane Nups descended from actinobacterial membrane proteins; (b). NPC lumens were narrowed by plugs of FG-repeat-rich Nups, which form a dynamic gel-like meshwork that prevents passive diffusion of macromolecular complexes and mediates active specifically-targeted nucleocytoplasmic exchange by carrier complexes, typically consisting of large karyopherin proteins and their cargo either bound directly or by adaptors. (c). Phase I surface view, showing complete Ran GTPase-mediated fusion of RER cisternae prevented by COPII coat proteins (black blobs) remaining in place to become octagonal NPC scaffolds.

NPCs must have evolved in two stages with different selective advantages [5]. Initially the basic octagonal cylinder embedded in the NE evolved, primarily to prevent complete fusion of ER cisternae (Fig. 3d) around chromatin, which would have been lethal by preventing transcripts reaching the cytoplasm and stopping growth [4,5]. Later this wide cylinder, allowing nucleocytoplasmic exchange by passive diffusion (Fig. 5a), was constricted by inserting the inner FG repeat nucleoporins (Nups) to exclude ribosomes from the nucleus and DNA and RNA polymerases and RNA processing complexes from the cytoplasm, and active transport machinery evolved to export and import them (Figs 3e, 5b). FG repeat proteins have long domains consisting of numerous repeats in which the basic amino acid phenylalanine (F) and the acidic amino acid glutamic acid (G) predominate, leading to a structure that is far less ordered than in most proteins. While probably a little more ordered than random coils, the FG-repeat Nups appear to form a hydrophilic meshwork with dynamic properties that impede the spontaneous diffusion of large macromolecular complexes through the NPCs, but can be modulated by the temporary binding of karyopherins [252] that can thus force large macromolecular complexes bound to them through the FG repeat putative hydrogel. FG repeat Nups not only dominate the central plug of the NPCs, but are also prominent in the nuclear basket that caps the NPCs on their nucleoplasmic face and in the long cytoplasmic filaments that help to control ingress to the nucleus nuclear on the cytoplasmic side. A gradient in binding affinity between karyopherins and the FG repeat Nups from weak outside to strongest on the nuclear basket is instrumental in the efficient polarised import of karyopherins and their cargos [253]. A basic principle of the evolution of the NPCs is that karyopherins and FG repeat Nups must have coevolved, as must karyopherins and their disparate cargos.

Extensive gene duplication and domain shuffling generated the two extensive and structurally different but functionally complementary protein families, the trans-envelope shuttling karyopherins and the FG-repeat Nups. The selective force for the coupled restriction of free diffusion of larger molecules and of active transport across Npcs was the benefit of compartmentation and specialization; higher concentrations of protein synthesis enzymes in the cytoplasm only and, especially nucleic acid synthesis enzymes in the nucleus could be maintained at much lower cost [4,5]; especially important in reducing cost was the effective exclusion of nuclear proteins from the much larger cytoplasmic compartment, thereby reducing the biosynthetic load on the cell.

I previously suggested that NPC transport machinery might in part have arisen from that for secretory vesicles [5,70]. This seems partially true. The basic octagonal scaffold Nups have α-solenoid and/or β-propeller domains clearly related to those of coated vesicles (COPII from ER; COPI from Golgi; clathrin-associated adaptins from plasma-membrane and endosomes) [15-17]. I now suggest that the NPC scaffold evolved from COPII coats, probably before COPI evolved; at that time COPII vesicles would be uncoated and fuse directly with the plasma membrane or protoendomembranes, processes mediated by small GTPases, that themselves underwent differentiative duplications in concert with those of vesicle coats that multiplied the number of genetically distinct endomembranes. Two β-propeller proteins (Sec13/She1) actually form a subcomplex in both COPII coats and NPCs [15-17]. β-propellers have several vanes composed of WD repeats that are widely present in proteins involved in binding other proteins (some in bacteria and many in eukaryotes). The combination of β-propellers and α-solenoid domains that is found in several core Nups is unique to eukaryotes; as such proteins are centrally involved not only in NPCs, and coated vesicles, but also in ciliary transport particles that supply growing ciliary axonemes with newly made proteins (and had also already evolved prior to the eukaryote cenancestor), their origin and diversification by gene duplication and domain shuffling were centrally important for eukaryogenesis. Since this paper was written the close structural relationship between the structural scaffold of the nuclear pore complex and the COPII outer coat lattice has been directly demonstrated by solving the crystallographic structure of the Nup85 20 α-helix/Seh1 β-propeller complex, placing their evolutionary relationship beyond reasonable doubt [254].

The universal binding of the nuclear envelope to chromatin during interphase is a really fundamental feature of the nucleus of profound importance for the evolution of eukaryote genome size and for the explanation of why eukaryote genome size correlates with cell volume [18], but has been largely overlooked by geneticists and theorisers about nuclear origins unfamiliar with cell biology. It was crucial for nuclear envelope evolution, because its inner membrane and components of the NPC are bound to chromatin and because the binding of RanGTP to chromatin, and the exclusion of the ancestrally soluble RanGAP that mediates its conversion to RanGDP provide a directional polarity to nucleocytoplasmic transport and nuclear assembly. RanGTP is concentrated in the nucleus because of the chromatin-attachment of its cofactor RCC1. Nuclear RanGTP binds to karyopherins, promoting the disassembly of cargo from karyopherins involved in import (e.g. importins α and β and the attachment of cargo to those involved in export of which the exportin Crm1 (exportin 1) that exports ribosomal subunits and many other cargos is most important) [255]. RanGTP remains bound to karyopherins till they exit the nucleus but is converted to RanGDP in the cytoplasm with the help of the activator protein RanGAP that promotes GTP hydrolysis to GDP; free RanGTP is concentrated in the cytosol because of the cytoplasmic location of RanGAP. In contrast to RanGTP, RanGDP promotes the attachment of cytoplasmic proteins to karyopherins for import into the nucleus, but it does not accompany them. Thus nucleocytoplasmic transport continually depletes the nucleus of Ran; it is replenished by transport to the nucleus of RanGDP bound not to karyopherin but to a completely unrelated carrier, an NTF2 dimer, which binds to FG-Nups in a different but partially similar way [256,257]. Inside the nucleus, RCC1 charges Ran with GTP, dissociating it from NTF2, which is transported back into the cytosol. The complementary distribution of the two forms of Ran [258] must have been a pervasive spatial backcloth against which the nucleocytoplasmic exchange machinery evolved [259].

Its importance, however, goes way beyond the origin of the nucleus, because RanGTP is intimately involved in spindle assembly and mitosis through promoting kinetochore and centrosome functions. If as I have repeatedly argued the origin of mitosis was so crucial to the survival of the earliest prekaryote cells that its main features must have evolved before the nucleus [3,4], it is reasonable to suppose that the initial function of the Ran system was for mitosis [260], thus preadapting the prekaryote for the subsequent and probably immediately following origin of the nucleus. RanGTP promotes the assembly of microtubules at the kinetochores, and its binding to chromatin may have originally evolved for this reason. Its putatively pre-existing chromatin location explains how the assembly of the envelope (itself under RanGTPase control) was spatially organized around chromatin from the outset. If the basic features of the RanGTP/GDP cycle existed prior to the origin of the nucleus, it both shaped and facilitated the latter. As proposed by Jékely [260], RanGTPase probably originated for its mitotic functions by gene duplication and divergence from Rabs. Prior to the origin of the nuclear envelope I suggest RanGTPase was already associated with chromatin by its RCC1 exchange factor that is essential for the conversion of RanGTP to RanGDP (the cytosolic form). Thus the RanGTP/GDP cycle and the chromatin attachment of RanGTP via RCC1 probably evolved for the control of mitosis including its spatial aspects in the prekaryote. Interestingly RCC1 is another β-propeller protein with 7 WD-40 domain blades like so many other such proteins (e.g. trimeric G proteins, the vesicular traffic protein Sec13) that proliferated during eukaryogenesis. Thus even before the origin of NPCs the cell was spatially polarised into Ran-GTP-rich chromatin and RanGTP-rich cytosol [259]. RanGTPase and a suite of Ran binding proteins played key roles in both phases of NPC evolution.

### Phase one: from coated vesicles to nuclear pore complex

The key step thereafter for the origin of the nuclear envelope, I propose, involved duplication and modification of some COPII component(s) that allowed cisternal fusion without prior total separation of vesicles from the donor membrane and therefore necessarily without uncoating (Fig. 5c left). Thus the essential key innovation for making the nucleus was a modification of COPII coats. As the cisternae fused together, the COPII-derived coatomers would remain on the membrane (Fig. 5c centre) and automatically become clumped as cylindrical aggregates, shown in surface view in Fig. 5c. Thus, a single mechanistic innovation in the known precursor would at a stroke evolve both a fenestrated nuclear envelope and NPC scaffolds allowing passive nucleocytoplasmic exchange. Cytoplasmic motors actively moving COPII, and later other, secretory vesicles would be prevented from causing DNA damage and interference with transcription, by getting entangled with DNA and nascent RNA, especially pre-rRNA, which being so long (comprising 3 molecules before cleavage) would be especially susceptible to shearing. Homotypic fusion of ER-derived COPII vesicles occurs in modern cells to generate the preGolgi intermediate compartment [261,262]; allowing it to go ahead without prior uncoating would generate a plausible precursor of the nuclear envelope (including its pore complex) in a single step, provided that cisternae already attached to chromatin (or COPII vesicles containing chromatin-attachment proteins) were also involved in that fusion.

Thus, avoiding DNA and RNA breakage was the primary advantage of the NE and NPC, which initially was only narrow enough to exclude secretory vesicles but not ribosomes and polymerases (Fig. 5a). Avoiding DNA breakage has been criticised as a selective force, by the claim that there is no problem '...during mitosis, even in species where they are permanently uncondensed, despite the fact that the nuclear envelope disintegrates at the beginning of mitosis...' [122]. This objection is misleading and invalid. The supposed counter-example actually indirectly supports my thesis, because the only eukaryotes in which histones have been lost and DNA is truly uncondensed during mitosis are peridinean dinoflagellates in which the mitotic spindle is entirely outside the nucleus and the envelope never breaks down [263-265]. Their perpetually relatively condensed but histone-free chromosomes [266] probably evolved secondarily only after, almost uniquely, spindle and chromosome separation by evolving intra-NE kinetochores bypassed the shearing problem by a different route [267]. However, avoiding transcript breakage, a novel proposition, was perhaps an even stronger force, not only for the origin of the NE but also of the gel-like interphase nuclear matrix [163], in which DNA topoisomerase plays a key role to relieve positive supercoiling ahead of transcription complexes.

Thus, we have both a plausible physical mechanism based on known cell biology and a plausible selective advantage for the origin of the nuclear envelope. As the envelope-associated part of the NPC involved over a dozen proteins of the scaffold Nups have α-solenoid and/or β-propeller domains prior to the eukaryote cenancestor, there must have been a rapid multiplication of these following the minimal change in just one or two needed to set the process in motion. In so far as some of these core scaffold proteins are relocated to kinetochores and/or centrosomes during mitosis, it is possible that some of these duplications actually preceded the origin of the core complex and were selected initially for their functions in mitosis rather than in pore complexes. However, fruitful speculation along these lines in impeded by our knowledge of the detailed cell biology of the mitotic behaviour and possible mitotic functions of NPC components being primitive and largely restricted to one eukaryote lineage only (opisthokonts, which include animals and fungi), almost nothing being known for bikonts, which have an even greater range of mitotic behavioural diversity than opisthokonts. It is especially important to study these processes in Euglenozoa as features currently assumed to be universal for eukaryotes might only characterise neokaryotes. A related problem is that even within opisthokonts NPC behaviour differs substantially among organisms with closed mitosis (e.g. yeasts), open mitosis (animals), or semi-open mitosis (e.g. *Aspergillus*); without comparable knowledge for a good range of bikonts inferring the ancestral state even for opisthokonts, still less for eukaryotes, is hazardous. A key recent discovery is that a major core complex of about 10 scaffold Nups (the Nup107-160 complex in animals; different names in fungi) behaves as a unit during mitosis. This complex probably includes proteins with membrane curving functions likely to have been central to the origin of nuclear pores, but present knowledge also does not allow one to deduce which components would minimally have been necessary for the first phase of NPC evolution and which might have been added later in the second phase, dominated by the addition of a completely different class of Nups, the FG Nups and their interactions with karyopherins.

### Compartmentation and its consequences: Ran-GTPase, FG-Nups and karyopherins

Karyopherins, which mediate protein import/export and the FG-rich Nups with which they interact to allow nucleocytoplasmic exchanges are both large eukaryotic gene families with extremely few bacterial homologues that became highly diversified during eukaryogenesis prior to the eukaryotic cenancestor [14,17]. The fact that karyopherins use basic amino acids as nuclear localisation signals (NLS) arguably stems from their major early cargo having been very basic histones and other DNA binding proteins [3] as well as basic proteins that bind RNA, especially ribosomal proteins [259]; early cargo probably included inner-membrane DNA-binding proteins [19], that would otherwise be impeded by the novel integral membrane Nups, unless from the start NPCs opened to let them cross the NPC NE domain, and histones. Ribosomal assembly became localised to the nucleolus probably because transcription of such long molecules is so slow that it is optimal to start assembling proteins onto them and process them by RNA cleavage, and base modification; the neomuran ancestor already had numerous RNPs and enzymes on a fibrillarin matrix associated with transcription sites [3]. Evolution normally takes the line of least resistance and changes as little as possible; stabilising selection prevents capricious 'redesign' so there was no chance of relocating ribosomal assembly to the cytoplasmic point of use. tRNAs are exported by attachment to karyopherins by proteins with NLSs, but completely separate machinery evolved for exporting ribosomal subunits and another for mRNA [17], indicating that their localisation was probably perfected in parallel not serially. Messenger RNA capping and polyadenylation probably arose primarily to prevent RNAase degradation during the much longer time it would take for mRNA to reach ribosomes than in bacteria, mitochondria and plastids, where they can attach during transcription [70]. But both probably became markers of maturity and readiness for export by the novel mRNA export machinery that arose by duplication of a bacterial GTPase to make Nug1p/Nug2p [17]. The ribosome-subunit export machinery recruited a duplicated neomuran AAA+ ATPase as Rix7P [17], possibly in the ancestral neokaryote, not the first eukaryote.

As previously argued [3,5] a phase of passive restriction of molecular exchange between the nucleoplasm must be postulated prior to active and selective import and export. Recently the likely intermediate stages have been modelled by Jékely [259], demonstrating by diffusion-reaction simulation that partial enclosure of chromatin by membranes with quite large apertures can lead to marked differences in molecular concentrations, which makes such intermediates selectively plausible; furthermore he showed that, even without any membrane boundary, direct or indirect binding of molecules to chromatin can change local concentrations of diffusible molecules. A limitation of such spatial modelling is that we do not know the size of the cell in which the nuclear envelope originated. Even though it almost certainly greatly increased in size compared with its bacterial ancestor [4] during the origin of the endoskeleton and phagocytosis, it could have been much smaller than the 20 μm he assumed, which would have made it harder to develop significant concentration gradients in the pre-envelope phase than in his model and thus increase the relative selective advantage of using a membrane and NPCs for effective compartmentation over simple binding to chromatin. The modelling has heuristic value but is notably oversimplified for the final stage with NPCs as it ignores the fact that RanGTP is exported and RanGDP imported on carriers (different ones) and do not diffuse freely.

Karyopherins have three distinct domains: an N-terminal Ran-binding loop, a central nucleoporin (Nup)-binding domain, and a C-terminal cargo-binding motif that interacts with NLS (in the case of importins) or NES (in the case of exportins). Their central region that interacts with Nups consists of HEAT repeats (comprising pairs of antiparallel α-helices) that can bind the FG repeats of FG nucleoporins. The great diversity of karyopherins and FG Nups must have arisen during eukaryogenesis from a common ancestor by repeated gene duplications, probably with some domain shuffling. As karyopherins have other functions independent of the nuclear pore complex it is likely that one of these was the ancestral one. Deciding what that was is not easy, partly because many of these functions are still poorly understood. The karyopherin Crm-1 is a member of the importin-β superfamily of transport receptors [268] that in addition to nuclear export is also needed for the intranuclear maturation and possibly export of the small ribosomal subunit. Crm-1 recognizes a leucine-rich NES [269]. This specificity might stem from the fact that RanGAP might have been its initial cargo (whether first to exclude it from the nucleus or even earlier for mitotic functions). It was recently discovered that Crm-1 has roles in mitosis (at least in opisthokonts) as it mediates the recruitment of the RanGAP1/RanBP2 complex to kinetochores and maintains kinetochore-fibre integrity [270]. Crm-1 is also involved in centrosome duplication in animals and interacts strongly with the centrosome (spindle pole body) of fungi [271].

Domain shuffling or quantum divergence was involved in early karyopherin diversification since Crm-1 has its own special N-terminal domain that differs from the globular N-terminal domain of most importins that is a mixture of HEAT and ARM (armadillo, a related type of repeat) repeats. Likewise the tRNA exporter (exportin-t) has a unique C-terminal domain that presumably recognises tRNA cargo. Once Crm-1 became able to export its first cargo, whatever that was, by binding to leucine-rich NESs, additional cargos could be added to its repertoire by adding such residues to pre-existing proteins or by evolving binding of adaptor proteins already bearing them to cargo (notably various RNAs) that lack them, allowing them to piggy back on NES bearers.

Many importins act as cytosolic chaperones and bind strongly to basic proteins such as histone H1 and some ribosomal proteins, which would stop them binding to or interfering with cytoplasmic RNA, e.g. mRNA [272]. This would have been useful even before the origin of the nucleus. As Jékely [259] pointed out, that might have been their primary function before they were recruited as karyopherins. Such acquisition of additional functions by pre-existing molecules reduces the difficulty of evolving all components of the complex transport machinery simultaneously. Though the basic idea that some elements of the transport machinery had evolved before Nups and the envelope is attractive, as is the idea that chaperones of various sorts were probably important before compartmentation, I am unconvinced by his specific suggestion that the primary function was for binding to ribosomal proteins [259]. This is partly because different importins are used for different ribosomal proteins, which suggests secondary recruitment of variants after a primary karyopherin function was established, partly because some of these bind other basic proteins (e.g. the importin β-importin7 heterodimer binds rpL4, rpL6 and histone H1 [272]) and I do not see how one can say that ribosomal protein chaperoning was primary, partly because before the nuclear envelope evolved ribosomal proteins could have bound rather quickly to nascent rRNA, which would have been available in large amounts in the same compartment in a fairly small cell, and partly because I prefer the idea that the first nucleoporin was directly associated with loading proteins, e.g. RanGTP or histones directly onto chromatin, as Crm-1 does today for mitosis.

One class of chaperones that likely predated the nuclear envelope is histone chaperones. The two multimers from which the core nucleosome octomers are made (H3/4 and H2a/H2b) each have different chaperones (e.g. CaAF-1, Asf-1 for H3/4 or for H2a/H2b), some of which associate with them prior to import. Though they are imported as macromolecular complexes it is the core histones themselves that bear the NLS (several that can interact with several different karyopherins; there are always many different karyopherins; 15 in yeast). Core histones are imported by a monomeric importin β, in contrast to many proteins that use a more complex heterodimer in which importin α acts as an adaptor between NLS and importin β (including histone H1: [273]). I suggest that this simpler system, which is also used for most ribosomal proteins may be the ancestral one and that the classical one involving also importin α and more complex NLS is derived. Once a primitive import system evolved importin gene duplications would generate many different importins and numerous molecules that unlike histones did not have NLS as part of their intrinsic structure could have added them.

One such clear case of addition of NLS is the export adapter Nmd3; its C-terminal domain has both NLS for entering the nucleus and nuclear export signals (NES) for exiting it so it can shuttle between nucleus and cytoplasm. Inside the nucleus it binds to newly made 60S ribosome subunits and carries them out with it [274]. As the archaebacterial homologue of Nmd3 lacks this C-terminal domain it was clearly added during eukaryogenesis to allow such shuttling and ribosome export. The most important and perhaps original export receptor is Crm-1, the karyopherin that mediates export of the Nmd3-60S-ribosome complex among many others, e.g. the signal recognition particle, spliceosomal snRNAs, 5S rRNA, but not most mRNAs. Obviously karyopherins themselves have to be exported. Most exit the nucleus as they enter it under their own steam. But importin α piggy backs on importin β on the way in and on karyopherin 109 on the way out, which might be interpreted as evidence that it evolved after those two similarly ubiquitous types.

The ancestral karyopherin may have arisen by gene duplication from the adaptins that attach clathrin coats to transport vesicles as they are structurally similar with HEAT repeats [17]; if the suggestion that the closest bacterial relatives of these proteins are certain phycobilisome proteins of cyanobacteria [17] stood up to more thorough study, this could be the first example of lateral gene transfer from cyanobacterial food of the prekaryote predator being important in early eukaryote evolution. That NPCs and Sec13 are not the only nuclear pore associated proteins with affinities with coated vesicle proteins strongly supports the view that the origin of a primitive endomembrane system with various types of coated vesicles preceded and was more fundamental to eukaryogenesis than the origin of the nucleus itself [4,5,11,70,123,163].

The difficulty of deciding on the likely ancestral functions of such pervasive molecules as RanGTP and karyopherins is highlighted by the fact that in animals at least, importins α and β are both involved in spindle and nuclear envelope assembly; to be consistent both with my thesis that some mitotic functions preceded NPC-related nucleocytoplasmic exchange functions and that importins α was not part of the ancestral karyopherin importer one would have to suppose that the mitotic roles of importin α are secondary (i.e. evolved after NPCs), which current evidence cannot exclude. An analogous problem concerns the fact that RanGDP is imported into the nucleus not by karyopherins but by its own custom importer, NTF2. The suggestion that this may be because Ran import evolved before karyopherins [17] is implausible for two reasons. Such a small molecule would be the least likely to be excluded by a primitive FG Nup plug and could  hardly have provided the first impetus for the evolution of active import. Secondly, if it did evolve first, why did all the other things that are imported by karyopherins not adopt that carrier. It seems to me that the diversity of karyopherins argues that they came first and were co-opted for the transport of any molecule that could acquire NLSs or could be bound by available or readily evolvable NLS-bearing adaptors to karyopherins. Perhaps the RanGDP is so small that it could not acquire a basic NLS without disrupting its function. If it did acquire an NLS this would also be present on RanGTP, which is exported (by binding to karyopherins), so its addition would contradict the need to export the GTP-bound form and import the GDP-bound one of the very same protein. It may have been easier to adopt a different carrier that would circumvent that contradiction. Its novel acidic C-terminal tail that is recognized by NTF2 was probably co-opted as the import signal because it is distinctive and absent from other Rab/Ras family proteins and thus would not lead to their accidental import into the nucleus. The suggestion that NTF2 which is essential for nuclear envelope function was acquired from the proteobacterial premitochondrial symbiont because homologues exist widely in eubacteria but not archaebacteria is fallacious, because of the likelihood that this was one of many eubacterial proteins that were lost by the ancestral archaebacterium after it diverged from its neomuran sister from which eukaryotes evolved. Such proteins are widely present in both actinobacterial and endobacterial Posibacteria so would have been present in the ancestral neomuran. Thus the phylogenetic history of this and other nuclear-envelope related proteins present widely in eubacteria but not archaebacteria do not constitute evidence that the nucleus evolved after mitochondria, a fallacious conclusion stemming from considering archaebacteria to be ancestral to eukaryotes rather than their sisters, a phylogenetic mistake which prevents many from accepting that Posibacteria and with their typically eubacterial genes were ancestral to both archaebacteria and eukaryotes.

Although there is some redundancy among karyopherins [275] some are essential for a small subset of molecules. Thus import of histone H1 is much more complex than for core histones, and some ribosomal proteins require different machinery from the majority. This complexity suggests that many molecules that were not initially imported were added later to the import repertoire in a piecemeal fashion by acquiring a diversity of NLS recognized preferentially by different karyopherins. Some ribosomal proteins show clear evidence of the later addition of NLS in expansion segments [276], an example of the quantum evolutionary impact on ribosomes of the origin of the nuclear envelope earlier postulated [3,70]. Though one might think that replication and repair enzymes and RNA polymerases were among the early molecules imported [3], it remains unclear how many of these, especially RNA polymerase are imported [277]; the case of transcription initiation factors is interesting in emphasizing that in a large macromolecular complex only some (minimally one) constituents needs an NLS - thus TAF10 a component of transcription factor complex TFII (that recognizes TATA boxes) and other transcription regulatory complexes lacks an NLS but is imported by being bound to three proteins with histone fold domains that contain NLS [278]; these histone-fold factors might themselves have evolved from core histones and thus ancestrally would have had NLSs.

Transport of multiprotein complexes is a major way in which evolution can add extra proteins to the transport repertoire without adding NLS to each; transport of ribonucleoprotein complexes in which at least one protein had an NES was key to the export of RNAs which themselves could not mutationally acquire NESs. Because NLSs and NESs are rather generalized, many proteins would by chance have had weak transport signals that could be improved by mutation, without having to add or insert an extra NLS domain. However the generalized nature of the signals also means that numerous proteins that do not need to be transported could by chance confuse the system so there was probably also selection against such resemblances becoming too strong. There is evidence from in vivo studies that such confusion is a real factor [275], emphasizing that evolution has, unsurprisingly, not been able to evolve perfect discrimination. Though I have speculated as to what may have been imported or exported early or initially, one must emphasize that selection for compartmentation would probably not act separately on single molecular types but be a bulk selection for numerous simultaneous mutations in different genes that collectively yielded economies in protein synthesis or accelerated growth.

Origin of the NE would have complicated sex, requiring nuclear fusion before meiosis, but appropriate timing of Ran GTPase fusion control probably fixed that. Because selfish DNA, especially transposons and endogenous retroviruses, spreads much more slowly in clonal than in sexual populations [279,280], a sexual protoeukaryote would have suddenly been inundated by such genetic parasites [4]. One response was to evolve posttranscriptional gene silencing to destroy double-stranded RNA.

### Nuclear envelope structural proteins and cytoplasmic organelle attachment proteins

**Figure 6:**
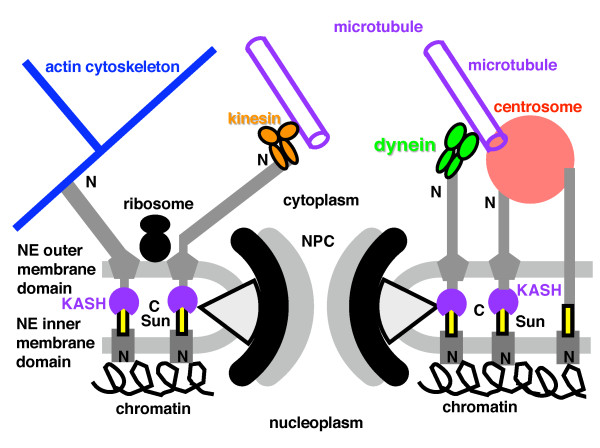
**Role of Sun-domain and KASH-domain proteins in nuclear envelope architecture**. Sun-domain proteins embedded in the inner membrane attach it directly to the DNA surface of the peripheral heterochromatin (the nucleoskeleton). Their Sun-domains (yellow) bind to the KASH domains (purple) of proteins embedded in the outer membrane, which attach it to the cytoplasmic actin filaments, microtubules, and centrosome of the cytoskeleton. Grey pentagons represent the membrane spanning domain(s) of the KASH-domain proteins and grey rods their flexible cytoskeleton-binding N-terminal domains, which differ greatly among the various types. Microtubules may be attached to KASH-domain proteins either by kinesin or dynein motors. The firm Sun-KASH linker complex (known as LINC) within the perinuclear cisterna holds the inner and outer membrane domains of the NE together with the correct spacing and transmits mechanical forces from cytoskeleton to nucleoskeleton or vice versa without damaging it. Some eukaryotes, e.g. animals, lobose amoebae and peridinean dinoflagellates, (probably polyphyletically) evolved a proteinaceous lamina beneath the inner membrane to further strengthen the nuclear periphery, but this was probably absent in the first eukaryotes; additionally to the universal interactions shown, in animals only cytoplasmic intermediate filaments (IF) attach to KASH proteins via plectin adaptors and lamin IF proteins associate with the intranuclear domain of Sun proteins. For simplicity the fact that Sun-domain proteins are homodimers with a coiled coil domain between their two membrane-spanning and chromatin binding domains (lumped here as grey rectangles) and two Sun domains is not depicted.

Numerous new NE structural protein and organelle attachment proteins evolved during eukaryogenesis, but little is known about them in most organisms and still less about their evolution. Of special importance for NE structure and attachments of other organelles to the nucleus are two families of NE membrane proteins: those with Sun or KASH domains [281,282]. These two protein families are jointly responsible for holding together the two (topologically continuous) NE membranes and for attaching them to the cytoskeleton and the nucleoskeleton. As Figure 6 shows, Sun-domain protein dimers are embedded in the inner membrane by membrane spanning domain(s) near their N termini with their C-terminal Sun domain protruding into the lumen of the perinuclear cisterna. Their Sun domains bind firmly to the C-terminal KASH domains of a variety of proteins similarly embedded in the outer membrane of the envelope, forming a linker complex holding the two membrane domains close together [281]. The N-termini of the Sun-domain proteins binds firmly to the DNA of the peripheral heterochromatin, whereas the N-termini of the KASH proteins are much more variable, binding to the actin cytoskeleton as well as via the molecular motors dynein or kinesin to microtubules [283]. Centrosomes are also attached to the nucleus by either KASH proteins (animals [284]) or by a Sun protein (Sun-1 in *Dictyostelium *[285]). The Sun/KASH linker complex forms a mechanically robust bridge between the inner and outer membranes of the NE that can firmly bind the nucleus to other organelles or transmit forces from outside motors (as in nuclear migration in animals [286]). In meiosis cytoplasmic microtubule motors also move telomeres together, creating the bouquet stage of prophase to help homologous chromosomes pair; Sun-KASH linkers across the NE mediate this movement [287,288]. In fission yeast Sun-KASH and other NE proteins link intranuclear centromeres through the envelope to cytoplasmic microtubules [289]. I propose that the origin of the Sun/KASH protein linkers was a key step in the original attachment of protoER membranes to heterochromatin during the formation of the nucleus.

Earlier I postulated that lamins were ancestrally involved at this attachment [4,5,18,163] but this is now unlikely because, like other intermediate filament family proteins, they are restricted to animals and known for sure only in animals with guts and nervous systems (Cnidaria and above). Sun proteins are much better candidates for a central role in NE origins because they are evolutionarily more widespread and because of their key role in ensuring nuclear integrity and attachment to other cell structures. The Sun domain is related to the discoidin domain; this broader domain family is present in all eukaryotes and even in some bacteria such as actinobacteria [17] so would have been present in the ancestral neomuran ancestor. The Sun-KASH interaction has only been studied experimentally and the locations of the proteins verified in unikonts, but Sun proteins have been detected through sequence homology across the neozoa [17,290]. When I studied Sun-1 and Sun-2 in *Dictyostelium *by BLASTP I could detect homologues for Sun-1 also only in neozoa, but using Sun-2 found plausible homologues with conserved Sun domains in three phyla of Eozoa including Euglenozoa, but none in bacteria. KASH proteins are more diverse in domain structure length and sequence, making such simple comparisons more difficult. Nonetheless, it is likely that Sun-domain inner membrane proteins evolved at a very early stage in the origin of the nuclear envelope to attach endomembranes directly to chromatin; that is a simpler origin for chromatin membrane attachment than one mediated by proteins like lamins that are not themselves inner membrane proteins. KASH proteins would probably have evolved almost immediately thereafter to stabilise the envelope mechanically.

Although animal Sun-domain proteins bind to lamins as well as to DNA or chromatin this is probably not their primary mode of targeting to the nuclear periphery as localization there is not prevented by the absence of lamins; some have known DNA-binding motifs. In *Dictyostelium *Sun-1 binds directly to DNA and also to a protein linker to cytoplasmic centrosomes, apparently independently of the characterized KASH protein. At least two different Sun-domain inner membrane proteins had evolved by the ancestral neozoan [290]. I suggest that a nuclear lamina composed of lamins and the intermediate filament family as a whole may have evolved in the ancestral animal (sponge) to increase the mechanical strength of the giant oocyte nucleus when the ancestor of animals evolved oogamy (giant eggs and tiny sperm) and that the family diversified into various cytoplasmic filaments when epithelia evolved in the first sponge to give added mechanical strength to them. Different paralogues were probably selected early for somatic cells and oocytes. The mechanical robustness of the lamina may have made it essential for it to be reversibly disassembled at mitosis, which may have been simplest to achieve through fragmentation of the whole envelope; thus open mitosis [291] probably evolved simultaneously with the lamina in the ancestral animal. Choanoflagellates, the closest outgroup to animals, have semi-open mitosis [292], making it likely that the direct unicellular ancestor of animals (probably a stem choanoflagellate [293]) also did.

A proteinaceous nuclear lamina evolved independently in amoebae of the amoebozoan subphylum Lobosa (e.g. *Amoeba*, *Hartmannella*), where in the giant amoebae it is far thicker and more complex than in animals [294]. Their ancestor lost cilia, centrioles and all cytoplasmic microtubules so as to focus on locomotion by actomyosin; a proteinaceous lamina was probably especially important to protect the nucleus from shearing forces which must be far greater in such amoebae that undergo repeated sol-gel transformations compared with the ancestral zooflagellate eukaryotic cells where the cytoplasm is almost permanent semi-rigid actin gel, except during cytokinesis by cell cleavage. Having evolved such a robust lamina lobose amoebae could dispense with the peripheral chromatin that underlies the NE in the zooflagellates most closely related to them (choanoflagellates and Apusozoa). Apusozoa (apusomonads and planomonads) are either sister to opisthokonts (animals, Choanozoa, Fungi) or the paraphyletic ancestors of both opisthokonts and Amoebozoa and have particularly strongly developed peripheral and internal heterochromatin in their nuclei, so must have a higher DNA content per unit nuclear volume than choanoflagellates. By contrast lobose amoebae have almost no heterochromatin visible in interphase, so their DNA per unit nuclear volume must be rather low (which goes some way to explaining why getting enough DNA for even simple PCR and sequencing from sparse cultures is often much harder for Lobosa [295] than other eukaryotes). Another group that often has giant nuclei and a proteinaceous lamina are the dinoflagellates. This coupled with the evolution of an extranuclear spindle with kinetochores embedded in the nuclear envelope probably allowed peridinean dinoflagellates to dispense entirely with heterochromatin as a nuclear skeleton and even largely also with histones, which are absent from the majority of their DNA [296] which is neutralized by divalent cations instead [297] and sparse bacterial-type DNA-binding proteins [298,299]; only one standard histone is known, perhaps involved in double-strand break repair [300]; the loss of histones entailed radically altered transcription factors that bind to TTTT instead of TATA boxes, but dinoflagellates retain standard telomeres associated with the nuclear envelope [301] and relatively normal spliceosomal intron splicing [302].

The origin of lamins and open mitosis in animals, a convergent kind of lamina and open mitosis in Lobosa, and of the dinoflagellate closed mitosis with extranuclear spindle, are all mentioned as examples of secondary changes from the ancestral state that must not be allowed to confuse us in reconstructing the first eukaryote. The peculiar mitosis of dinoflagellates in which kinetochores are embedded in the NE was once proposed as a model for an early version of mitosis before microtubules evolved, but that idea became untenable when it was discovered that they do have microtubules; I argued long ago that it was irrelevant to the origin of eukaryotes because dinoflagellates are so complex that they cannot be primitive and must be very advanced higher eukaryotes [70], which is demonstrated beyond question by their branching within the alveolates and these within the kingdom Chromista that was formed by the secondary symbiogenetic enslavement of a red alga (Fig. 1) [9,61,86,87,303]. Despite this irrelevance, dinoflagellate mitosis is oddly still sometimes mentioned as being germane to the origin of meiosis because telomeres are similarly moved on the NE [290]; though the movement of dinoflagellate kinetochores may well use the same cytoplasmic machinery (microtubules and dynein) as do meiotic pre-bouquet telomeres, and it is likely that the attachment of both depends on Sun-domain inner membrane, it is most unlikely that the ancillary attachment proteins for telomeres (e.g. Bqt1-4 [304,305]) are related to those of dinoflagellate NE plaques. How peridinean dinoflagellate mitosis may have evolved from the more normal patterns found in other alveolates is discussed in [267]. Independently of dinoflagellates, metamonad Parabasalia evolved NE-envelope embedded centromeres and extranuclear spindle, but they are equally irrelevant to the origin of mitosis or meiosis despite sometimes being mentioned in that context. Nonetheless, molecular studies of NE-embedded peridinean and parabasalian centromeres would be very revealing for cell biology and evolution.

The ratio of heterochromatin to euchromatin mass affects the quantitative relationships between genome size and nuclear and cell size [18]. However the reasons for its variation even in protists are poorly understood. I originally assumed that if a nuclear lamina was universally present euchromatin and heterochromatin ought to be able to serve equally well as a nuclear skeleton and nuclear volume determinant [169]. On that view it ought to be more economical of DNA and protein to use the less dense euchromatin for this function rather than the denser heterochromatin as less material would be needed for the same volume. I assumed that the presence of heterochromatin in so many animal and plant somatic cells mainly reflected the fact that their nuclear volumes varied so greatly that heterochromatin was only unwound fully to make dispersed chromatin in the largest cells such as giant neurons, whose large nuclei lack condensed chromatin [169,170,176]. Thus multicellular organisms would have a complete range of chromatin unfolding and nuclear volume from such giants to the most compact nuclei of sperm and red blood cell nuclei where all is maximally condensed and transcriptionally inactive. I argued that protists with multiple fission life cycles with alternation between giant cells with huge nuclei and tiny ones with compact ones should have heterochromatin, but that those with simple binary fission cycles should not, and cited much comparative supportive evidence [306]. However, as recently explained there are now several clear counter-examples to that thesis, which must therefore have been oversimplified [18]. Several examples of ordinary binary fission cell cycles without major changes in nuclear volume beyond the normal two-fold per cycle but with extensive heterochromatin, especially on the nuclear periphery, but also interstitially are now apparent. This include cryptomonads and haptophytes (interestingly now grouped together in the chromist subkingdom Hacrobia, for which extensive heterochromatin was arguably the ancestral state); Apusozoa [307,308]; choanoflagellates (probably with a lower heterochromatin/euchromatin ratio); and euglenoids. In all these cases the whole group possesses this character. It is noteworthy that in other whole groups, e.g. lobose amoebae, Excavata, and in euglenozoan Kinetoplastea and Diplonemea condensed chromatin is largely absent or relatively sparse in interphase. Thus there is strong phylogenetic constraint; for many protist groups changes from a heterochromatin-rich to heterochromatin-sparse nuclei or the converse are only made relatively rarely in protist evolution.

One way to rationalize this is to suggest that the ancestral eukaryote was a middle-sized cell in which large amounts of heterochromatin were the primary nuclear skeleton on which the nuclear envelope was assembled and cryptomonads, haptophytes, and euglenoids, for example, have retained that heterochromatin-rich state but that the protist groups with more dispersed chromatin hit upon more economical ways of making or using their nuclear skeleton or ones more suited to their individual life styles. I pointed out above that the evolution of giant nuclei in lobose amoebae and dinoflagellates was associated with cytological novelties that arguably allowed them to dispense with heterochromatin as a peripheral nuclear skeleton (the nuclear lamina of Lobosa and the permanently condensed histone-depleted chromosomes of Peridinea) and depart radically from the ancestral eukaryote condition. I now suggest that evolving especially small cells and nuclei coupled with a relatively rigid cytoplasm may also have allowed the loss of most peripheral heterochromatin (and associated metabolic and spatial economy) without mechanically harmful consequences, and a big increase in the ratio of coding to non-coding DNA. This may be why phylogenetically diverse organisms converged on a largely heterochromatin free-state (free-living budding yeasts, intracellular Microsporidia, and above all the nucleomorphs of cryptomonads and chlorarachneans (relict enslaved nuclei)) or on a sparse heterochromatin state (the intracellular parasitic coccidian Sporozoa, like the malaria parasite *Plasmodium*) and more gene-rich genomes than most eukaryotes. Somewhat intermediate relatively small-celled organisms like choanoflagellates, Filasterea or bodonids with some peripheral heterochromatin but less than Apusozoa or cryptomonads may have made some minor economy but with less radical change. In protists there are numerous secondary adaptations affecting the nuclear envelope, for example the huge macronuclei of ciliates or the large nuclei of actinophryid heliozoa that nucleate axopodia or the huge nuclei of many Retaria (Rhizaria). This proposal fits much comparative data but would require several independent reductions in heterochromatin richness in excavates and within plants and chromists, which is not unreasonable.

I have long thought it an attractive idea that both centromeres and telomeres were ancestrally attached to the nuclear envelope [4,309]. Their heterochromatin could have been a major fraction of the heterochromatin that forms the periphery of nuclei onto which the inner membrane is attached by Sun proteins. The involvement of Sun proteins as the direct binder of chromatin makes it clearer than ever that chromatin itself is really the fundamental nucleoskeleton, as I have repeatedly argued [18,169,170,176,211,309]. A major consequence of the nuclear envelope was the initiation of a new nucleoskeletal function for DNA. Elsewhere I explained how this, coupled with the adaptively significant huge variation in cell volumes of eukaryotes that the origin of the endoskeleton and endomembranes made possible, is the fundamental evolutionary reason why eukaryotic genomes are typically much larger than bacterial genomes, why their size varies so immensely, and why it correlates so well with cell volume but not with organismal complexity [18]. The (nucleo)skeletal DNA explains many aspects of the evolution of eukaryotic genome size where the still more popular selfish DNA/mutation pressure theories fail totally [18,310]. The reason why most of the non-coding DNA in eukaryotic genomes exists is fundamentally structural, which is why it is called skeletal DNA whether it is centromeric, telomeric or interstitial [18]. Parts of it are transcribed and involved in the positional control of centrosomes and heterochromatin, but at base these are structural and not coding functions; the transcription and base-pairing are needed for positional control of structure within the skeletal DNA component of the genome. As adumbrated over 30 years ago, the evolution of eukaryotic genomes are to be understood in terms of the complementary functions of genic and skeletal DNA [169]. As stressed then, the more skeletal DNA a cell has, the larger the habitat available for viruses and transposons to flourish in without harming the cell as much as they would if they if inserted themselves into genic DNA. Therefore one expects larger genomes to have more parasitic genetic elements and for these to be especially concentrated in heterochromatin because this is where they can best evade purifying selection against them.

However, many have confused these well-known correlations of selfish element abundance and location with causation. In my view they are consequences of the deeper cell biological evolutionary forces acting on nuclei, not their fundamental causes [18]. It does not matter functionally whether genomes increase in size by duplication of genes, non-coding DNA, selfish genetic elements or whole genomes - all have been important in the past - because the mutational mechanism of origin of the extra DNA is not the main or fundamental determinant of the actual spectrum of genome sizes in different organisms. To understand these we must understand the adaptive significance of the differing cell volumes in organisms with different life styles and the cell biological principles of intracellular scaling that make an approximately constant ratio of nuclear and cell volume functionally superior [18]. These cell biological considerations did not exist before the origin of the nuclear envelope and are the major reason why the evolution of genomes is so radically different in eukaryotes compared with bacteria [18,211,311]. Purely population genetic perspectives are fundamentally misleading as they fail to appreciate the key role of the major innovations in cell structure during eukaryogenesis in stimulating first a radical change in chromosome structure and then in the selective forces acting on genome size, and sometimes [121] even misrepresent what is known about the radical differences in cell biology between bacteria and eukaryotes. Though mutations in DNA initiated and promoted the changes, from a deeper perspective of cellular constraints affecting the selective forces governing the failure or spread of the causative mutations, cell biology led and DNA and genetics followed, contrary to such widespread misconceptions. Without a cell biological perspective eukaryogenesis is impossible to understand. Nonetheless, the interplay of selection acting on cell properties and that acting on the spread of parasitic genetic elements have both shaped eukaryotic genomes.

The most important evolutionary consequences of selfish genetic elements were probably in the origin of introns [81,312], and the origin of near universal trans-splicing in Euglenozoa [211], though they might also have been involved in the origin of telomeres. But they are not the fundamental reason why eukaryotic genomes are so large and variable.

### Selfish DNA, introns and spliceosomes

Nucleocytoplasmic compartmentation facilitated the origin of spliceosomal introns from group II introns supplied by gene transfer from the enslaved mitochondrion to the nucleus. Only rapidly self-spliced introns in mRNA or rRNA or extremely short easily protein-spliced tRNA introns seem compatible with prokaryote/mitochondrial/plastid organisation; the ready access of ribosomes to mRNA during transcription and the extreme slowing of spliceosomal splicing probably prevented it from ever evolving in prokaryotes [4,5,81]. It could not have evolved until after the second phase of NPC evolution when inner Nups arose to exclude ribosomes entirely and ribosome-subunit export arose (Fig. 5b). Spliceosomal introns could not have evolved in the prekaryote phase, and recent suggestions that the NE evolved to prevent translation of unspliced messengers (latest [21,122]) are illogically back to front. This also cannot possibly have been the initial selective force for the formation of either the nuclear envelope or the initial scaffold part of the NPCs (Fig. 5a) since neither would have excluded ribosomes. This popular theory wrongly endows selection with foresight. By contrast, protection of DNA and nascent rRNA from cytoplasmic-motor shearing damage provides a strong selective force for phase I (Fig. 5a); compartmentation benefits do so for phase II (Fig. 5b). Only then could spliceosomal introns have evolved, as described previously [81]. They are consequences not causes of the NE.

I concur, however, with the suggestion that the selfish origin of spliceosomal introns caused the origin of nonsense-mediated mRNA decay [21]. This mechanism, conserved in unikonts and corticates (all key proteins and both major mechanisms are in *Arabidopsis *[313]), must have evolved prior to the neozoan cenancestor but after the origin of spliceosomal introns. It depends on ribosomes (probably attached to the outer NE) and a test translation recognising stop codons upstream of exon-exon junction sites marked by bound UF3 complexes and stimulating mRNA destruction [314]. A selective force for the origin of this junction-site specificity of UF3 is unimaginable prior to the origin of a high density of spliceosomal introns in genes and inevitable mis-splicing yielding harmful stop codons; though once evolved it could be used, as it now is in plants and fungi, also to destroy intronless similarly flawed mRNA of many intronless genes, which inevitably have sites mimicking exon junctions sequences. Thus like splicing itself, and many genomic oddities like RNA editing [211], nonsense-mediated decay is a consequence of selfish DNA evolution; correcting the bad effects of mutation pressure, not positively adaptive. Intriguingly, the cleavage enzyme itself probably evolved from one used by selfish bacterial plasmids to kill host cells losing the plasmid by mis-segregation [315]. When it originated is unclear, as it has not been demonstrated to occur in any Eozoa. If it evolved after the origin of the nucleus as is possible it could not have influenced its origin. However, the RNA helicase UPF-1 that plays a central role clearly originated in the ancestral eukaryote as it is present in excavates and Euglenozoa, as is UPF-2 with which it binds in the nonsense-mediated mRNA decay but as this helicase has multiple functions this does not prove that nonsense-mediated mRNA decay itself arose that early. Nonsense mediated decay is triggered by failure to remove the exon junction complex (EJC) from intron-containing RNA; two of the four EJC proteins are present in trypanosomatids [316], presumably involved in checking trans-splicing (as introns are extremely rare in trypanosomes [317]), so these at least are also ancient.

### Origins of nuclear protein modification by sumoylation

Sumoylation is a universal eukaryote-specific process mediated by Small Ubiquitin-like MOdifier proteins (SUMO for short) that mainly modifies nuclear proteins [318,319]. It probably evolved at the same time as ubiquitination of proteasome-digested proteins very early in eukaryogenesis, and ought to have been included in Table 1 of reference [27], where I omitted to discuss its origin and importance for eukaryotes. Sumoylation is vital for heterochromatinization, maintaining the stability of eukaryotic chromosomes, for the nucleocytoplasmic exchange via the nuclear pore complex, as well as for tubulin assembly [320-323] and is thus crucial for many non-mitochondrial ('host') eukaryotic properties. Animal, fungal and plant SUMO machinery is strongly associated with the nuclear pore complex [324] and essential for viability [325] and thus was present, and probably essential for pore complex function, in the last common ancestor of neokaryotes. SUMO is also present and strongly conserved in structure in Euglenozoa [326] so evolved in the ancestral eukaryote, though little is known of its functions and ancillary proteins in Eozoa.

SUMO proteins belong to the ancient and diverse ubiquitin superfamily; ubiquitin and SUMO have similar 3-D structures and probably diverged from a common ancestral protein. Moreover, enzymes E1 and E2 that mediate ubiquitination are related to the two that add SUMO to proteins. Both ubiquitin ligase proteins probably originated during eukaryogenesis when the ubiquitin superfamily expanded massively by repeated gene duplication [327,328]. Though neither ubiquitin nor SUMO occur in prokaryotes, Iyer et al. [329] discovered ubiquitin-related genes in a scattered array of eubacteria (never archaebacteria) clustered in an operon with E1- and E2-related protein genes and also a gene for a JAB-domain protein related to the JAB-domain deubiquitinating and desumoylating enzymes. They reasonably suggest that these four proteins represent an ancestral protein modifying system that was widespread in eubacteria before eukaryotes evolved. As such operons are not known from either actinobacteria or α-proteobacteria, they could have entered early eukaryotes by lateral gene transfer from incompletely digested prey DNA. Their scattered distribution within eubacteria is suggestive of lateral gene transfer among them, so the possibility also exists of lateral transfer to the specific actinobacterial lineage that was ancestral to eukaryotes prior to eukaryogenesis.

A curious twist to the origin of this eukaryotic protein modification machinery is the unexpected discovery of an analogous system in actinobacteria, in which the small protein Pup is covalently attached to selected proteins destined for degradation by the actinobacterial proteasomes [330]. Though Pup was called ubiquitin-like by its discoverers [330], Iyer et al. [331] convincingly show it to have a different 3-D structure. It is thus unrelated, having evolved instead from a family of enzymes containing the β-grasp domain; they also showed that the putative Pup ligase (PafA) is unrelated to the ubiquitinating enzymes E1 and E2, and probably works by a different mechanism, being more closely related to glutamine synthetase. Thus the actinobacterial proteasome-related protein modification system was probably replaced by a completely unrelated one during eukaryogenesis. Why? A possible explanation could have been the transitional novel use of proteasomes for digesting externally attached prey, which I recently postulated preceded the evolution of phagocytosis [27]. As I stressed [27], it would be advantageous to digest all prey proteins indiscriminately; thus the actinobacterial restriction of digestion to a few proteins by pupylation would have been disadvantageous and was therefore lost before it could be recruited and modified for novel eukaryotic uses that arguably arose after the early endomembrane system [27]. Therefore, protoeukaryotes evolved a different pre-existing, fully functional ubiquitin-like system based on E1, E2 enzymes and JAG proteins that happened to be available for recruitment to the now more complex 26S proteasome. A 20S core of the proteasome itself, by contrast, was probably directly inherited from the actinobacterial ancestor [12], as was the eukaryotic chaperone PAC2 required for its assembly; Iyer et al. [331] show that a gene encoding a PAC2-related protein that forms a toroidal trimer suitable for nucleating the assembly of the cylindrical proteasome (also present in archaebacteria) is closely linked to PAC2-homologues in actinobacteria, but is absent from more distant species such as the planctomycete *Rhodopirellula *and the δ-proteobacterium *Plesiocystis*, which belong to groups that typically lack a pupylation system (Pup and Pup ligase) [331], suggesting that both obtained it by lateral transfer from actinobacteria.

### Origin of meiosis and sex (syngamy and nuclear fusion)

As discussed in detail elsewhere [3,4,63,332,333], the initial function of meiosis was probably ploidy reduction to correct failures of DNA segregation caused by mitotic or cell cycle control errors that would probably have been greater during eukaryogenesis. As there explained, the key step would have been a duplication of cohesin genes to create separate meiotic cohesins that would be loaded onto chromosomes at the beginning of pre-meiotic S-phase. The changed meiotic cohesins would be digested only from the chromosome arms at meiotic prophase, remaining at the centromere, thus preventing sister chromatid separation until meiosis II. Retention of meiotic centrosomal cohesin in prophase I also arguably prevented the relief of the standard block to further DNA replication initiations imposed at the beginning of each S phase, thereby letting meiosis II continue without a preceding DNA replication, halving the nuclear DNA content to produce haploid cells. Pairing was mediated by DNA hybridization during prophase I, which has a duration proportional to genome size as expected if it is rate-limited by DNA hybridization. As Cleveland first noted [334], the origin of meiosis also requires a novelty in the control of centriolar and centrosomal duplication. In normal mitotic cell cycles centrioles and centrosomes duplicate at the beginning of DNA synthesis S phase (also true of fungal spindle pole bodies, which are simply specialized centrosomes) [71,72,75,76,335-338]. But in meiosis they must also be allowed to duplicate during the abnormal interphase between meiosis I and II, which could be achieved by preventing in anaphase I the normal anaphase proteolytic digestion of the protein SAS-6, whose amount controls centriole duplication [71], the third key step in the origin of meiosis.

Conversely to meiosis, the origin of syngamy requires plasma membrane fusion and also the merger of two parental centrosomes into one or the destruction/loss of one parental centrosome or the destruction of both and the re-emergence of a single one de novo [332]. In principle two centrosomes could merge into one if they lack centrioles as in higher seed plants, but for centrioles that would be mechanistically impossible. In some animals the centriolar reduction associated with syngamy is associated with the loss/destruction of the egg centriole, only that from the sperm being transmitted. In *Chlamydomonas *all four parental centrioles from the isogamous bicentriolar gametes are disassembled in the zygote and new ones are formed in the germinating zygospore shortly before meiotic prophase I [166], as are all their associated microtubular and centrin-containing fibrillar roots. Controlling their proper numbers and assembly in the premeiotic cell would be simplest if the centrioles of one mating type were totally destroyed in the zygote but those of the other remained as two microscopically invisible procentrioles until germination. In the euglenoid *Scytomonas*, whose gametes both have a single cilium and centriole, the zygote is initially biciliate but one cilium is quickly lost and the diploid cell then swims with the other [68]. This suggests that unilateral destruction of one zygote centriole may have been the ancestral condition for eukaryotes.

Meiosis could have started to evolve as soon as cyclin-based eukaryotic cell cycle controls and cohesin evolved. Arguably it did not initially depend on synaptonemal complexes, but these were soon added to increase the mechanical stability of paired chromosomes. As synaptonemal complexes have been casually observed ultrastructurally in one bodonid euglenozoan, this stage must have been reached in the eukaryotic cenancestor. However there are no studies of meiosis in any of the three most deeply branching eukaryotic phyla (Euglenozoa, Percolozoa, Loukozoa), but recombination and Mendelian processes is established for several trypanosomatids. Therefore we do not know if any of these early branching eukaryotes have a bouquet stage of meiosis as in neozoa or whether Sun-KASH nuclear envelope protein roles in meiotic prophase evolved in the first eukaryote or only later, e.g. in the immediate ancestor of neozoa which all have fundamentally similar meiosis.

As mentioned above, meiosis is likely to have arisen prior to the origin of the nuclear envelope, probably as soon as the earliest form of mitosis evolved with primitive centrosomes and centromeres, as the selective advantage of correcting ploidy errors would have then been at its peak and cohesins, the essential molecular precursors of the meiotic machinery, would already have evolved. Explaining both its mechanistic origin and how it was selected are both much easier than if meiosis originated after the nuclear envelope and had to be preceded by the evolution of nuclear fusion, which in the absence of meiosis has no rationale or advantage. Ploidy reduction is a real, powerful, and experimentally demonstrable evolutionary force [339]. Ploidy reduction requires only chromosome pairing and meiotic cohesins and arguably evolved even before the nuclear envelope. The cytoplasmic motor driven congress of telomeres to form the bouquet stage must have followed the origin of the nuclear envelope and Sun-KASH domain interactions across it. Nuclear fusion, an essential part of sexual cycles must also have followed the evolution of the envelope. Thus the evolution of meiosis probably straddled nucleogenesis and was an integral part of eukaryogenesis, not a late evolutionary afterthought. What about syngamy?

I have argued that syngamy evolved marginally after meiosis and its primary functional significance was related to the fact that even the first eukaryotes would have had dormant resting cysts, which I argued evolved from posibacterial exospores [3,63,332,333]. Its prime role was two-fold: to make zygotes larger and increase their survival rate by being able to store more solid food reserves; to provide genetic redundancy in the dormant cysts so that accidental damage, notably double-strand breaks by radiation or other environmental insults or by physiological errors could be repaired by homologous recombination among genomes. Maximising the number of offspring when conditions improved and the cyst could germinate explains why meiosis probably ancestrally occurred then and vegetetative cells were haploid [333]. The feast and famine existence of the first predators on earth thus probably played a key role in the origin of sex, and life cycle ploidy control, its prime raison d'être. Predatory feeding came before sex. I refer readers to other discussions of the likely prime importance of circumventing deleterious mutations in the origin of sex [340,341]. It is now widely considered that removing harmful mutations may be a more important factor in the maintenance of sex than making favourable allele combinations, which sex can equally undo [342-344] and which can also be made by mitotic recombination [345]. The origin of sex cannot be considered apart from the origin of the new eukaryotic cell cycle controls, mitosis, and a wall-free cell surface with internal actin gel cytoskeleton that mechanically allow gametes the ability to fuse and the stability to survive without walls prior to fusion. The origin of syngamy did not just involve the control of plasma membrane and nuclear envelope fusion but also the merger of the cytoskeletons of the gametes. The only Eozoa where meiosis has been widely studied are parasitic Metamonada, where its hormonal synchronization with insect host moulting physiology made it technically easier. In oxymonads, unlike morphologically more conservative metamonads like the free-living *Trimastix *and parasitic retortamonads, the microtubular skeleton is no longer mainly pellicular - instead most microtubules form a giant contractile axostyle organelle deep in the cytoplasm. Remarkably, when oxymonad gametes fuse, their two axostyles also fuse into one [346], illustrating the need in all eozoan sex to halve the number of cytoplasmic organelles as well as fuse nuclei during syngamy.

Even though sex can undo favourable gene combinations as well as make them and always has some cost, the likelihood that syngamy was already evolving in the vegetatively wall-free prekaryote that periodically made dormant walled cysts at times of starvation, would have allowed different lineages of transitional forms to recombine their eukaryotic innovations and could have speeded up the transition. This is because eukaryogenesis differs radically from discussions of the maintenance of sex, which deal with a quasi-equilibrium situation where repeated (and often reversible) mutations of the same general sort may be the main factor and radical innovation is rare [342-345]. By contrast, during eukaryogenesis mutations that had never occurred before in the history of life, rather than recurrent everyday allelic mutations, were of key importance at numerous times in the process. I have given many instances where the origin of new protein paralogues by unique gene duplication and divergence, or radically new protein domains never before found in bacteria, and of totally new domain combinations to make novel multidomain proteins, were decisively important for the origin of the majority of novel eukaryotic proteins, structures and processes. Thus, once basic meiosis had evolved for ploidy reduction, accidental cell fusions between related lineages with different novel useful genes would not be so harmful; by combining independently evolved innovations it could indeed have speeded up the evolutionary transition from bacteria to eukaryotes. I do not think it reasonable to regard this as the driving force for the origin of meiosis, but it could have been a third important reason for improving the cell fusion machinery and restricting its expression to times of dormancy onset, where life was threatened already by famine and the cell had less to lose and more to gain by fusing with others. Fusion during normal growth with abundant prey when cell fusion would have been disadvantageous for a zooflagellate and therefore increasingly stringently repressed. Such condition-dependent sex is much easier to evolve than if every reproductive act is sexual, as the costs are more easily outweighed by the benefits [347]; this obviously applies to protozoa where most reproduction is clonal with cell fusion being rare at best and to bacteria where cell fusion probably almost never occurs in nature - controlled cell fusion began with the first eukaryotes.

Rerooting the eukaryote tree between Euglenozoa and Percolozoa [9] makes it much clearer than ever before that the forms of vegetative cell fusion that make multinucleate plasmodia, exhibited for example by myxogastrid slime moulds (e.g. *Physarum*) in the Amoebozoa (unikonts) and by the naked cercomonad zooflagellates in the Cercozoa (Rhizaria in the corticates) are evolutionarily advanced not primitive characters. Such plasmodial stages in the life history are entirely unknown in Eozoa, and there is now little doubt that the last common ancestor of all eukaryotes had a semi-rigid micro-tubule supported pellicle that would preclude plasmodia formation. Though amoebae did evolve secondarily within Percolozoa to form Heterolobosea [348] (and even in one instance, *Dientamoeba*, within parabasalid metamonads), the main thrusts of eukaryotic soft surfaced evolution were in advanced groups of unikonts, which probably ancestrally evolved myosin II-based pseudopodia [9] (notably in Amoebozoa), and the corticate kingdom Chromista (not only in the ancestrally amoeboflagellate Rhizaria, but also in several independently amoeboid subgroups), which by contrast were probably ancestrally strongly pelliculate zooflagellates without amoeboid stages prior to the internal enslavement of the red alga. But for understanding early sex it is the discicristate zooflagellates with strongly developed pellicles that need to be considered. Sadly, sex is unstudied in any of their free-living representatives; for almost none of them (exceptions one bodonid and the euglenoid *Scytomonas*) do we even know whether they have sex at all or whether it has been lost long ago by many or even most of them, as is perfectly possible. However, if the present rooting of the eukaryotic tree is correct, we can be sure that the cenancestral eukaryote was a pelliculate zooflagellate with dormant cysts, ciliated gametes, meiosis, and a synaptonemal complex. If, as I have argued, sex and meiosis began in a prekaryote with a single cell-surface-associated centrosome containing centrin and γ-tubulin and attached on the one hand to the chromosome replicon origin and on the other to stable microtubules that were permanently present through the cell cycle, then it is relatively easy to see how sexual life cycles could have originated despite their apparent complexity.

If the chromosome replication origin at that stage was also permanently attached to the cell surface, the lateral fusion of two such cells would have generated a zygote with two side by side chromosomes in the same compartment that could form hybrid DNA segments with each other quite easily when single stranded DNA was transiently produced, e.g. during replication or repair. By generating unequal crossover and chromosome breakage they would have been deleterious. Though this would have thereby selected against such fusions, the cell phenotype produced by syngamy, with two side-by-side chromosomes able to recombine, is essentially the same as that produced every cell cycle by replication prior to division. Thus the suppression, control or repair of such adjacent chromosomes exchanges would be important even for vegetative growth. In bacteria such dimers can be resolved by the Xer decatenation machinery at the cell surface at the presumptive division plane [235,349]; similar processes must have been in process during eukaryogenesis; given the magnitude of the changes associated with chromosome internalization on endomembrane vesicles it seems almost inevitable that at some stage chromosomes would be broken, even in vegetative cells merely undergoing binary fission and not engaging in syngamy. Odd numbers of sister chromatid exchanges for circular chromosomes have the same dimerization and consequential breakage potential as meiotic cross-overs, so the linearization of eukaryotic chromosomes and addition of telomeres need not have awaited the evolution of a primitive meiosis and sexual cycle as earlier suggested [11] but was likely as soon as the ordered segregation along the bacterial cell surface broke down during the replacement of the bacterial cell wall by a cell surface coat, endoskeleton and protozoan pellicle. Thus telomeres probably evolved soon after the origin of the first centrosomes. It has been proposed that centromeres evolved from telomeres [350], but the arguments are unconvincing. It was claimed that the fact that only linear chromosomes have evolved proper centromeres means that telomeres must have evolved first [350]. But the argument can easily be reversed; it is just as (il)logical to argue that as proper telomeres only evolved in chromosomes with proper centromeres, centromeres must have come first. As eukaryogenesis was unique, one cannot reasonably use its uniqueness per se, as did these authors, to argue the polarity of any of its component unique events. Instead one must use a logical reconstruction of the likely nature of transitional intermediates, the nature of their probable precursors, the phylogenetic context, and the selective advantage of each postulated stage, as attempted here and in previous discussions [4,70]. Those authors were unaware of those arguments that centromeres arose before telomeres [4,70], wrongly stating that the only past discussion of the origin of centromeres was [351]. The arguments in this paragraph suggest that telomeres probably slightly post-dated proto-centrosomes but could have preceded eukaryotic centromeres and kinetochores. The heterochromatic properties of centromeres and telomeres could have evolved simultaneously, but I still think it likely that centromeres came first as there would probably have been a stronger selective advantage for chromosomes to associate with microtubules than for telomeric functions. As soon as chromosome origins lost their association with centrosomes there would have been selection for secondary association with microtubules via proto-kinetochores. It seems simplest to suppose that these evolved from the bacterial origin region as both are involved in bacterial chromosome movement and that this happened soon after the origin of the nuclear envelope when the ancestral centrosome duplicated to make a separate intranuclear MNC. Telomeres might have evolved either from the chromosome terminus or random breaks elsewhere repaired by adding telomeric repeats; derivation from the replicon origin [4] now seems less likely.

### Nuclear fusion

Nuclear fusion involves both membrane fusion and the integration of the nuclear skeleton into one. If, as I have argued, the first eukaryotes had no proteinaceous nuclear lamina and the inner membrane was attached directly to chromatin by Sun-domain proteins among others, this simplifies the origin of nuclear fusion. One only has to make each membrane fuse in turn, as in *Chlamydomonas *[352] and yeast [353]; for the fusion of the outer membrane proteins involved in ER membrane fusions were probably recruited. Fusion of mitochondria from both gametes also commonly occurs in protists as diverse as yeasts, slime moulds and *Chlamydomonas*, and must also involve fusion of two membranes. I am unaware of any examples in Eozoa, so we do not know whether it evolved early in eukaryote and mitochondrial evolution. It might have occurred relatively late, for example after the ancestral bacterial FtsZ division mechanism was replaced by host dynamin division machinery [354], which could also have helped fusion. Given the multifarious roles of dynamin in membrane division (which also involves membrane fusion) [355] and that it is located on the nuclear envelope in ciliates [356] it would not be surprising if it were also recruited to help with nuclear fusion. Chloroplast fusion also occurs during syngamy in many green algae [352] and clearly evolved much later than nuclear or mitochondrial fusion, but is an important part of some sexual cycles.

### Ploidy cycle evolution

Attentive pedants will have noticed that my saying that meiosis evolved for 'ploidy reduction' prior to the origin of the nuclear envelope is terminologically questionable. This is because the distinction between polyploidy and multinuclearity strictly only applies to eukaryotes with a NE. In bacteria with single chromosomes and lacking plasmids there is no distinction between genomes and chromosomes and the concept of ploidy does not strictly apply. In bacteria filamentous cells containing several nucleoids within one cell (quite common) are more analogous to eukaryotic multinucleate plasmodia produced by delaying cytokinesis compared with mitosis, and are not really polyploids and no special reduction division is needed to reduce their genome copy number.

The neomuran precursor of eukaryotes would conceptually have stopped being a bacterium as soon as centrosomes, microtubules, cyclin-based eukaryotic cell cycle controls, and cohesin evolved, though would not have been a protoeukaryote until the nuclear envelope evolved. But as soon as cell division of this prekaryote became obligately linked to centrosome duplication and anaphase proteolysis of cohesins, it becomes reasonable to regard cells in which this linkage has failed and which contained multiple chromosomes but only one centrosome as analogous to plant autopolyploids. With only one centrosome they would have no way of knowing that they had too many chromosomes or halving their numbers without somehow blocking DNA replication while allowing an extra centrosomal duplication, one of the hallmarks of meiosis. Thus as soon as special meiotic cohesins and partial protection of their anaphase I digestion (on chromosome arms) had evolved one can reasonably speak of prekaryotic meiosis and of ploidy reduction.

It is unclear what was the ploidy of the first eukaryotes as ploidy levels are unknown for free-living discicristates (the fact that some trypanosomatids are diploids does not allow us to infer the condition even for their closest relatives the bodonids, still less for ancestral Euglenozoa or Percolozoa), though it should be possible to deduce whether *Naegleria *is haploid or diploid when its genome sequence is properly studied. I have long supposed that the ancestor would have been haploid and only the zygote diploid, as in *Dictyostelium*, *Crypthecodinium *and *Chlamydomonas*, as that is probably the most widespread condition in free-living protozoa, except for those with very large cells like the somatically polyploid ciliates, the plasmodial myxogastrids or reticulose Foraminifera [3,63,306]. I have argued that selection for large or small cell size played a major role in evolving the diversity of protist life cycles, and that selection for small trophic cells and larger resting cells may have been of key importance in the evolution of haplo-diploid life cycles, including cell cycle variants such as multiple fission (always derived) rather than binary fission (clearly the ancestral state for eukaryotes) [306]. Experimental studies on yeasts are clarifying the selective forces acting on ploidy levels of this normally diploid saprotrophic fungus [339,357-359], but caution is needed in applying these to predatory protists with their very different ecology. Direct studies of discicristate ploidy and life cycles are needed to clarify early eukaryote ploidy evolution.

## Discussion

According to the present coevolutionary theory, the origin of the nucleus depended on the prior evolution of a primitive endomembrane system and a primitive mitosis, both brought about by and associated with the origin of phagocytosis. The revised multistage theory for the simultaneous origin of mitosis and the pellicular microtubule array of the first discicristate eukaryotes offered here (Fig. 3) explains the transition from posibacterial to eukaryotic skeletal, segregational, and cell division structures distinctly more smoothly and in more detail than previous ideas. The new interpretations of the origins of heterochromatin and its positional control by small RNA and the new theory for the origin of the nuclear envelope and nuclear pore complexes by coated vesicle fusion (Fig. 5) integrate a vast body of molecular and cell biological data into a coherent picture of how nucleocytoplasmic compartmentation originated, with plausible mechanisms and selective advantages for each stage. The discussion of the origin of meiosis and syngamy explains the origin of sex and of eukaryotic life cycles with alternating ploidy levels as the almost inevitable outcome of those new mechanisms of mitosis, cell cycle control, and cell compartmentation, given the need to reduce cell cycle errors in ploidy, maximize survival in dormant states induced by famine or environmental adversity that precludes growth, and the contrasting requirements for maximizing reproductive rates when food is plentiful. This is all done within a critically interpreted phylogenetic framework for both the ancestral bacterial and the derived eukaryotic parts of the tree of life, which is consistent with all major lines of evidence, molecular, cellular, and palaeontological.

In comparison with the above proposals, previous discussions about the origin of the nucleus are either unnecessarily complicated or fail to explain the most essential things. This is particularly the case with theories that invoke symbiogenesis or prokaryotic cell fusions as 'explanations' of the basic features of eukaryotic cells. To illustrate such unnecessary complexity and explanatory failure, consider the serial symbiogenetic/autogenous theory of my good friends López-García and Moreira [122]. I single this out for detailed criticism not because it is worse than other cell fusion/symbiotic theories but because it attempts to be more detailed, which is praiseworthy; most are so empty scientifically that a critic can gain no useful purchase.

### Small GTPase origins: vertical inheritance, lateral transfer or extra symbioses/cell fusions?

The ancestral eukaryotic small GTPase gene gave rise to about 10 functionally distinct paralogues [124] during the later phases of eukaryogenesis, all important for controlling the cytoskeleton, secretion, phagocytosis and nucleocytoplasmic exchange (e.g. the Ran GTPase). For some years Moreira and López-García have argued that the closest relatives of these small GTPases, are absent not only from archaebacteria and posibacteria but also from α-proteobacteria, but are present in myxobacteria (δ-proteobacteria) [360,361]. They thought that eukaryotic small GTPases came from neither the host nor the premitochondrial symbiont. If such GTPases were truly absent from both host and mitochondrial lineages, the simplest interpretation would be that the prekaryote instead acquired a small GTPase gene by lateral gene transfer (LGT) from a negibacterium; presumably one that it ate in the earliest days of phagotrophy after actin and endomembrane vesicle traffic started but before control by small GTPases evolved [3]. However small GTPases of the BglA family are widely present in negibacteria, not just myxobacteria [3]. They have also now even been found in α-proteobacteria and several other paralogue families have been discovered in bacteria [128]. Most important is the RarD family restricted to actinobacteria and archaebacteria. This is the prime candidate for a direct vertical ancestry for all eukaryotic small GTPases making LGTs or more complex scenarios pointless.

Yet Moreira and López-García [360,361] imagined that the nucleus evolved as part of a three way symbiosis in which a myxobacterium was the host for the engulfment and enslavement of an archaebacterium and an α-proteobacterium. Supposedly, the initial stimulus for this was metabolic syntrophy between the different bacteria, followed by serial endosymbiosis, also metabolically driven. In the latest version of this syntrophy theory [122] the archaebacterium became the nucleus, but its membrane was lost, as were the outer membrane and genome of the myxobacterium! Not only is this unnecessarily and excessively complex, but its logic is fundamentally flawed, and it explains nothing important about eukaryote origins. None of the 27 novelties of Appendix 1 is explained, most not even being mentioned. The theory states that a myxobacterium phagocytosed first a methanogenic archaebacterium, to which it supplied CO_2 _and hydrogen for making methane, and then a methane-oxidising α-proteobacterium. Then the myxobacterium grew surface membrane invaginations to surround the archaebacterium, supposedly to help the archaebacterium secrete proteins into the environment across the three membranes and two cell walls that then surrounded it (its own cytoplasmic membrane and cell wall and the cytoplasmic membrane, peptidoglycan wall and the outer membrane of its host myxobacterium). How this could possibly help secretion across these five barriers was not explained, nor was it stated what specific proteins were secreted or any selective advantage of that secretion mentioned. Despite these omissions that render it explanatorily empty, that membrane invagination was put forward as the primary selective 'explanation' of the origin of the nuclear envelope.

In the next hypothetical step the archaebacterial membrane was lost but its genome took over as the main genome of the cell, with the loss of the myxobacterial genome after transferring most metabolic genes into the former archaebacterial genome [122]. The physical mechanism of loss of the methanogen's membrane was entirely unspecified, presumably because no plausible mechanism exists. I cannot imagine any DNA mutation that could achieve that or any selective advantage for it. Indeed there is no known case in the history of life when one cell living inside another lost its bounding membrane and survived. If a bacterium thus lost its cytoplasmic membrane, it would be disastrous for DNA segregation that depends on membrane proteins. This theory says nothing whatever about the transition from bacterial DNA segregation to mitosis, and is physically and cell biologically absurd. The same is true of all the numerous 'theories' that invoke a chimaeric fusion of two bacterial cells prior to the origin of mitochondria, e.g. [120,362-364]; most of the others do not even attempt to explain the origin of the nuclear envelope or mitosis, so one cannot explicitly criticise their logic, if they have any - which is not evident. The next flaw concerns the claim that the next selective advantage for wrapping the secretory membranes around a former methanogen was that it would prevent the harm done by putting opposing metabolic pathways, e.g. methanogenesis and methane oxidation, in the same compartment. This is illogical, because that harm only comes when you remove the archaebacterial membrane. Since the authors argue that methanogenesis mechanically requires the membrane and was lost with the membrane, there never could have been any selective harm from having methanogenesis and methane oxidation in the same compartment. To suggest that such a selective advantage could favour the growth of the membrane prior to archaebacterial membrane loss is equally unsound; it is impossible mechanistically, as it assumes that natural selection has foresight, essentially a creationist attitude not a biological one. Nor can one specify any metabolic segregation that making the nuclear envelope could alleviate. Nuclear pores are so large that any metabolite could diffuse through them.

The authors then suggest that the next selective advantage for the further evolution of the nuclear envelope was to prevent the hypothetical harm done by the origin of spliceosomal introns if they evolved when transcription and translation were in the same compartment [81]. This is precisely the same as Martin and Koonin's [21] suggestion for the primary selective force for the origin of the nuclear envelope. Both proposals are equally illogical, failing as explanations. First, if as I originally argued [81] this was such a strong selective forces that it prevented the evolution of spliceosomal introns for 3.5 billion years in all bacteria, and for 800 My in all mitochondria and chloroplasts, which all have only rapidly self-splicing introns, there is no reason to postulate that spliceosomal introns evolved before the nuclear envelope - a gratuitous assumption devoid of evidence. Secondly, if they did evolve before the envelope, the damage was already done, and could not be reversed by making an envelope. Thirdly, as explained above, the earliest stages of nuclear envelope evolution would not have excluded ribosomes from the nucleoplasm, and thus selection to separate transcription and translation cannot have been the initiating force.

Another flaw in the myxobacterial theory is that myxobacteria have two bounding membranes, so the outer membrane would have to be lost to make a eukaryote. Losing a negibacterial outer membrane is very difficult; I have argued that it only happened once in the history of life (during the origin of Posibacteria from negibacteria) and that the physical mechanism was by mutation-induced murein hypertrophy making the wall so thick that contacts between cytoplasmic membrane and outer membrane were suddenly physically broken [4,13]. Neither López-García and Moreira [122] nor others have ever suggested another physical mechanism for losing the outer membrane. Yet they assume such loss, citing the loss of the outer membrane by Posibacteria as an historical precedent. But that is irrelevant unless the same mechanism also is assumed, which they do not suggest. In fact, they ignore both the mechanism and the selective advantage of the loss, making the theory scientifically empty and unrealistic. It is however gratifying that they now accept the origin of posibacteria by outer membrane loss as an historical fact, despite López-García as a referee strongly criticising my conclusion of its truth, mislabelling it an assumption [13] rather than a deduction by reasoned argument.

Another defect of their theory is its assumption of two successive phagocytic uptakes of foreign bacteria, coupled with the contradictory assertion that phagocytosis itself evolved after the origin of mitochondria and the nucleus. This is unparsimonious and illogical. No bacteria have phagocytosis. There is not even one known example of free-living eubacteria that take up other cells into their cytoplasm by any mechanism. To postulate that something never known to happen did so twice in one cell prior to the origin of eukaryotes is not the most parsimonious explanation of their origin. It is almost infinitely more likely that the mitochondrial enslavement was after prekaryote cells started to evolve phagocytosis rather than before. Exactly as did Martin [26] for the hydrogen hypothesis, refuted in detail previously [3], López-García and Moreira [122] make a spurious defence of their prokaryotic host theory by citing the discovery of a cellular symbiont with a proteobacterium that is itself a parasite of eukaryote cells, which they claim invalidates my criticisms of prokaryote host theories like theirs. It does not. It is unreasonable to extrapolate to a free-living cell from a parasite, which has a more stable environment for the origin of intimate associations, and in which cell wall peptidoglycan can become thinner or even be lost (mycoplasmas, chlamydias), and therefore weaken one barrier to rare cell uptake. However, the criticism of host prokaryote theories would not be invalidated even by the discovery of several examples of one free-living bacterium engulfing another. It would remain true that almost all free-living protozoa can engulf other cells any day, so this has happened trillions of times in the history of life; moreover there are thousands of examples of symbionts surviving uptake; but at present not one example for free-living bacteria. It is therefore undeniable that uptake of another cell by a protozoan is vastly more likely than by a free-living bacterium. On grounds of parsimony and likelihood, one should not invoke uptake of a bacterium by another prokaryote as the initiating step in eukaryogenesis unless there were compelling evidence that requires us to do so. There is no such evidence.

The belief that many eukaryotic genes exist, whose source cannot be explained as from either our neomuran or α-proteobacterial ancestors [122], is phylogenetically unsubstantiated. Even if it were true for a few - or even many genes - it would not justify the cell biologically impractical and evolutionally unrealistic suggestion of cellular fusion or chimaerism among prokaryotes. Instead, the proven ease with which phagotrophs can acquire genes from their prey [365] would suffice to explain their origin. It is puzzling why Moreira and López-García [360,361] assumed cell fusion, not the much simpler LGT, to explain the origin of a few additional genes (if any clearly required it, which almost none do). Neither they nor any other proponents of prokaryote host theories going back to Sagan [366] have ever validly criticised the phagotrophy first theory [367]. The even more complex symbiotic [122] rather than syntrophic theory rested its assumption that phagocytosis was relatively late in eukaryogenesis on Jékely's fallacious argument that exocytic secretion came first [107], which I refuted above. López-García and Moreira [122] wrongly wrote that Jékely [107] thought that a Sar1-like GTPase was acquired by myxobacteria via LGT from eukaryotes; he did **not**, instead assuming, probably correctly, that such eubacterial GTPases, which his tree also included for other negibacteria, were the outgroup by which he could root his tree! The assumption that obligately anaerobic methanogens (derived euryarchaeotes) provided either the cytoplasm [26] or the nucleus [122] of the eukaryotic cell are both phylogenetically refuted by the evidence that archaebacteria as a whole are sisters of eukaryotes, not ancestral to them [12,24]. They are phylogenetically wrong, cell biologically flawed, and explain nothing important.

Even LGT probably had only a minimal role in eukaryogenesis. One possible example is the six NE-associated proteins with homologues in cyanobacteria but no other eubacteria [14]. It needs to be established whether LGT was from eukaryotes to cyanobacteria or the reverse. If cyanobacteria were donors, it would be intriguing to know if any of these proteins are associated with thylakoids, invoked as precursors of the NE in the original version of the phagotrophy theory [11]. However, at present the dominant genetic aspect of eukaryogenesis is a massive origin of new genes by gene duplication, radically sudden divergence (often beyond recognition by sequence alone) of genes already present in the actinobacterial ancestor of neomura, and gene chimaerization to yield novel domain combinations, all in response to the novel cellular membrane topology and endoskeleton produced by the unique evolution of phagotrophy.

### Inadequacy of other theories

Sometimes the internal membranes of Planctomycetes [368] are invoked as possible precursors of the nuclear envelope. However, this is a complete red herring as they are totally irrelevant - crude analogies at best. There has been no serial sectioning analysis to show that they actually define a distinct cellular compartment separating nucleoid and cytoplasm. The simplest interpretation of the published pictures and others I saw when visiting Fuerst is that these membranes are unusually extensive invaginations of the cytoplasmic membrane, topologically equivalent to proteobacterial chromatophores, albeit much more extensive. Possibly their stronger development than in most negibacteria has something to do with the fact that the ancestors of Planctomycetes lost the murein wall and evolved a proteinaceous layer between the cytoplasmic and outer membrane; conceivably this layer can extend inwards to help support more extensive membrane that can murein. To call the openings sometimes seen in these membranes 'NPC-like' [122] is sloppy. There is no evidence whatever that they are structurally related. Nor is there any evidence from planctobacterial genomes for a specific relationship with eukaryotes; they are far away from both eukaryotes and archaebacteria on all multigene trees. The idea that a negibacterium with two bounding membranes, as in Planctobacteria, could be ancestral to phagotrophic eukaryotes is cell biological nonsense, especially for Planctomycetes as there is no known mechanism for losing the outer membrane in peptidoglycan-free negibacteria. Even their *Chlamydia *relatives, among the most reduced intracellular parasites, never lost the outer membrane despite losing murein and ATP biosynthesis; nor did mitosomes, the most phylogenetically reduced negibacterial descendants of all, which additionally lost their entire genome [369].

The discovery of α- and β-tubulin genes in some Planctomycetes is also irrelevant to eukaryote origins, as they are evidently lateral gene transfers from eukaryotes [370-372], evolving so much faster than any others that they cannot possibly be ancestral to eukaryotic genes and probably lost microtubule functions and evolved others instead with weaker stabilising selection. Just as one swallow does not make a summer, two tubulins do not make a eukaryote. Several others are needed, as are hundreds of other cytoskeletal proteins.

The latest version of the spirochaete theory of the origins of cilia, mitosis and the nucleus [120] is as devoid of phylogenetic support, explicit transformational reasoning, and cell biological plausibility as the earlier versions dating from Sagan [366], so past criticisms [366] still apply. It is imagined that a sulphur-reducing archaebacterium, like *Thermoplasma*, the smallest archaebacterium, successfully engulfed a much larger spirochaete (with two bounding membranes) and converted it into a mitotic spindle and cilium and the genomes of the two bacteria merged to become the nucleus. How any of this could have happened is totally unexplained. Early predictions of the theory that spirochaetes would have tubulins and other precursors of spindles and cilia have been firmly refuted, but instead of being properly rejected the 'theory' is now emptied of all content that might allow refutation, making it pure science fiction. The complexity of cilia with about 1000 proteins, most novel to eukaryotes and many related to non-ciliary eukaryotic proteins, always made it naïve to think of their origin primarily in terms of where tubulin came from.

By comparison, the autogenous theory [4,238-240] according to which cilia are very complex modifications of many disparate elements of a simpler cytoskeleton that evolved in the earliest eukaryotes remains valid and unrefuted, and goes from strength to strength. Though I do not agree with all details in the latest formulations [122,373], which can be further modified and improved, they are firmly founded on real cell biology and careful reasoning. An important point is that the ciliary transport particle, essential for ciliary biogenesis, contains α-solenoid and/or β-propeller domains, and so like the NPC may also have evolved from transport vesicle coats [374]. This makes it plausible that the origin of coated vesicles was not only the cause of making endomembranes permanent but also an essential prerequisite for the origin of both nucleus and cilia, whose origin is therefore subsequent to that of endomembranes. The recent rooting of the eukaryotic tree indicates that the cenancestral eukaryote had both a nucleus and an attached centriole and cilium: what classical protozoologists called a karyomastigont. The theory of the simultaneous origin of cell nuclei and centrioles and cilia is therefore correct [4]; it is accepted even by Margulis [120], who unfortunately also retains its associated postulate that some extant protozoa are primitively without mitochondria, now firmly disproved [106].

We now see that the origins of mitochondria, nuclei, and cilia were virtually simultaneous. As each was a complex series of processes it is most likely that they overlapped in time, so disputing which was first may be meaningless. One clue to relative timing is that ubiquitin is essential for spliceosomal assembly [375]; this suggests that ubiquitin was already present before the mitochondrion donated group II introns to the nucleus, allowing the origin of spliceosomes. As the role of ubiquitin in proteasomal digestion of faulty ER proteins and cell cycle proteins is likely more fundamental, these mechanisms probably evolved prior to the origin of mitochondria; thus the host was probably already eukaryotic in most respects prior to its enslavement and the origin of spliceosomes. Ubiquitination probably evolved very early in eukaryogenesis as soon as the endomembranes became stably separate from the cell surface [27]. That paper proposed that proteasomes played a key role in the origin of eukaryotes immediately before the origin of phagocytosis, by digesting prey proteins translocated directly across the ancestral plasma membrane, and that ubiquitination evolved after this hypothetical intermediate evolved phagocytosis and lysosomes, making such digestion of prey proteins redundant.

### Was ribosomal chimaerism the major selective factor in pore-complex origin?

Since this paper was written a new suggestion about the selective force initiating nuclear evolution appeared [376]. This builds on my earlier idea that the symbiotic origin of mitochondria and transfer of mitochondrial ribosomal protein genes to the nucleus would have influenced cytoplasmic ribosome evolution, because of selection to avoid chimaeric cytoplasmic ribosomes with some incorporated mitochondrial ribosomal proteins [3,377]. I argued that cytoplasmic and mitochondrial rRNAs and proteins diverged to reduce the extent of heterologous misassembly. Jékely [376] proposes that this selective force would have been strong enough to favour also the origin of the nuclear envelope and pore complex. He notes that 24 mitochondrial proteins were transferred to host chromosomes before the eukaryote cenancestor, assumes that this happened before the origin of the nuclear envelope and caused enough harm to stimulate the origin of the envelope. I am unconvinced, for several reasons, the first two already expressed by Forterre, who refereed his paper [376]. First, there is no evidence that the mitochondrion evolved before the nuclear envelope; Jékely himself earlier noted, as I had previously, that the recent conclusion that the ancestral eukaryote already had mitochondria has been misinterpreted as evidence for mitochondria being enslaved by a eukaryotic host before the origin of phagotrophy, when it is nothing of the kind. This is not a strong objection because I suspect that they evolved roughly simultaneously. Secondly, supposing that the transfer of genes could be tolerated with impunity for a period and then became a compelling force for evolving the envelope is problematic. This explanation is like the spliceosomal intron one in putting the damage first with a rescue later: the cart before the horse. If transfer were so strongly disadvantageous, it would be more likely either that cells with transferred genes would lose out in competition or that host genes and transferred genes were simply modified to reduce the problem, as I proposed earlier (we know that substantial modification took place, though whether the reason I gave is the best explanation is hard to test). However, there is little doubt that sometimes cells do suffer non-lethal harmful effects that are later phenotypically corrected in roundabout ways, so this style of reasoning is not intrinsically unsound; but its plausibility hinges on difficult judgements of selective disadvantages and advantages, and the likelihood of different modes of correction and weighing all these against other likely factors that are hard to evaluate. Thirdly, the benefits of differential concentration and nucleocytoplasmic distribution would have been the net benefit of excluding hundreds of different proteins from the nucleus and the positive import of hundreds of others. If avoiding ribosomal chimaerism were one of them, it was probably of such minor importance that the nucleus would have evolved as it did without it. Singling out exclusion of one class of proteins as markedly more critical than others is very arbitrary. In any case, my earlier suggestion of a modification of host ribosomal proteins and RNA (helping to causing their long-known much greater divergence from prokaryotic ancestors than for mitochondrial ribosomes) [3,377] would be a much simpler and more direct way of reducing the problems of ribosomal chimaeras.

Nonetheless, Jékely's [376] simulation studies of concentration gradients between localised chromatin and cytoplasm in a cell without full nucleocytoplasmic compartmentation and transport are extremely important as showing how in principle proteins that can bind to chromatin can be localised and influence the local concentration of interactors/product in ways beneficial to the cell. Thus even though I am sceptical of a dominant role being assigned to the avoidance of ribosomal chimaerism, the principles he demonstrates can be applied to all kinds of molecules whose concentration collectively could have provided a strong selective force at all stages of nuclear envelope evolution. It is highly likely that many molecules were initially concentrated in chromatin regions by binding directly or indirectly to chromatin or by being generated by enzymes that were thus bound. The origin of the pore complex and nucleocytoplasmic exchange was so complex that many components must have evolved initially independently and been combined in successive selectively advantageous stages. As a general principle, compartmentation can explain the origin of the nucleus irrespective of whether it slightly preceded, slightly followed, or was contemporaneous with mitochondrial enslavement. That makes it a more general explanation that unlike Jékely's is insensitive to the precise order of events; his interpretation is a special case of the general principle.

### The primacy of the precursor and mutation; selection is a secondary metaphor

Many theories of eukaryote origins and other megaevolutionary events make the mistake of assuming that selection is the primary force in evolution. It is not. Selection is simply a consequence of a mutation and the environment in which it is found; it is not a third force, or even a force at all. In a stable environment, a novel mutation increases reproductive success, decreases it or is neutral. The relative increase over generations of individuals bearing a novel beneficial mutation is a direct consequence of the phenotype of that mutation; its most important phenotypic property is that it increases reproductive success. Selection is just a metaphorical name given to the mathematical fact that genotypes that inherently increase that success necessarily spread at the expense of those reduce it. Thus major innovation comes only from within the organism by mutation and is not imposed from outside by the environment or a designer or even 'natural selection'. The environment was irrelevant except in a very general permissive way for the origin of eukaryotes. The possibility of an organism getting its food by eating another existed even before the origin of life. There is no reason to think that the environment prevented phagotrophy and the origin of the nucleus for the roughly 2.8 billion years that elapsed between the origin of the first bacterial cell and the origin of the nucleus. The possibility of syntrophy existed for billions of years without making eukaryotes, and the possibility of being an aerobic eukaryote heterotroph existed for about 1.6 Gy before the first one evolved. Eukaryogenesis was so long delayed because of the exceeding difficulty of evolving phagocytosis in a eubacterial cell enclosed by a rigid corset of murein peptidoglycan, and because replacement of murein by a more flexible glycoprotein coat that allowed the evolution of phagocytosis was itself so difficult and unlikely that it did not happen till 2.8 billion years after life began [6]. Thus the nature of existing precursor organisms limits evolution. Most changes are harmful and eliminated by selection. Selection for efficient function in bacteria prevented the origin of the nucleus for nearly three billion years. No selective force could make a normal eubacterium or even a normal archaebacterium evolve a nucleus. There would be no advantage in giving up its traditional attachment of DNA to the cytoplasmic membrane and DNA segregation by membrane motors.

Only the forcible disruption of prokaryotic cell organization by novel forces - actomyosin, mediating phagocytosis - could have triggered such a radical change. No DNA mutation ever directly made endomembranes. Actomyosin provides the real physical force that powers our muscles, enables speech, writing, eukaryotic cell division, and phagocytosis, thereby making endomembranes. The first proto-endomembranes are easily understood as incidental physical consequences of phagocytosis [3,4,11]. Membrane heredity was as important as DNA heredity in stabilising them as distinct genetic membranes [85]. The origin of eukaryotes involved no metabolic innovations, unlike much bacterial evolution. At its core were structural innovations in the cell skeleton and its association with membranes, catalysts and genes. Only phagotrophy first and intracellular coevolution theories provide a clear logical explanation of the origin of the nucleus and of the way in which endomembranes, cytoskeleton, and eukaryotic chromosomes are so mutually interdependent in their functions. They must have coevolved. Enslavement of a proteobacterium to make a mitochondrion provided no novel metabolism. It merely made phagotrophy more efficient in an already aerobic host by improved division of labour by compartmentalisation [3]. Metabolic/bioenergetic theories of eukaryogenesis fail to explain why or how eukaryotes evolved. Prokaryotic host or prokaryotic cell fusion theories are incompatible with cell biology and have explained nothing of significance, yet may remain popular among those who avoid the fundamental issue of radical cellular transformation and how to explain it plausibly without assumptions that would kill the cell.

### Novel adaptive zones, new body plans, and megaevolution

Eukaryote origins has all the hallmarks of what Simpson [10,113] called megaevolution; the origin of major new body plans that distinguish higher taxa like phyla, classes and orders. He argued that such evolution occurred by the normal processes of mutation and selection but was exceptionally fast and radical in its consequences. He showed that the fossil record indicated that body plan innovation invariably occupied a very short time compared with the subsequent history of an innovatory new body plan. Almost invariably the most important adaptive radiations of a new major type also occur relatively soon after its origin with subsequent megaevolutionary stasis. New inventions, like arthropod cuticle and jointed limbs, land plant cuticle and vascular systems, the vertebrate jaw or tetrapod limbs, create what Simpson called a new adaptive zone (in plainer language, a novel way of life). Within it an immense variety of organisms able to fill related but distinct niches can relatively easily evolve by making minor variants of their shared body plan. New body plans always develop by gradual piecemeal modifications of pre-existing ones, but gradual does not mean slow! Typically they do so very suddenly, because transitions from one major adaptive zone to another are difficult because of the sharply conflicting selection pressures that apply to them. Unless the right organism is present in the right place at the right time and experiences a suitable succession of lucky chances, it will not happen - especially if the properties selectively advantageous in the new zone are disadvantageous in the old one.

Megaevolution, quantum evolution, and mosaic evolution are distinct but complementary concepts. Quantum evolution can occur independently of megaevolution. It is not necessarily associated with a novel body plan or higher taxon - it could just affect one molecule that suddenly found a novel function, e.g. following a gene duplication. It refers only to the rate being abnormally high - way beyond the normal range. Mosaic evolution simply refers to a basic truth about organisms: they are not homogeneous, so different parts can evolve in different ways at different times and vastly different rates. All three are just descriptive terms; they do not invoke special mechanisms beyond mutation and selection. This does not lessen their key importance for accurately portraying the pattern of evolution and countering the mistaken view that it is uniform in rate and mode throughout history. We need to understand not only the basic mechanisms of mutation, selection, and symbiogenesis, but also how historical and phylogenetic preconditions, environmental changes, and chance at many levels have combined with these to shape the tree of life into a unique unrepeatable and extremely non-uniform historical record. In other words understanding evolution demands not only mechanistic analysis but also critical historical synthesis. Haldane, the pioneer of population genetics, was well aware of the necessity of adding a detailed organismal and historical dimension to it to give a realistic picture of the causes and pattern of evolution [378] - much more so than some of his successors who mistakenly suppose that population genetics and evolutionary biology are one and the same. It was Simpson who first really tried to make such a synthesis, but it needs to be updated every generation.

Simpson correctly argued that the most dramatic innovations occur when an adaptive zone never occupied before in the history of life originates, as was true for phagotrophy and eukaryogenesis. This is because the first organism able to exploit an entirely novel lifestyle has no competitors in its new niche, and can multiply and leave diversified descendants even if it is relatively inefficient at exploiting it compared with its descendants that later perfected the new body plan. However, its very inefficiency means that there will be exceptionally strong selection for directional improvement until few further gains can accrue. Fast improvement is therefore inevitable given the initial success of the new body plan, and ensures that intermediates will die out before they can significantly radiate, so are never alive today for study and were so transiently and locally present that the chances of their fossilization are exceptionally low. Once they become so efficient that further improvement can only be minor, stabilising selection inhibiting further radical change once again becomes dominant, until the next megaevolutionary breakthrough. The central logic of megaevolutionary innovation, easily recognised when discussing major shifts in habitat such as from sea to land, also applies to major functional shifts, e.g. from fin to leg, from leg to wing, where stabilising selection normally prevents incipient changes that might normally lead to a really dramatic shift. Anthropomorphically put, generally it is better to do what you already do best than to try something so radically different that the chances of a successful transition to its new requirements are very low, often effectively zero.

Mass extinctions never completely emptied a really major adaptive zone or eliminated whole phyla, so no new phyla ever arose as a result. However, some did totally extinguish a few classes and more orders, providing opportunities for other members of the same or adaptively similar phyla to make mid-level megaevolutionary innovations and fill the vacated adaptive zones. In such cases environmental change indirectly caused the timing of some, relatively minor, megaevolutionary events, such as the replacement of one kind of reef-building coral by another or one molluscan or reptilian group by another. There is however no reason whatever to think that abiotic environmental change stimulated any of the most important innovations in the history of life: the origins of phyla or kingdoms, which arose less than 60 times in 3.5 Gy, were limited by the difficulty of the transition and availability of suitable precursors [6]. All or almost all the 48 extant eukaryote phyla probably arose after the melting of snowball earth ~635 My ago, most within 50 million years of the Cambrian/Precambrian boundary (530 My); only four phyla evolved substantially later, the terrestrial fungi (Ascomycota, Basidiomycota) and land plants (Bryophyta, Tracheophyta), being delayed by over 100 My simply because of the difficulty of the transition to land [6]. These Cambrian explosions of phyla are attributable simply to the previous absence of eukaryotes to serve as ancestors. Thus their late origin was triggered internally and biotically by the origin of a suitable ancestor, not by external abiotic factors. By contrast eight of the 10 bacterial phyla (all negibacteria) had probably arisen by 2.9 Gy ago, with only unibacteria (Posibacteria and Archaebacteria) arising significantly later, archaebacteria being the most recent of all - coterminous with the eukaryotes, and possibly triggering the Neoproterozoic snowball earth [6]. Most bacterial phyla could have arisen within a few million years of the origin of oxygenic photosynthesis in the only really major explosive adaptive radiation prior to the Cambrian explosion. Thus on the grand sweep of earth history there were only two major megaevolutionary radiations in the history of life: that of eubacteria consequent on the origin of the bacterial cell; and that of eukaryote phyla stemming directly from the origin of the eukaryote cell [6]. Over long time scales and across major transitions the assumption of uniform rates of change for functionally significant structures or sequences is always wrong, sometimes grossly so, notwithstanding its approximate truth for functionally less significant sequences in some lineages for comparatively brief periods.

### Conclusion: phagotrophy, the novel adaptive zone that made the eukaryotic cell

Now that we are reasonably certain that the ancestral eukaryote was a phagotrophic protozoan [9], not a non-phagotrophic photosynthetic alga or osmotrophic fungus, as on some past now firmly rejected theories, it is beyond serious question that eukaryogenesis involved the origin of phagotrophy [91]. De Duve [131], who discovered lysosomes and peroxisomes, and Stanier [2], apparently independently, first emphasised the key role of phagotrophy in explaining the origins of the endomembrane system. Stanier [2] proposed that phagocytosis was also the stimulus for the evolution of larger cells, microtubules and cilia. Then my coevolutionary theory [11] argued that compartmentation by endomembranes and the cytoskeleton must have coevolved and together fundamentally changed the chromosomes and gene expression machinery, because DNA segregation, transcription and ribosome biogenesis are tied up with cell structure and compartmentation. It proposed that loss of the bacterial peptidoglycan wall and subsequent origin of actomyosin were the primary stimuli for the origin of phagocytosis and the nucleus [11]. Thus the genetic peculiarities of eukaryotes were indirectly also consequences of the origin of phagotrophy in a wall-free prokaryote [4,5,11,163,379,380]. No compelling evidence or arguments have yet been found against this thesis, yet the idea that the novel genetic systems of eukaryotes are a consequence, not a cause, of their feeding behaviour and novel cell structure has not been popular, perhaps because of a widespread misconception that DNA makes cells. It does not; cells make DNA, which is an inert informational repository. Proteins and RNA are the catalysts and effectors; lipids, proteins, and glycans the builders. Trophic behaviour is the architect within the ecological landscape. DNA is not primary, but is important. We must explain its reasonably efficient segregation to daughter cells across the prokaryote/eukaryote divide.

Recently De Duve [367] reappraised the evidence for eukaryotic origins in the light of modern molecular cell biology and now accepts that peroxisomes were early offshoots of the endomembrane system [3,11] and that theories that he and I once espoused of a separate symbiotic origin for them are entirely superfluous. According to the coevolutionary theory of the eukaryote cell, cytoskeleton, endomembranes, peroxisomes, cilia and genetic systems are part of a unified whole, within which food came first and sex was an afterthought [3]. The autogenous origin of all these structures was far more radical than the minor tinkering when the resulting phagotroph converted an engulfed but undigested proteobacterium into a mitochondrion - another afterthought [20]; ultrastructurally and functionally mitochondria are still easily recognisable as slightly modified negibacteria. Intracellular coevolution is strongly exemplified between the endomembrane system and cytoskeleton; and in the way that internalization of membrane-attached DNA revolutionised DNA segregation [70]; in how the origin of centromeres led to other eukaryotic chromosomal properties through mutation pressure [70]; in the way the origin of motor ATPases provided selective forces for nuclear origins; and the origins of coat proteins by duplications both diversified endomembranes into ER, Golgi, endosomes [4] and yielded NPCs and ciliary transport particles [374] necessary for the simultaneous origin of cilia from astral microtubules attached orthogonally to the cell surface by transitional fibres [4].

It is thus hardly surprising that except for mitochondria piecemeal discussions of the origin of only one eukaryotic component largely failed. Symbiogenetic theories, especially, have been a 40-year distraction from the core problems of how a bacterium was transformed into a eukaryote.

## Competing interests

The author declares that he has no competing interests.

## Appendix 1

Key innovations in the origin of the nucleus and eukaryotic cell cycle

1. Internalization of DNA attachment sites as protoNE/rough ER [3,4,11,380]

2. Cell division by actomyosin not FtsZ [3,4,6,11,70,240]

3. Chromatin condensation cycle: histone phosphorylation, methylation, acetylation; heterochromatin [18,170]

4. Mcm replication licensing system controlled by cyclins [244,251]

5. FtsZ triplication to make tubulins; (γ for centrosome) and α and β for microtubules fixing it to cell surface [3,4,6]

6. Kinesin to separate centromeres via antiparallel microtubules [6]

7. Centromeres/kinetochores (CenpA from core histone) for attaching DNA to microtubules [3,4,6] (note that as discussed in the text CenpA might be a property of neokaryotes only, but even Euglenozoa have some kind of kinetochores)

8. Dynein for moving cargo towards the minus end of microtubules and related midasin for ribosome export [6]

9. meiosis and synaptonemal complex [3,63,70,332,333,381]

10. telomerases and telomeres [3-5,70]

11. post-transcriptional gene silencing [177,199,201,203,382]

12. proteinaceous interphase nuclear matrix with bound DNA-topoisomerase II and its ability to reorganize as mitotic chromosome cores [163]

13. nuclear lamina [18,163]

14. nuclear pore complexes (NPCs) [14-17,85]

15. nucleolus and more complex rRNA processing (e.g. 5.8S rRNA) [3]

16. Ran GTP/GDP cycle for directionality of NE export/import [14,17,107]

17. karyopherins [14,17]

18. ribosome subunit export machinery [14,17] (note that most proteins involved have not been detected in Euglenozoa so they might use a simpler system than neokaryotes)

19. mRNA capping and export machinery [14,17,70]

20. polyA transcription termination system [70]

21. 26S proteasomes [27]

22. ubiquitin system [329,331,383,384]

23. sumoylation of nuclear and other proteins [318-323]

24. cell cycle resetting by anaphase proteolysis [6,13,18]

25. nuclear envelope fusion and syngamy [332]

26. spliceosomes and spliceosomal introns [4,70,81,211,385]

27. nonsense-mediated mRNA decay [314]

Innovations are listed in likely order of occurrence, but some (e.g. 3-6, 17-19, 22-23) were likely to have been simultaneous. Some were likely to have been rapid consequences of others (e.g. 9 of 1-7; 16-20 of 13,14; 26 & 27 of 14; 27 of 26). References indicate where their evolution or molecular basis is reviewed in more detail.

### Reviewers' comments

*Anthony Poole*

This is a fascinating and important paper on a complicated topic. Overall, I rate Cavalier-Smith's take on the evolutionary origin of the nucleus as the most thoroughly considered attempt published to date, and there is little doubt in my mind that the main conclusions he makes are better supported than those of competing models. I see no significant problems with the theory as presented, and agree with the central points.

There are two general take-home messages here. The first, eloquently summarised in the final paragraph of the paper, is that symbiogenetic theories have been a '40-year distraction'. Cavalier-Smith's forceful and cogent attack on these views makes for enjoyable reading (though perhaps not if one is on the receiving end), and the arguments here amply demonstrate why the position advanced by Yutin and collegues in their recently published paper (Biology Direct 2009, 4:9) is specious at best. The second general message is that, to have any hope of explaining the origin of the nucleus, it cannot be considered in isolation from other key eukaryotic cellular features (as has all too often been the case).

The only real gripe I have with this paper concerns its proportions. It is like a sandwich made with bread sliced too thickly, and meat sliced too thinly. The Introduction is an interesting though long and, at times, meandering read, and the Discussion is nine tenths diatribe (on why everyone else is utterly wrong on almost everything). Let me make it clear that I don't have any major concerns about the content of these sections, but the really exciting material (the 'Results' section) is concertinaed into a few brief pages. To give some perspective, there is about 10 pages in the Discussion devoted to the slating of other published theories (I found this interesting reading but note that many of these critiques have been made elsewhere, either by Cavalier-Smith or by others) and around the same proportion of the 58 pages of text in this manuscript is dedicated to developing the novel ideas. This has two effects. One is that the novel material is deeply buried, and as a reader one has to work hard to extract the author's insights from such a lengthy composition. The second is that, on account of the brevity of the 'Results' one has to spend a lot of time figuring out exactly what Cavalier-Smith means. I am probably not alone in acknowledging that Cavalier-Smith has a broader knowledge of the subject matter than I, but it makes it hard to follow the reasoning when a number of points are assumed to be common knowledge and stated without some sort of background. While one might say I should have done my homework, I think much would be gained from acknowledging that the topic of this paper is of interest to a broad range of evolutionary and cell biologists, and providing the requisite detail would therefore be of value. In some cases the arguments are indeed well-supported and developed, but for other points there may be a greater degree of speculation, and the difficulty is that without a fair recollection of Cavalier-Smith's extensive canon, it may be hard to follow all the reasoning. This is a pity because, some points might therefore be mistaken as superficial speculation when they are in fact well-supported.

***Authors response: ****Only one bread slice (the introduction) was substantially thicker than the meat. That is because it provides background to help digest the meat. The referee requests more background for certain topics in the results section; the problem with such a broad field is that readers differ greatly in which parts of the background they really need; some may want more on some points and less on others than provided. I have amplified only parts of the introduction for which at least one referee seemed to need more information. Cutting some or inserting more might please some readers but irritate others: each can skim what to them is obvious and linger over what seems novel. I also amplified the meat section on the evolution of nucleocytoplasmic transport to clarify points raised by the next referee, moved the general section on megaevolution to the discussion so readers reach the meat faster, and made the results discussion of the origins of mitosis (now mentioned in the title), meiosis and heterochromatin more thorough with more background even though this needs some revisiting of arguments and evidence discussed previously. A diatribe is defined as bitter and abusive; my discussion has much robust but carefully reasoned criticism of arguments, including some of each referee, but is neither bitter nor abusive so is not a diatribe. I would welcome similarly robust, reasoned and discriminating criticisms of my ideas, much preferable to their being ignored or uncritically accepted. I thank all referees for their comments; more such dialogue is needed in this field*.

What follows are a few specific comments or questions concerning ideas presented:

p7, "The eubacterial ancestor of neomura could not have been a negibacterium with two bounding membranes, but was a posibacterium with a single surface membrane, like neomura; probably an actinobacterium, but possibly an early intermediate between Endobacteria and Actinobacteria".

Here I wish to request a clarification. This statement builds on two points. One is that archaea and eukaryotes are sister groups. The other is that the neomura (archaea + eukaryotes) evolved from within the bacteria. The view that neomura evolved from bacteria is based on an argument Cavalier-Smith has made before (see in particular Biology Direct 2006, 1:19), and is where the statement 'probably an actinobacterium' comes from. This gives the impression that neomura (archaea + eukaryotes, and ignoring any genetic contribution from mitochondria) evolved from the actinobacterial crown. I would like to ask whether the author's statement should be taken to mean this is a possibility, or whether actinobacterial-neomuran affinity refers to a split before the most recent common ancestor of actinobacteria (i.e. a stem actinobacterium).

***Author's reply: ****I thought there was no compelling evidence either way, so stated the point thus, and simply referred to my previous discussion of the uncertainties *[13]. *The radical changes in so many genes that caused the neomuran revolution make it exceedingly difficult to answer this question using gene sequence trees - more difficult than establishing the branching order of the eubacterial phyla (itself largely unresolved by sequence trees alone). However, reasons now specified in the introduction indicate that neomura are most likely sisters to crown actinobacteria, as shown in the more resolved revision of Fig. *1. *Insofar as the posibacterial ancestor of neomura must already have evolved phosphatidylinositol, sterols, and 20S proteasomes, it was a stem actinobacterium rather than an endobacterium (though it may well have had endospores as do the most divergent actinobacteria). Phosphatidylinositol can be considered a synapomorphy of actinobacteria that clearly differentiates them from Endobacteria, which unlike the other two has not been secondarily lost within the group. If, as previously suggested *[12]* that ancestor was a thermophile (****not ****hyperthermophilic), it might also have had a GC-rich genome*.

I also think it is worth me pointing out for those who might disagree with the bacterial origin of neomura that one does not need to accept all the steps preceding the archaeal-eukaryote split in figure 1 - for the purposes of following the novel arguments developed here the reader should concentrate on the three stages marked with stars.

***Author's reply: ****That is correct. The nature of the mechanistic and selective forces that led to the nucleus is logically independent of the phylogenetic origin of neomura (seemingly not realised by the third referee). However it is likely that destabilization of eubacterial structure assumed by the overall theory during the origin of neomura almost immediately prior to the origin of the eukaryote cell was of key evolutionary importance in facilitating the radical changes during the origin of phagotrophy. A close association in time between this putative destabilization and the divergent origins of both eukaryotes and archaebacteria and the probable rapidity of the transitions is a major part of the explanation of the absence of extant intermediates between the three domains; it helps explain why they are so phenotypically distinct. Moreover, readers also need to take on board the palaeontological evidence that eubacteria are much older than eukaryotes, combined with the phylogenetic evidence that archaebacteria are sisters rather than ancestral to eukaryotes and the absence of any compelling evidence that archaebacteria are as old as eubacteria because this is important for understanding the phylogenetic origin of the individual components of the eukaryotic host, especially those that are absent from archaebacteria. It is important to appreciate the major role that unsubstantiated assumptions, fashion, and the very name archaebacteria, rather than critical reasoning, has played in the prevailing view (in my view wrong) of the equal age of neomura and eubacteria*.

p23 'and possibly triggering the Neoproteozoic snowball earth'

and

'in the only really major explosive adaptive radiation prior to the Cambrian explosion'

and (on p25) 'Every gene probably duplicated many times in just a few days'

One factor that makes this paper frustrating to read is all the distraction. The above comments do not really seem necessary for presenting the central ideas. The first is a speculative aside that Cavalier-Smith has given elsewhere and does nothing more than derail the reader's concentration by throwing in an unnecessary spanner. The second is a qualitative statement of the throwaway kind, better suited to a narrative piece, and is again distracting. The statement from p25 is again terribly speculative, and, to my mind these types of statement (there are many more) detract from the strengths of the paper.

***Author's response: ****these are fair comments, reflecting our differing opinions of how one should present ideas; however, it would have been better to have said that such brief, less central statements may detract from ****some ****readers****' perceptions ****of the strength of the paper. They do not reduce the ****actual ****strength of the central arguments, which stand on their own merits. I retain them to alert readers to these possibilities, the last one as a deliberate irritant to stimulate readers to consider that key changes can be much faster than often assumed. Because they are peripheral and/or speculative it is not useful to amplify them here*.

I like the discussion of Simpson's writings, but think the author might get to the last paragraph, and the last sentence of the last paragraph ('These are just descriptive terms; they do not invoke special mechanisms beyond mutation and selection'), a little earlier. I found that, having an idea of what the author was actually saying made the second reading of the paper much easier; the first time through I was heavily distracted by all the strong statements about microevolution, e.g. p4, '.dramatic innovations like the origin of the nucleus that are incomprehensible by just extrapolating normal microevolutionary changes'. I think that one does have to be careful with this sort of blanket statement since 'microevolutionary processes' has in the past been very broadly defined. Suffice it to say, it is clear with careful reading that Cavalier-Smith is not invoking special ad hoc mechanisms to account for the origin of the nucleus, so this is perhaps a case of smoke without fire, though with statements such as 'the origin of eukaryotes was an intrinsically abnormal event, not one understandable simply by extrapolating the trivial tinkering that occupies most evolution. To understand it we must invoke something quite exceptional' (p25), it is easy to think this is the case.

This melodramatic wordsmithery is followed by a brief list of four important items (note to the author: only three are listed): preadaptation to phagotrophy, 'novel selective forces' that phagotrophy brings (these are left unexplained), and disruption to cell division and DNA segregation as a consequence of phagotrophy. This at least tempers the hyperbole immediately preceding it. What follows that is an interesting discussion of preadaptations that the ancestor of eukaryotes must have possessed: a single membrane, loss of the murein cell wall and several cellular attributes (large cell and genome sizes, diverse lipids, a secretome and a facultatively aerobic/anaerobic metabolism). Cavalier-Smith argues that Actinobacteria appear to carry these preadaptations. In this view, the lineage leading to archaea secondarily lost these preadaptations.

The points concerning preadaptation are essential for Cavalier-Smith to place this work within the wider context of his own ideas on the neomuran revolution, and are interspersed with slicing critiques of others' proposals for the cellular nature of the host that engulfed the mitochondrial ancestor (i.e. an archaeal host or a mycoplasma). What is most important to my mind is that identifying these preadaptations provides important cell biological insight into the origin of the eukaryote cell, regardless of the specifics of the actinobacterial-eukaryote affinity.

***Author's response: ****These perceptive comments highlight the difficulty of getting over the idea of radical differences in scale, style, and speed of megaevolution compared with day to day microevolution without exaggerating or underplaying the importance of the descriptive distinction, and without being misunderstood because of past exaggerations or mistaken claims for radically different mechanisms or contradictions that do not exist that make many reasonably wary of such distinctions. It is pleasing that nonetheless you understood the essence of what I tried to say. I have now placed the last paragraph as the second paragraph with some modifications that may help other readers understand my message more easily. Emphasizing the dramatic contrast between megaevolutionary events and most evolution is not hyperbole; it is an essential truth. It is the extravagant exaggeration by some students of microevolution to the effect that 'all evolution is just changes in allele frequency' that is truly hyperbolic and wrong. The origin of eukaryotes is a prime example where the origins of hundreds of new genes and protein domains and of dozens of radically new cellular structures was far more important than changes in the frequency of alleles of existing genes, and thus involves considerations entirely outside the scope of standard population genetics, which does not consider cell structure or unique events at all. The more general parts of this discussion are now moved to the discussion*.

I have three questions for the author concerning the evolution of chromosomes from a circular to linear form (p33):

1. What do you make of the multiple secondary emergences of linear chromosomes among bacteria [386]? The constraints you raise ('the need for replication forks to converge on a single terminus and signal completion', p33) do not seem to apply to these cases.

***Author's response: ****These examples show that some bacteria can evolve linear chromosomes if they solve the end replication problem, which can be done in several different ways (no bacteria use mechanisms homologous to telomeres; the spirochaete Borrellia, like some viruses, uses the hairpin mechanism I originally proposed for eukaryotes *[221], *and the actinobacterium Streptomyces uses terminal proteins like some plasmids or viruses). Thus the constraints against bacterial linear chromosomes are not absolute, but that does not mean that no constraints other than the end replication problem exist (the same must apply to mitochondria, where linear genomes have also evolved multiply; apparently plastid genomes never did). It would be interesting to study the control of replication termination and its coordination with segregation and division in each case of secondary origin of linear chromosomes. A thorough understanding of this should show either that the putative constraint I invoked is irrelevant or that these bacteria found novel ways around it or for some reason can better tolerate linear chromosomes. I doubt whether they would reveal an advantage for linearity, which I suspect is a functionless evolutionary accident driven by mutation pressure, but do not exclude the possibility of an advantage*.

2. The discussion presented regarding the consequences of phagocytosis includes the idea that, prior to this, there were strong constraints on the number of origins of replication. The relaxation of these constraints ('only a single replicon per chromosome so replication termination could directly signal division to occur directly between the only two daughter replicon termini') is thus something that is proposed to postdate the archaeal-eukaryote split. Sulfolobus species are known to have more than one origin of replication [387,388] - do you have an opinion as to why multiple origins are found in some archaea? - this observation doesn't obviously support your statement that additional origins would be 'stringently removed by purifying selection for >2Gy'.

***Author's response: ****Sulfolobus is an interesting exception to the general rule. In so far as I consider that Sulfolobus evolved well after eukaryotes, this important exception does not invalidate my statement from which you omitted the key word 'previously': my actual statement 'previously stringently removed by purifying selection for >2 Gy' remains true. As this exception is found in an archaebacterium, which unlike eubacteria have Mcm-related DNA synthesis initiation proteins (Cdc6 proteins) related to those of eukaryotes, it is clear that replication initiation was significantly changed during the neomuran revolution (I argue as a coevolutionary result of the origin of core histones). These changes might have preadapted both eukaryotes and archaebacteria to evolve multiple replicon origins, provided there is a selective advantage for this. In the case of eukaryotes I suggested that this selective advantage was to ensure that their much larger genomes were replicated by their much slower replication forks in a small fraction of the cell cycle. Though Sulfolobus has a rather small genome, its replication forks move an order of magnitude more slowly than in most prokaryotes *[387]. *With three origins replication takes 40% of the cell cycle; with only one it would take 120%, clearly impossible; without multiple origins the cell cycle would be markedly longer, a severe selective disadvantage. Thus the exceptional slowness of Sulfolobus replication forks imposed a novel selective force for multiple origins, as in eukaryotes (I would argue probably independently, though the mechanisms of its synchronous initiation of multiple origin control need elucidating in detail to test this). Sulfolobus has asynchronous replicon termination, which raises the same issue of coordination with division as linear chromosomes: understanding this better would also help test these ideas. It seems that in general in archaebacteria there is a long gap after replication termination before division and no archaebacteria are known to use the trick of successive initiations at single origins (at shorter intervals than total fork transit time) whereby Escherichia coli and other enterobacteria speed up DNA replication and allow cell cycles shorter than replication time. The absence of such mechanisms combined with slow forks would favour multiple origins. In these and some other respects *[389,390]* there are closer resemblances in cell cycle controls of archaebacteria with eukaryotes than with eubacteria; possibly when archaebacterial cell cycles are better understood, additional preadaptations for what are currently thought of as typically eukaryotic mechanisms will become apparent. If they do, such cell features would have arisen in the ancestral neomuran - and thus be neomuran not eukaryotic innovations. In Sulfolobus multiple origins clearly arose by gene duplication (two of the three are linked to cdc6 genes) *[387], *as I originally suggested for eukaryotes *[4]. *Why replication should be so exceptionally slow in Sulfolobus could be related to its exceptional environment (80C and pH 3) that may have led to unusually stable chromatin structure, likely to delay strand separation by DNA helicases. Overall this exception illuminates the rule and supports my interpretation of the reasons for it, which explain both the rule and this exception*.

3. Thermophily has previously been proposed to be a selective pressure favouring the emergence of circular chromosomes [391]. Earlier in the manuscript (p6), you state that the ancestral archaebacterium was subjected to massive gene loss (including genes such as MreB) during 'secondary adaptation to hyperthermophily'. Given the statement that the ancestral archaeon was not hyperthermophilic, it would be nice to hear the author's view on the proposal that Cenarchaeum symbiosum (and related mesophilic 'Crenarchaeota') be classified as a distinct phylum (Thaumarchaeota - [232]), one possible implication from that work is of course that the ancestral archaeon was not hyperthermophilic. I haven't discussed this with Forterre yet, but an ancestrally mesophilic archaeon could potentially kill the argument for circular chromosomes as a direct thermoadaptation (since Cenarchaeum has a circular genome).

***Author's response: ****You misinterpreted the significance of 'secondary' in my sentence. I did not state that the ancestral archaebacterium was not hyperthermophilic. As explained in detail before *[12]* I have consistently argued since 1987 that it was hyperthermophilic and that hyperthermophily was the key innovation involved in the origin of the special archaebacterial lipids. Secondary means only that the ancestral neomuran and its eubacterial ancestors were not hyperthermophilic. Thus in the context of the whole tree of life ancestral archaebacterial hyperthermophily is secondary. I have never favoured Foterre's argument for thermophily as an explanation of the circularity of the chromosome as I have never accepted that archaebacteria are as old as eubacteria, which also mostly have circular chromosomes and are mostly non-thermophiles, which means that circularity evolved prior to the last common ancestor of all prokaryotes, for which there is no reason to assume hyperthermophily or even thermophily. Forterre's idea of the origin of prokaryotes by reductive evolution of eukaryotes is mechanistically totally implausible (nobody has ever suggested plausible intermediate stages or how you could undo the eukaryotic endomembrane system or cytoskeleton, still less how the first cell could have evolved it) and refuted by the fossil record, so his 'explanation' of circular chromosomes was a non-starter*.

*Your question about Cenarchaeum has a phylogenetic and a taxonomic aspect. Taxonomically I oppose making it a distinct phylum, just as I oppose cren- and euryarchaeotes as distinct phyla, for their phenotypic disparity is too small to justify a rank above subphylum. There have been too many unmerited designations of new phyla among bacteria based just on sequence trees rather than phenotype. Another unmerited phylum ranking is the hyperthermophilic Korarchaeota *[392]. *It would be more appropriate to make thaumarchaeotes and korarchaeotes new crenarchaeote orders, possibly classes. Ranking is a subtle thing and bacteriologists underuse the intermediate ranks of class and subphylum compared with order and phylum (technically division), yielding unbalanced and less useful classifications*.

*Phylogenetically there are two contradictory positions for Cenarchaeum: as sister to hyperthermophilic crenarchaeotes as on the 2-gene rRNA tree of Brochier-Armanet et al. *[232]* and both protein trees of *[393]* and as sister to all other archaebacteria as in the 53 ribosomal protein tree of *[232]. *It was unwise to assume as they did that the latter is the correct topology, as Cenarchaeum is represented only by one lineage and its position on the tree could reflect systematic bias, not true history. Indeed the EF2 tree and one based on 35 ribosomal plus 3 RNA polymerase proteins give strong support to Cenarchaeum being sister to hyperthermophilic crenarchaeotes. Furthermore the 16S-23S rRNA tree including Korarachaeum gave unequivocal support to it and Cenarchaeum both being the deepest branching members of the crenarchaeote clade. The latter trees based on a greater diversity of different proteins and including also a korarchaeote (unlike *[232]) *are more likely to be correct than the ribosomal protein tree of *[232]. *Interestingly the 38-gene tree places Cenarchaeum and 'Korarchaeum' as sisters with good support and this clade as sister to the traditional thermophilic crenarchaeotes with 100% support. If this is correct then your question would be irrelevant, as the conclusion that the cenancestral archaebacterium was hyperthermophilic would remain, as it would be most parsimonious to suppose that Cenarchaeum, like several euryarchaeote lineages, was secondarily mesophilic (it could also be sensible to group Cenarchaeum and Korarchaeum in one new class). I agree with Brochier-Armanet et al. that the characters shared by Cenarchaeum and euryarchaeotes are likely to be ancestral to archaebacteria and were probably lost by hyperthermophilic crenarchaeotes, but if such characters are indeed ancestral one cannot use their presence in Cenarchaeum as a reason for preferring one topology over the other. If Cenarchaeum is sister to all other archaebacteria, which seems to me unlikely in the light of *[393]* this would make their ancestral state harder to deduce; Brochier-Armanet et al. candidly admit that one could not confidently decide whether the cenancestor was hyperthermophilic or mesophilic just from the topology *[232]; *that is because a hyperthermophilic ancestry would involve only one more reversion to mesophily than the several we are confident of in euryarchaeotes; thus it is not so unlikely that we can reasonably reject it purely for parsimony. Even in the unlikely eventuality that Cenarchaeum is truly the deepest branching archaebacterium it would remain more likely that the cenancestor was hyperthermophilic, as that more satisfactorily explains the origin of their lipids and reverse DNA gyrase*.

*Forterre's explanation of the origin of reverse gyrase as a chimaera of two eubacterial proteins strongly shows that eubacteria are indeed ancestral to archaebacteria and neomura, as all the most compelling evidence congruently indicates. The trees and genome data of *[393]* confirm that erecting Korarchaeota as a separate phylum based on bad and misleading 16S rRNA trees (an all too common practice for bacteria) was premature and unwise, and together with the genome data for Cenarchaeum make the same point for it; neither is more radically different from other crenarchaeotes than the very diverse euryarchaeotes are from each other; moreover they show that the differences between crenarchaeotes and euryarchaeotes have been exaggerated; many now clearly stem from secondary losses in the cenancestor of the traditional hyperthermophilic crenarchaeotes, just as I argued *[12]. *It is increasingly clear that archaebacteria were ancestrally hyperthermophilic, evolved from eubacteria, that both subphyla generated secondary mesophiles, and that reductive evolution by multiple gene losses has been far more important in their origin and diversification than is yet widely accepted. But there is absolutely no evidence for the generation of prokaryotes by the loss of eukaryotic organelles. I would rank Thaumarchaeota and Crenarchaeota each as classes, not phyla*.

p39: 'Because selfish DNA, especially transposons and endogenous retroviruses, spread much more slowly in sexual than in clonal populations, a sexual protoeukaryote would have suddenly been inundated by such genetic parasites.' There is an error here: presumably this sentence was supposed to read 'spread much more slowly in clonal than in sexual populations'. Here I think it would be an oversight not to cite the seminal paper by Hickey [280]. I am not aware of 'spread' in clonal systems, though there may be 'persistence' of some types of mobile element. Arkhipova & Meselson [394] showed loss of retroelements during the evolution of asexuality in Bdelloid rotifers, which makes sense given these elements propagate through replicative transposition. It seems that DNA transposons persist, and, given that they can transpose by conservative mechanisms, this may well be a case persistence rather than slow spread. It is noteworthy that bacterial transposons are primarily conservative, but can occasionally transpose by replicative means - overall there doesn't seem to be much evidence for proliferation (at least judging by prevalence in prokaryote genomes).

***Author's response: ****It was an inversion error, now corrected. I inserted reference to Hickey *[280]* and also to my paper that was probably first to argue that non-infectious selfish DNA cannot spread easily in clonal populations *[279], *and which directly stimulated Hickey to show that it can easily in sexual ones. I originally cited neither because this is old established history, just as one does not cite Darwin every time one mentions evolution or Watson and Crick every time one mentions a DNA double helix. With respect to bdelloids, they contain many more transposons than previously thought *[395], *indicating that transposons can spread even in clonal asexuals by lateral gene transfer (e.g. by viral infection), for which there is also good evidence in bdelloids *[396]* (or that bdelloids are not strictly asexual). This does not alter the fact that evolving sex added an extra easy way for transposons to spread*.

Gáspár Jékely

Max Planck Institute for Developmental Biology, Tübingen, Germany

This paper presents a critical review of various models on the evolution of the nucleus along with a long argument that intracellular coevolution is the key to understanding eukaryote origins, as well as a scenario on endomembrane and nucleus evolution. I find this a very detailed and insightful synthesis. Below I comments on certain parts of the paper, which relate to some cell biological aspects of the author's model. I focus on those parts where the author's scenarios disagree with some of the scenarios I had proposed on the origin of secretory membranes, predation and the nucleus. Hopefully this discussion will help to improve our models and also help to recognise the merits and weaknesses of the somewhat contrasting scenarios.

Comments about the small GTPase tree

You challenge some of my earlier suggestions that were based on the phylogeny of eukaryotic small GTPases [65]. You write, that "It is evident from other work that the Arf branch is the longest among the eukaryote paralogues and that the small GTPase tree is essentially unresolved at its base [83]; therefore rooting of the tree with the far longer eubacterial outgroup could have wrongly attracted it to the base." Ref. 83 does not show that Arf is the longest branch, and in the tree shown in this paper only Ran is unresolved. The small GTPase tree is also resolved at its base in other analyses (see also my Bayesian tree in [ref. [67]]). Besides, I didn't claim that Arf came first, but that the first split separated Sar1/Arf/SR? from the rest. Sar1/Arf/SR? also forms a distinct group in [ref. [87], which paper suggests independent origin for this branch and the Ras/Rab/Rac/Ran branch. Looking back at my paper [65], I realised that you probably misinterpret the numbers at the nodes of the tree in my Fig. 1[65], which solely indicated the nodes, to simplify discussion about them, and not branching orders.

You also write, that "It is as unwise as for other paralogue trees to assume that the rooting is accurate." Rooting with the closest eukaryotic paralogs, the trimeric G-protein alpha subunits, also gives the same position for the root [67].

Further down you write: "The worst argument, however, was that the Arf-1 branch is involved in secretion and the Rab one in phagocytosis, and to combine these two bad arguments to conclude that exocytosis evolved before phagocytosis [65]." I disagree. It can be safely deduced that the Sar1/Arf/SR? branch ancestrally regulated secretory membranes. Sar1 and SR? still exclusively do this, and Arf1 has indispensable functions in Golgi. The ancestral function is not so clear for the other branch. Later you write, that "almost certainly endocytosis and exocytosis coevolved, recruiting from a common pool of enzymes when assembling their toolkit." I agree with this, but I still think that putting whole-cell phagocytosis first is cell biologically unrealistic. There had to be some preadaptation also in the membrane trafficking system to allow complete cell engulfment.

You also write, that "Thus was born the primary divergence between ER outwards secretory traffic and plasma membrane inwards endocytic traffic seen in GTPase [65] and SNARE [89] trees." Funnily, this is the same over-simplification that you just criticised above as my worst argument (in ref [65]).

***Author's reply: ****We both agree that 'endocytosis and exocytosis coevolved, recruiting from a common pool of enzymes when assembling their toolkit' and also that it is not easy to infer whether the Rab branch was originally involved in phagocytosis or exocytosis. That being so, it is not safe to infer what was the ancestral role in the common ancestor of both branches. The difference between us may not be that huge. I agree that there must have been much preadaptation in several respects not least with respect to surface membrane properties. I also agree that on the grand scale of things secretion preceded phagocytosis as it is done by all prokaryotes whereas phagocytosis is derived. However secretion and exocytosis are not the same. My 'oversimplification' and your 'worst argument' are**** not ****the same - both for this reason and also because my statement about SNAREs is obviously not the same as yours about Rabs. You argued from the Rab tree that exocytosis preceded phagocytosis. I argued that secretion (which ancestrally did not involve exocytosis) preceded both and that primitive phagocytosis marginally preceded exocytosis, but that well-developed exocytosis and phagocytosis must have coevolved; even my statement about both trees mentioned only primary divergence and did not involve a claim that exocytosis came first. With respect to 'putting whole cell phagocytosis first' my recent discussion of the origin of the endomembrane system argues that predation was the primary driving force for eukaryogenesis but that some novel features of the endomembrane system evolved during an earlier phase of extracellular digestion prior to phagocytosis itself, but that (as argued earlier) cell ingestion was what first generated internal membranes and thus membranes that could for the first time undergo exocytosis *[27]. *I refer the reader to that paper for details*.

Comments about the nature of the first endomembranes

You also disagree with my secretory membranes-first scenario, although the difference between our scenarios may not be so great as it seems from this criticism, and I think that our models will eventually converge. You step back a little in this paper, but in your recent paper [91] you postulated an early phase of membrane evolution, even discussing the possibility of some tubulation before phagotrophy, as I suggested in [65]. In ref [91] you also speculate on the primacy of ER-linked functions, such as surface secretion of membrane-anchored digestive enzymes and ERAD. In my scenario these evolved on the early membrane tubules, somewhat before total cell engulfment and were preadaptations to allow regulated secretion during phagotrophy and possibly membrane splitting/fusion. The prey might not have been exclusively cellular, as you also write [91], it could have been digested nutrients or viruses and these could have been digested/absorbed by a tubular endomembrane network (and I agree that ERAD is a very plausible mechanism [91]). I would argue that the phagocytosis machinery, which is based on actin, Rac and pseudopodia, evolved independently, although always functionally linked (e.g. via digestive enzyme targeting) to the secretory membranes, which is rather based on microtubule motor, tubules and vesicles. Your figures are still not updated to include these tubules, which would emanate from the prey vesicle in your scheme, or directly from the PM, before total engulfment, according to my scheme.

You also write, that "So we should not ask 'did phagocytosis or exocytosis evolve first?': both evolved together, with phagotrophy being the entirely novel selective advantage, as De Duve [90] and Stanier [2] first argued." Actually Stanier in ref [2] wrote 'endocytosis', and De Duve expressed views, which are more similar to the enzyme secreting, food capturing tubules I described in ref [65], rather than to whole-cell engulfment. According to De Duve [90] endomembranes evolved by "infolding of the cell membrane, allowing the formation of internalized extracellular pockets into which captured food and secreted enzymes were trapped together ... If this hypothetical reconstruction is correct, then the decisive event may have been simply the progressive spreading, with concomitant infolding, of a membrane already adapted for secretion and absorption." Assuming that the food was not cellular, it is easier to imagine such (tubular) invaginations, generated by primitive membrane bending coats and molecular motors pulling on membranes [65]. These membranes could have had both secretory and absorptive function. In these membrane invaginations and networks fusion and fission could evolve, all preadaptations for total cell engulfment. Otherwise I cannot imagine what your prekaryote did when it first engulfed a whole bacterium and topologically separated the phagosome from the plasmamembrane. How did it re-fuse the vacuole if no membrane fusion machinery was present? How did the cell do it on a regular basis to earn a living? I would like to emphasize, that my model also posits that eukaryogenesis was driven by the evolution of predation, but I incorporate some intermediate steps before total cell engulfment, that also make the scenario cell biologically more plausible. The selective logic and a possible social scenario for the evolution of predation is described in (Biol Direct. 2007 Jan 19;2:3.).

***Author's reply: ****Our thinking is certainly much more similar than either is to some deeply contrasting views that seem to ignore cell biology, especially in that we both see membrane budding and fusion as the most crucial innovation in eukaryogenesis and give a central role to novel selective forces and mechanisms stimulated by predation. You also rightly guessed that the present paper and figures were prepared long before my recent paper that proposes a predatory and partially extracellular digestion phase prior to ingestive phagocytosis *[27]. *That modification to my earlier ideas was made because, like you, I think that some degree of preadaptation is essential in explaining complex innovation and because my proposal also neatly explains many aspects of the evolution of the ERAD machinery that was unknown when I first thought about the origin of phagocytosis. I imagined that some refusion of food vacuoles with the plasma membrane could have occurred accidentally because of an inherent tendency of membranes to refuse, but grant that this would probably be inefficient and that an evolution of phagocytosis without a simultaneous improvement to exocytosis could hardly have occurred; I pointed that out long ago *[11], *and thus have never thought that phagocytosis evolved in detail prior to exocytosis. Thus without a very rapid improvement of refusion by the origin of specific membrane fusion proteins, there would have been a severe bottleneck. This would have given an extremely strong selective advantage to the evolution of exocytosis, stronger I think than in your scenario. The intermediate where I now postulate both were perfected is assumed to have 'made its living' initially by extracellular breakup the cellular prey and import of the proteins, so the transition to engulfing whole cells would make absorption of proteins more efficient and thus not be as radical a change in way of 'earning a living' as your question implies; complete internalization would also allow the better exploitation of energy rich lipids. In a chicken-and-egg problem like this it is more likely that exocytosis and endocytosis evolved together rather than either developed alone. I did not complicate the present figures by introducing tubulation because this paper focuses on the later origin of the nucleus, the necessarily prior origin of endomembranes having been discussed in detail in *[27].

*I am not against membrane tubulation per se as an intermediate stage; the difference between us is that I invoked it to improve the efficiency of absorption, as did de Duve, whereas you did so to increase the efficiency of secretion at the cell surface, which is less plausible to me. If as you now say such invaginations 'could have had both secretory and absorptive function', we are indeed coming closer; what I objected to was singling out secretion as their primary one. Until at least some membrane budding to internalize membrane had occurred, there would have been no internal membranes that could be externalized by exocytosis or from which exocytotic vesicles could bud and later fuse with the cell surface. Thus exocytosis must have followed ****some ****membrane internalization; such internalization would be most simply initiated by a rudimentary form of endocytosis/phagocytosis. If so, the rudiments of phagocytosis at least briefly preceded the rudiments of exocytosis. With respect to history, you misinterpret Stanier's use of 'endocytosis'; at the time he wrote the term included both phagocytosis and pinocytosis (see p. 9 of his paper); it was specifically invented to embrace both; its much more recent use to refer mainly to receptor mediated micropinocytosis in contrast to phagocytosis is a complete distortion of its original meaning. As you probably know, Stanier went on to say (p. 27): 'the capacity for endocytosis would have conferred on its early possessors a new biological means for obtaining nutrients; predation on other cells.' prior to discussing the impact of this on cell structural complexification. There is no doubt that he had in mind mainly phagocytosis rather than pinocytosis as the driving force, just as I did when proposing actin as the key molecular invention enabling phagocytosis and eukaryogenesis *[10].

Comment about the constriction of the NPC cylinder

About the early evolution of the NPC you write, that "Later this wide cylinder, allowing nucleocytoplasmic exchange by passive diffusion (Fig. 5a), was constricted to exclude ribosomes from the nucleus" A simple constriction of the proto-NPC cannot exclude ribosomes and other factors from the nucleus. For exclusion FG-repeats are needed. A constriction will only result in more limited diffusion, but not (size) selectivity. Ribosomes cannot be excluded this way, because they are assembled in the nucleus. What such a diffusion barrier could achieve is increased RanGTP levels in the nucleus and a sharper RanGTP gradient (as shown in ref [139]). Everything then follows from this, as nuclear transport evolves. An increase in localized RanGTP was probably the primary reason for constricting the proto-NE and NPCs.

***Author's reply: ****there is a misunderstanding here; the Figure citation relating to 'constriction' is 5b, which does have the FG repeats. I thought it was clear that in 5a where there are no FG repeats the ribosomes had free passage but in 5b when they are added they do not. To prevent others misinterpreting my meaning I changed 'constricted' to 'constricted by inserting the inner FG repeat ring'. I do not really understand the statement about ribosomes not being excluded because they are assembled in the nucleus; precursor ribosomal subunits are indeed thus assembled, but complete ribosomes with messenger are larger and generally only assembled in the cytoplasm and thus generally are excluded*.

You also write, that "possibly their first cargo was inner-membrane DNA-binding proteins [18], that would otherwise be impeded by the novel integral membrane Nups, unless from the start NPCs opened to let them cross the NPC NE domain" It is not clear that NPCs have to open to allow the crossing of transmembrane cargo. There are clear gaps between the NE and the NPC that may allow the passage of transmembrane proteins (Cell 1992 69:1133-41, Nature 2007 449:611-5, Science, 2008 322:1369-73).

***Author's reply: ****an interesting comment. However there is evidence that some integral membrane proteins are imported by karyopherins *[397]. *I suspect that my and your proposals about which were the first things to be transported may both be oversimplified guesswork, and that we may never be able to reconstruct with confidence the order in detail*.

Comment about the selective forces during the evolution of the nucleus

I am unconvinced by the kinetic argument for phase 2 of the evolution of the nucleus (the first steps in the evolution of selective transport). In ribosome assembly the rate-limiting step is rRNA transcription and maturation, and is not limited by the diffusion of ribosomal proteins, which is very fast. So even if the cell can concentrate its ribosomal proteins by active nuclear transport, it will not make more ribosomes. The anuclear cell can simply make the amount of ribosomal proteins needed, which will quickly diffuse to the site of ribosome assembly as the rRNA is made. Similarly, during nucleosome assembly the rate limiting step is replication, and concentrating histones doesn't help. So no cost is spared, but it is costly to drive transport by RanGTP. So evolving nuclear transport in order to concentrate ribosomal proteins and histones around DNA is not a good explanation. In my model [ref [139]] the evolution of nuclear transport is not simply about absolute, but relative concentrations (host ribosomal proteins relative to mitochondrial ones).

***Author's reply: ****As explained previously *[4,5]* but not reiterated here, compartmentation advantages centrally involve the lower cost of maintaining a high concentration of key molecule if they are excluded from the bulk of the cell. By largely excluding free histone, other nuclear proteins and ribosomal proteins from the cytoplasm, roughly 10-fold less of each protein is needed per cell cycle to maintain a given nuclear concentration than if they were evenly spread through the cell. That is a large economy. Thus it is not just a question of rate limitation as you assume. If replication were the rate-limiting step for nucleosome assembly why would there be so many different nucleosome assembly chaperones?*

Comments on the criticism of the ribosome chimerism model

You also criticize my model on the origin of the nucleus [139]. You write, that "First, there is no evidence that the mitochondrion evolved before the nuclear envelope" This I also acknowledge in the paper. However, you also note, that "We now see that the origins of mitochondria, nuclei, and cilia were virtually simultaneous. As each was a complex series of processes it is most likely that they overlapped in time, so disputing which was first may be meaningless." So, simply, we don't know the order. My theory is based on the assumption that the mitochondrium came slightly earlier, than the nucleus, but clearly after phagotrophy evolved. You also write, that "Secondly, it is not very logical to suppose that the transfer of genes could be tolerated with impunity for a period and then became a compelling force for evolving the envelope." In my theory the nucleus evolve to correct the bad effects of mutation pressure. It is the same logic as the one you apply to explain the origin of NMD: "Thus like splicing itself, and many genomic oddities like RNA editing [105], nonsense-mediated decay is a consequence of selfish DNA evolution; correcting the bad effects of mutation pressure, not positively adaptive." I say the same about the gene transfer ratchet of mitochondrial ribosomal proteins (slightly deleterious mutations fixed by drift), and when the harm is done, the problem is fixed by compensatory advantageous mutations as the nucleus evolves. Since there are 24 individual transfers, the harm is done in small steps, and compensation can also evolve in small steps.

You also write, that "If transfer were so strongly disadvantageous, it would be more likely either that cells with transferred genes would lose out in competition" It was probably not more strongly disadvantageous, than the shearing forces you postulate. This was collateral damage, caused by the evolution of other cellular features, which were either selected for or spread by drift, and then had to be fixed. Further on you write, that "Thirdly, just as for the intron harm theory criticised above, the early stages in nuclear envelope assembly could not have helped solve the problem" This is not a valid criticism either. Figure 2 and 3 in the paper [139] clearly show how the first stages could have reduced the problem. Actually, I can even give an explanation for the origin of the RanGTP gradient around chromatin, which can create a compositionally distinct region of the cytoplasm, which is not explained by invoking only shearing forces, as you do. Later you write: "Fourthly, in the final stages of evolution of nuclear import - increased selectivity - the benefit would have been the net benefit of excluding hundreds of different proteins from the nucleus and the positive import of hundreds of others." I agree, and I also mention [139] that in the final stages many transport processes and cargos had to coevolve.

***Author's reply: ****I am not against the principle of phenotypic correction of unavoidable mutation pressure being important in cell evolution, having invoked it several times myself, e.g. in the origin of spliceosomal introns, RNA editing, chloroplast DNA minicircles, and even with respect to ribosomal evolution as a consequence of the chimaera problem *[3,211,398,399]. *The difference between us is primarily over the likely selective disadvantages and advantages of the steps discussed. I apologize for not having properly appreciated your simulation studies, which I agree refute my original third objection to your ideas. I have therefore deleted it, making the fourth objection the third. I have also revised this objection to cast it in the form of strong support for your general thinking of how a degree of compartmentation could have been achieved prior to complete pores combined with scepticism over the special importance of avoiding chimaerism. We are both agreed in the central importance of compartmentation and coevolution for the origin of the nucleus, but I think it likely that there were many simultaneous benefits of compartmentation*.

Minor comments:

The conservation of EJC-dependent NMD in plants has also been demonstrated experimentally (EMBO J 2008 27:1585-95).

The structural similarity of COPII and NPC has recently been demonstrated (Science, 2008 322:1369-73).

***Author's reply: ****references added*.

The section "Origins of nuclear protein modification by sumoylation" does not really fit into the Discussion. Sumoylation is not mentioned in the main text. It would be better to include it into the Results section.

***Author's reply: ****moved*.

*Eugene Koonin*

'Intracellular coevolution and the origin of the cell nucleus and sex'

This is a very far-reaching, extensive discussion of a paramount problem in evolutionary biology, at least, with regard to the evolution of eukaryotes, the origin of the nucleus and sex.

The article is quite long but is overall an excellent read. Furthermore, I am very sympathetic with the succinct conclusion of the abstract on the importance of studying coevolution of different eukaryotic organelles for understanding eukaryogenesis. Yes, I think such a systemic approach is indeed key.

I am afraid, however, that this is where I have to stop with my comments. The text of the article, interesting as it undoubtedly is, does not appear to be an objective discussion of the problem, but rather a one-sided narrative that I am not inclined to analyze and criticize in detail. The show stopper to me is that the "painted picture" is based on several major assumptions that are accepted here as unquestionable but that I find either highly controversial or outright implausible. The highly controversial assumptions are the archaezoan nature of the proto-eukaryotes that was the host of the mitochondrial endosymbiont, that is, the assertion that this organism was a bona fide phagotroph that possessed the principal eukaryotic features such as the endomembrane system, the cytoskeleton, and the nucleus itself; and the bikont-unikont phylogeny of eukaryotes.

***Author's response: ****The core subject of this paper is not the ****origin of mitochondria***, *treated in detail elsewhere *[20], *but new proposals on the physical mechanism and selective forces causing the origin of the ****nucleus***. *These are entirely independent of whether or not the eukaryote tree is rooted between unikonts and bikonts, as the original version of this paper assumed, or not. Indeed, new evidence suggests that that rooting was mistaken *[9,55]* and that the root is instead within Eozoa, specifically between excavates and Euglenozoa (or possibly even within Euglenozoa) as explained elsewhere *[9]. *But this does not in any way affect the mechanistic and selective arguments proposed here, which are independent of the precise rooting. As I stated, no reasonable alternative alters the conclusion 'that the ****last ****common ancestor of all eukaryotes was a phagotrophic protozoan with nucleus, at least one centriole and cilium, facultatively aerobic mitochondria, sex (meiosis and syngamy) and dormant cyst with cell wall of chitin and/or cellulose, and peroxisomes'*.

*The referee inaccurately states that I asserted that the host for the origin of the mitochondrion was a fully developed eukaryote with a nucleus. I did not. I said it was ****either ****such a well-developed eukaryote ****or else ****a somewhat earlier 'intermediate stage' that already had evolved 'rudiments of phagocytosis and endomembranes'. My arguments as to the mechanistic cause of the origin of the nucleus depend (as do most scenarios for its origin) on the prior evolution of the endomembrane system, but are independent of whether mitochondria were acquired after the origin of the nucleus (as in the disproved archezoan hypothesis *[400]*)*, *before the origin of the nucleus (as Martin and Koonin postulated *[21]*)or temporally overlapping with the origin of the nucleus. My positive arguments for the selective forces that favoured the origin of the nucleus in successive stages are also independent of the relative timing of nuclear and mitochondrial origins. My negative arguments against the prior acquisition of mitochondria and of introns derived from them as being a plausible selective force for the initiation of the origin of the nucleus are also independent of the historical fact of whether the mitochondrion came before, after, or during the origin of the nucleus. Thus even were it true that the mitochondrion came first, I would not consider it plausible that the origin of spliceosomal introns was the primary selective cause of the origin of the nucleus. It is also misleading to refer to my present views as favouring an archezoan host, as for over a decade I have rejected my earlier hypothesis that there are extant primitively mitochondrial eukaryotes (Archezoa) as all putative Archezoa turned out to be secondary anaerobes with relict degenerate mitochondria*.

*To refer to the idea that the host for mitochondrial enslavement was a bona fide phagotroph as 'highly controversial' is misleading and tendentious. Unless you place the root within non-phagotrophic Plantae (which the single symbiogenetic origin of chloroplasts forbids) or Fungi (which much evidence contradicts), we can safely conclude that the last common ancestor of eukaryotes was a bona fide aerobic and sexual protozoan phagotroph with all standard eukaryotic organelles. That its earlier ancestor which engulfed the ancestor of mitochondria was also a phagotroph obviously cannot be stated with equal confidence. Nonetheless, as no free-living bacteria (=prokaryotes) have ever been shown to internalize a foreign cell and maintain it as a symbiont, the alternative assumption *[21]* that the host that thus internally enslaved an proteobacterium was a prokaryote is mechanistically implausible and much less justifiable than to suppose that the host was a phagotrophic prekaryote or protoeukaryote that regularly engulfed prey cells. Phagocytosis provides the easiest and most widespread mechanism whereby symbiotic cells enter host cells. Intracellular symbiosis is so widespread in eukaryotes mainly (not entirely) because of the existence of phagocytosis; billions of cells daily are thus taken up by free-living eukaryotes. Conversely there are no examples of free-living prokaryotes able to engulf other cells or harbour intracellular symbionts (the only known example of intracellular symbiosis within a prokaryote is a parasite of eukaryotes); thus there is no evidence that a free-living prokaryote did so even once during 3.5 billion years. It is therefore immensely more likely that mitochondria entered eukaryotes after the origin of at least a rudimentary phagocytosis. Furthermore there is no phylogenetic evidence that mitochondria were taken up before the origin of the nucleus. To assume that they were is phylogenetically gratuitous and to assume that the host was a non-phagotrophic bacterium is mechanistically ****extremely ****implausible. Therefore it is not scientifically sound to invoke the formal possibility that mitochondria came before phagocytosis in evolutionary explanations; unless new evidence or compelling arguments to the contrary are adduced, it is more parsimonious and more likely that phagocytosis preceded mitochondrial acquisition*.

*Evolutionary explanations should distinguish between mere formal possibilities and genuinely likely events based on known properties of organisms. A remote formal possibility is not a valid ****explanation ****of an historical fact if a simpler one based on known phenomena exists. Therefore it is the alternative invocation of an unknown, unprecedented mechanism for engulfment by a free-living prokaryotic host (e.g. by *[21]*)that is unwarranted and 'highly controversial', not the idea that at least a rudimentary version of phagocytosis had probably evolved prior to the origin of mitochondria, which is mechanistically much sounder and phylogenetically entirely acceptable*.

*Because there are no phagotrophic prokaryotes it is unlikely that phagotrophy would have long preceded the origin of the nucleus (if such organisms existed for millions of years there is no reason why they should all now be extinct as the niches would still be available), and because there appear to be no primitively amitochondrial eukaryotes (if such organisms existed for millions of years there is no reason why they should all now be extinct as the niches would still be available), it is equally unlikely that the acquisition of mitochondria long preceded that of the origin of nuclei. Therefore for some years, in contrast to my earlier, now disproved, archezoan hypothesis *[60,400,401], *I have considered that the origins of phagocytosis, mitochondria, and nuclei were most likely essentially contemporaneous *[3,27]*and that we are unlikely ever to be able to order the two latter with certainty in time. All these events may have occupied well under a million years *[27].

An implausible one, as far as I can judge, is the "neomuran" origin or archaea (archaebacteria, under the terminology used here). All these assumptions are accepted as statements of fact, largely, on the force of previous publications, without much elaboration. My understanding, however, is that the arguments in those publications were seriously flawed, so I cannot accept the assumptions.

***Author's response: ****I wish you had said what you think the flaws are so that we could see if your objections have any weight. As you agree with me that archaebacteria are holophyletic sisters to eukaryotes *[24], *I do not see how you can logically object to my deducing that characters shared by both groups were present in their common ancestor, whereas characters unique to each probably arose only in their individual last common ancestors. The latter means that with respect to lipids the ancestor of neomura had acyl esters and was thus eubacterial not archaebacterial in nature. From your previous writings, I assume that you do not like the idea that eubacteria are older than both eukaryotes and archaebacteria, but you ought to consider both the palaeontological and transition analysis arguments for the greater antiquity of eubacteria and if you disagree for definite reasons should attempt to refute them. I refer readers to the latest discussion by Valas and Bourne *[41]* on the position of the root of the tree of life, which concludes that available evidence most strongly supports my conclusion, and that the post-1977 mindset of so many microbiologists of assuming the antiquity of archaebacteria without proper evaluation of the evidence is probably mistaken.*

I did not present this view dogmatically but did so only briefly 'relying on previous arguments'. As the arguments in my previous papers on this topic all remain valid and have not been refuted, and as the logic of my explanation for the rooting of the tree (both mechanisms and selective forces) is valid independently of the rooting of the overall tree of life, about which I had few novel arguments, I simply cited those in my earlier papers. As the referee criticises my doing that and as the root position is important for another aspect of the origin of the nucleus (the phylogenetic origin of each key protein), I have now introduced two even more compelling new arguments from lipid evolution for the posibacterial ancestry of neomura and summarise the overall logic below (readers familiar with this can skip the next two paragraphs):

*First is the phylogenetic argument that archaebacteria are holophyletic sisters to eukaryotes, not their paraphyletic ancestors. I originally based that argument on (1) the view that the unique lipids of archaebacteria are a shared derived character of archaebacteria alone and that the acyl ester lipids of eukaryotes are an ancestral eubacterial character vertically inherited by the host from an ultimately eubacterial ancestor, plus (2) the palaeontological fact that eubacteria are much older than eukaryotes but no unambiguous fossil evidence exists that archaebacteria are any older than eukaryotes, together with (3) the view that there is no mechanistically plausible way that the far simpler prokaryotic cells could be secondarily derived from eukaryotes, which was congruent with the fossil evidence that prokaryotes, specifically eubacteria, are older *[4]. *This original argument was made somewhat less decisive by the demonstration that all extant archezoa are secondarily derived from aerobic mitochondriate ancestors, as this meant that the origin of the nucleus and mitochondrion must have roughly coincided, raising the formal possibility that the eukaryotic acyl ester lipids were derived by lipid replacement from the mitochondrial rather than the host ancestor *[31]. *Elsewhere I explained why that formal possibility is mechanistically and selectively extremely implausible *[3]. *At that time I introduced a new argument that three gene splits found in archaebacteria alone, which are unlikely to have been reversed in the last common ancestor of eukaryotes, are additional evidence for the holophyly of archaebacteria *[12]. *Two new arguments introduced here are that Martin's idea of host lipid replacement by the mitochondrion *[31]* cannot explain the origin of phosphatidylinositol or sterols; phosphatidylinositol was crucial for eukaryogenesis *[27]* but is totally absent from both archaebacteria and proteobacteria but is universally present in actinobacteria, the likely ancestors of neomura; and the enzymes for making sterols are very widespread in actinobacteria and were not acquired by LGT from eukaryotes. Thus neomura must either have evolved from actinobacteria or be their sisters as suggested here. The latest trees from the referee's own group based on 355 genes argue much more strongly for the holophyly of archaebacteria than did any previous trees *[24]. *Thus, despite conflicting/ambiguous but unconvincing evidence from some gene trees *[46], *evidence for archaebacterial holophyly is stronger than ever*.

*If archaebacteria are indeed holophyletic, one cannot justifiably infer that the last common ancestor of archaebacteria and eukaryotes had any of the characters that are unique to archaebacteria or any that are unique to eukaryotes. Instead it is most parsimonious to suppose that this common ancestor had ****all ****the properties shared by archaebacteria and eukaryotes, plus ****all ****those shared by archaebacteria and eubacteria alone (i.e. prokaryotic genome and cell structure), plus ****all ****those shared by eukaryotes and the phylogenetically older eubacteria (i.e. acyl ester lipids, and dozens of genes ancestrally absent in archaebacteria such as Hsp70). In other words this last common ancestor of eukaryotes and archaebacteria (collectively neomura) was a ****not an archaebacterium***, *because on the most parsimonious assumptions it had acyl ester lipids like a eubacterium and prokaryotic cell structure. It resembled a posibacterial eubacterium because it had a single membrane not a double bounding envelope as do all other eubacteria (negibacteria). But it would be equally incorrect to call it a eubacterium as it would have had N-linked glycoproteins on its surface and presumably had already lost the peptidoglycan wall. Thus the last common ancestor of neomura was prokaryotic, but neither an archaebacterium nor a posibacterial eubacterium, but a missing link with some properties of each that was most probably derived from a posibacterium prior to the origin of either eukaryotic or archaebacteria-specific characters. That is why it is misleading to use modern names of extant groups to label such inferred intermediates; better call it an ancestral neomuran. The ancestor of the neomuran clade prior to the replacement of the peptidoglycan by glycoprotein and the origin of histones was a eubacterium, specifically a stem actinobacterium that still retained some key endobacterial properties that persist in neomura*.

*As the referee does not give any reasons why these arguments for a posibacterial/neomuran origin of archaebacteria are 'implausible' or 'deeply flawed' or cite any from the literature (I am aware of none that have been clearly laid out) I cannot do more to defend them than I did in previous publications. The referee does not mention any better explanation of the ancestry of Archaebacteria than that given previously *[4,12]. *The widespread assumption that they came from a mythical 'progenote' devoid of specific properties *[37], *which he presumably shares, is no more informative or better based than the idea of special creation*.

*One important discovery from the referee's research *[402]* illuminates the nature of the cenancestral neomuran further. They have shown that crenarchaeote and korarchaeote archaebacteria possess actin-like proteins that are more similar to actin and actin-related proteins (Arp 2 and 3) than are eubacterial MreB and ParM proteins. This implies that certain key changes that differentiate actin from MreB (and related eubacterial proteins ParM and MamK) occurred in the neomuran ancestor prior to the divergence of eukaryotes and archaebacteria. It is reasonable to suppose that these were associated with the loss of the peptidoglycan wall and the development instead of an improved internal cell skeleton for osmotic protection and mechanical stability. This finding does not alter the fact that the gene duplications that generated actin and the related Arp2/3 which nucleate its polymerization and mediate branching took place ****after ****the pre-eukaryotic lineage diverged from archaebacteria. This is consistent with the argument that this major step in actin evolution was associated with the origin of phagocytosis *[6,27]. *Thus archaebacteria lack this key feature of the actin skeleton; a three dimensionally cross-linked actin gel remains unique to eukaryotes. It was misleading to write of the archaeal ancestry of actins *[402]* as the tree for their actin-related proteins provides no evidence that crenarchaeotes are ancestral to eukaryotes; instead they appear as their sisters. Thus the novel features shared by actin/Arp2/3 and crenarchaeote actin-related protein (notably large related insertions near the C-terminal) are most parsimoniously interpreted as having evolved in the ancestral neomuran. Their absence in euryarchaeotes can be attributed to secondary loss. Very likely during the neomuran burst of gene duplication of MreB/actin-related proteins a number of paralogues was generated; one would have been ancestral to actin/Arp2/3 and crenarchaeote actin and a more conservative one to the methanogenic euryarchaeote MreB. Additional paralogue loss within crenarchaeotes can explain the findings of Yutin et al. *[207]*more simply than lateral transfers and protein replacements that they invoke between crenarchaeotes and korarchaeotes*.

*Their assumption of lateral gene transfer of MreB from eubacteria to methanogenic archaebacteria is also entirely unnecessary if one accepts the rooting of the tree of life within eubacteria *[4,6,12,13,40,47,48,50,381]; *vertical transmission from posibacteria to archaebacteria alone would suffice. Their assumption of lateral transfer to Thermoplasma is a bit more plausible because such transfer may be inherently more likely for a plasmid encoded paralogue like ParM than for MreB, but is also probably unnecessary. It is not obvious that its placement on the tree within the ParM/StpA clade is correct; I suspect it is a long branch artefact (LBA): a strong divergence in Thermoplasma actin-like protein caused by the loss of the eukaryote cell wall and ensuing selection for a better internal skeleton would have made it so divergent from its ancestral type (whether MreB or the crenarchaeote/actin paralogue) that it might not group correctly with them. It is evident from the tree that ParM evolves much faster than MreB. The alignment indicates that Thermoplasma protein is very idiosyncratic compared with all others in respect of both indels and sequence - just the sort of protein likely to be misplaced on trees; I do not think one can reliably distinguish between 3-5 possibilities: (1) that it is really sister to the crenarchaeote protein, but misplaced on the tree by LBA, (2) that it is really a ParM protein (descended either vertically from the neomuran ancestor or by LGT from another eubacterium) or (4) that it is an independent derivative of an MreB ancestor (descended either vertically or by LGT, but if the latter not necessarily from a eubacterium). Nonetheless the first possibility seems simplest*.

*Portrayal of the last common ancestor of eukaryotes by the referee's group *[402]* as a cell with a branched actin skeleton and mitochondrion but no nucleus or cilium was wrong. No matter where one roots the eukaryotic tree the last common eukaryotic ancestor had both nucleus and mitochondrion. Provided that one places the root somewhere between the five supergroups shown it is inescapable that it also had a cilium; that figure confused the last common ancestor that we can rigorously infer cladistically with a purely hypothetical intermediate between prokaryotes and eukaryotes. As Jékely pointed out, the implication that Rhizaria may lack phagocytosis is entirely erroneous; the group has two phyla: Retaria (Foraminifera and Radiozoa, in which every single one of the over 10,000 species feeds by phagocytosis) and Cercozoa in which all but a handful of the thousands of free-living species feed by phagocytosis. As referees Gribaldo and Jékely correctly argued, the conclusion that phagocytosis was absent from the cenancestral eukaryote and evolved several times independently in different eukaryotic supergroups is entirely unjustified. The authors conceded that the actin branching and control machinery was present in the last common ancestor of eukaryotes and thus accept that molecules of this core machinery like Arp2/3 which have not been detected in diplomonads must either have been lost or evolved dramatically in sequence beyond bioinformatic detection in a diplomonad ancestor. It is perfectly reasonable to argue that the same is true of other near universal eukaryotic proteins, e.g. Jékely also pointed out that the assumption that Ras was derived from mitochondria was not justified. Thus Yutin et al. *[402]*provided no new convincing evidence for their assumption that mitochondria arose before the nucleus, nor any reason for their calling the hypothetical primitively phagotrophic intermediate an archaebacterium rather than an intermediate pre-eukaryotic derivative of the ancestral neomuran. The first referee of the present paper shares my scepticism of their basic thesis, calling it 'specious at best'*.

Once again, this is interesting reading that reveals remarkable erudition of the author, a lot of interesting literature is cited, and the reader will benefit from a variety of exposition to an important and exciting research field, and possibly, from some of the ideas proposed in the manuscript. However, for reasons outlined above, I find that this manuscript does not call for serious discussion, so I offer none such.

***Author's response: ****A pity you did not give any reasons for your disagreement. It is important that those many who do not accept the phylogenetically older character of eubacteria compared with both eukaryotes and archaebacteria seriously consider (and not just ignore) the now quite compelling and varied evidence for this, which rests on a combination of palaeontological and phylogenetic evidence, both critically interpreted *[6,12,13,41]. *To establish an historical fact like the position of the root, we need arguments and evidence, not unjustified assumptions. Your assertion when reviewing another paper on the position of the root of the universal tree *[41]* that the sheer magnitude of the differences between archaebacteria and eubacteria is sufficient to place the root between neomura and eubacteria and to 'close' the debate on the position of the root and that you do not need to go into 'the minute details' of the evidence was phylogenetically unsound and not a good scientific attitude. Thank you, however, for being open-minded enough to recommend publication despite your disagreement. Please try to open your mind still further to the arguments and evidence *[4,6,12,13,23,41,403]* that refute this idea stemming from the extremely weak speculations of Woese and Fox *[404]. *Alternatively, please explicitly explain any errors in the reasoning concerning the roughly 20 polarizations from eubacteria to neomura *[12]* and 13 additional ones within eubacteria *[6,13]; *i.e. over 30 independent arguments that are mutually consistent evidence for the root being in Eobacteria. No similarly coherent integration of palaeontology and cell and molecular evolution has ever been presented in support of their now pervasive but almost certainly erroneous assumption (it is nothing more) that archaebacteria are as ancient as eubacteria*.
